# Dynamics and optimal harvesting of a fractional predator–prey system with fear and anti-predator mechanisms

**DOI:** 10.1371/journal.pone.0360316

**Published:** 2026-10-14

**Authors:** Mohan Kumar Govindan, Abhishek Kumar Singh

**Affiliations:** Department of Mathematics, School of Advanced Sciences, Vellore Institute of Technology-Chennai Campus, Chennai, Tamil Nadu, India; Federal University of Technology - Parana, BRAZIL

## Abstract

Wild prey are shaped by predators long before they are caught. Fear alone suppresses reproduction, physical refuges reduce exposure, and many species strike back directly at their attackers, imposing real costs on the hunter. To investigate the combined effects of these behavioral mechanisms, we develop a Caputo fractional-order predator–prey model incorporating fear-induced reproductive suppression, proportional prey refuge, active anti-predator retaliation, a Holling type II functional response, and nonlinear saturation harvesting. The fractional memory order accounts for hereditary effects in ecological interactions, with the classical integer-order system recovered as a special case. Fundamental dynamical properties of the model, including non-negativity, uniform boundedness, and existence and uniqueness of solutions, are established. All biologically feasible equilibria are characterized, and local and global asymptotic stability conditions are derived using Matignon’s criterion and Lyapunov function techniques. The analysis further reveals that the coexistence equilibrium can lose stability through a Hopf bifurcation induced by the fractional memory order, and a critical threshold for the onset of this bifurcation is derived. Numerical evaluation of the first Lyapunov coefficient provides evidence that the bifurcation is supercritical, leading to the emergence of stable periodic oscillations. Applying Pontryagin’s Maximum Principle, we derive a bang–singular–bang optimal harvesting strategy that improves discounted net revenue while maintaining ecologically sustainable population levels. Numerical simulations support the theoretical results and illustrate the combined influence of fear, refuge, anti-predator behavior, harvesting, and memory on ecosystem dynamics. The findings demonstrate that the fractional memory order acts as an effective regulator of ecological stability, providing a biologically interpretable mechanism for controlling oscillatory behavior that has no counterpart in classical integer-order predator–prey models.

## 1. Introduction

Predator–prey dynamics occupy a central place in mathematical ecology, and understanding the mechanisms that promote population persistence or drive extinction remains a fundamental challenge. The pioneering models of Lotka and Volterra [1] provided the first continuous-time mathematical description of predator–prey interactions, and subsequent developments have incorporated increasingly realistic ecological features, including logistic prey growth, saturating functional responses, harvesting, and environmental variability [2]. Among these advances, a particularly important realization is that predators can influence prey populations even in the absence of direct consumption. Zanette *et al.* [3] demonstrated that song sparrows exposed solely to predator vocalizations produced approximately 40% fewer offspring per year than control populations, establishing that perceived predation risk can itself act as a demographic force. Motivated by these observations, Wang *et al.* [4] introduced a fear-mediated predator–prey model in which predator presence suppresses prey reproduction, showing that strong fear effects can dampen population oscillations whereas weaker fear may destabilize coexistence. Subsequently, Zhang *et al.* [5] reported that the combined effects of fear and prey refuge can enhance system stability. Since then, fear-driven predator–prey models have been extended to delayed [6], stage-structured [7], and stochastic [8] environments, highlighting the growing importance of non-consumptive predator effects in ecological dynamics.

Beyond fear effects, prey populations employ several additional strategies that can substantially alter predator–prey dynamics [9]. Many species do not merely evade predators but actively retaliate through behaviors such as mobbing in birds and direct counter-attacks in fish and mammals, thereby imposing measurable fitness costs on predators. Incorporating such effects as prey-density-dependent predator mortality, Kumbhakar *et al.* [10] and Qin and Dong [11] demonstrated that anti-predator behavior can enrich the bifurcation structure and stabilize otherwise unstable coexistence states. The autonomous and nonautonomous study in [12] further revealed that the combined effects of anti-predator behavior and refuge use may generate bistability in ecological systems. The role of prey refuge is equally important. When a fraction of the prey population is protected from predation, effective predator–prey encounters are reduced, thereby weakening predation pressure. Both theoretical and empirical studies [13,14] indicate that moderate refuge levels can promote long-term coexistence, whereas excessive refuge may severely reduce predator persistence or even drive predators to extinction [5,15]. From a resource-management perspective, nonlinear saturation harvesting of Michaelis–Menten type provides a more realistic description than constant-yield or constant-effort harvesting because it captures diminishing returns at high prey densities and avoids the abrupt population collapses often associated with linear harvesting strategies [16–18]. Although the individual effects of fear, refuge, anti-predator behavior, and nonlinear harvesting have received increasing attention, their combined influence on ecosystem stability and management remains comparatively underexplored [7,17].

Classical ODE models assume that population dynamics depend only on the current state of the system [19]. In reality, however, ecological processes are often influenced by past conditions, including previous predation pressure, resource availability, and environmental disturbances. Caputo fractional derivatives provide a natural framework for incorporating such memory effects into mathematical models [20,21]. This approach has been successfully applied in a variety of biological settings, including epidemic dynamics [22] and ecological food webs [23,24]. Several studies have shown that memory effects can significantly alter system behaviour by suppressing oscillations, shifting Hopf bifurcation thresholds, and generating dynamical patterns that are not observed in classical integer-order models [25–27]. In particular, Li and Wu [28] established general conditions for the occurrence of Hopf bifurcation in Caputo fractional-order systems as the memory order crosses a critical threshold, and this framework has subsequently been applied to predator–prey models with various functional responses [24,29]. Consequently, the interaction between memory effects and ecological parameters can generate a substantially richer stability structure than that observed in corresponding integer-order models. The applicability of Caputo fractional derivatives extends well beyond predator–prey systems. Paul *et al.* [30] employed a fractional *SEIQRD* model to investigate COVID-19 transmission dynamics in Italy and demonstrated that memory effects can substantially influence disease spread. The same authors subsequently developed fuzzy fractional models for metabolic disease dynamics [31], highlighting the importance of hereditary effects across diverse biological processes. Related applications of fractional-order modelling to harvested predator–prey and epidemiological systems have also been reported in [32–34]. Collectively, these studies indicate that fractional-order modelling is not merely a mathematical extension of classical approaches but a biologically motivated framework for describing systems whose current dynamics are influenced by past states. These developments provide further support for the fractional-memory framework adopted in the present work. Related recent contributions include fear-driven fractional harvesting with stage structure [7], stochastic fractional predator–prey dynamics under climate forcing [35], and stability and bifurcation analysis of fractional models with memory effects [36–38].

From a resource-management perspective, determining harvesting policies that balance economic benefit with ecological sustainability remains an important objective. Optimal control theory provides a natural framework for addressing this problem by treating harvesting effort as a time-dependent control variable and maximizing net economic return over a finite planning horizon. Within this framework, Pontryagin’s Maximum Principle has become a standard tool for deriving optimal harvesting strategies in ecological systems [39,40]. Recent studies have shown that optimal harvesting policies in fractional-order predator–prey models can differ substantially from conventional constant-effort strategies and may lead to improved ecological and economic outcomes [7,17]. In particular, fear effects have been found to influence the economically sustainable yield, highlighting the importance of incorporating behavioural mechanisms into harvesting and management decisions.

Despite these advances, no existing study simultaneously incorporates fear-induced reproductive suppression, proportional prey refuge, active anti-predator retaliation, nonlinear saturation harvesting, and fractional memory within a single predator–prey framework. Existing studies typically consider only a subset of these mechanisms. For instance, fear-driven fractional models often neglect anti-predator behaviour and refuge effects, whereas harvesting studies either employ integer-order formulations or omit behavioural interactions altogether. Consequently, the combined influence of these ecologically important mechanisms on equilibrium structure, stability, bifurcation behaviour, and optimal harvesting strategies remains insufficiently understood. This gap motivates the development of the present fractional-order predator–prey model.

Motivated by the above observations, the present work addresses the following questions:

**(Q1)** How does the simultaneous incorporation of fear, prey refuge, anti-predator behaviour, and nonlinear harvesting affect the existence, feasibility, and stability of coexistence equilibria?
**(Q2)** Can the fractional memory order act as a bifurcation parameter, and if so, can a critical threshold for the onset of Hopf bifurcation be determined?
**(Q3)** What is the nature of the resulting Hopf bifurcation, and does it generate stable or unstable oscillatory dynamics?
**(Q4)** What harvesting strategy maximizes discounted economic return while maintaining long-term ecological sustainability?

As illustrated in Table 1, the proposed model extends the existing literature by integrating all five mechanisms within a unified fractional-order framework.

**Table 1:** Comparison of the proposed model with closely related studies. Ticks (✓) indicate that the feature is present; crosses (✗) indicate it is absent.

Reference	Fear	Refuge	Anti-pred.	Harvesting	Fractional	Key contribution
[4]	✓	✗	✗	✗	✗	First fear-effect prey–predator model
[5]	✓	✓	✗	✗	✗	Fear + refuge synergy
[24]	✓	✓	✗	✗	✓	Fractional fear + refuge model
[10]	✓	✗	✓	✗	✗	Anti-predator behavior, bifurcation
[25]	✓	✓	✗	✗	✓	Fear carry-over, fractional memory
[7]	✓	✗	✗	✓	✓	Fractional fear + optimal harvesting
[17]	✓	✗	✗	✓	✗	Nonlinear harvesting, bistability
[12]	✓	✓	✓	✗	✗	Anti-pred. + refuge, non-autonomous
**Present study**	✓	✓	✓	✓	✓	All five mechanisms, Hopf & PMP

The principal contributions of this work are summarized as follows:

- A novel Caputo fractional-order predator–prey model is formulated by simultaneously incorporating fear-induced reproductive suppression, proportional prey refuge, active anti-predator retaliation, and nonlinear saturation harvesting.
- Fundamental dynamical properties of the model, including non-negativity, boundedness, and existence and uniqueness of solutions, are rigorously established.
- The biologically feasible equilibria are characterized, and local as well as global stability conditions are derived using fractional-order stability theory and Lyapunov methods.
- A critical threshold for Hopf bifurcation is derived, demonstrating how the fractional memory order governs transitions between stable coexistence and oscillatory behaviour. Numerical evaluation of the first Lyapunov coefficient provides evidence that the bifurcation is supercritical.
- Pontryagin’s Maximum Principle is employed to derive an optimal harvesting strategy, resulting in a bang–singular–bang control policy that improves economic return while maintaining ecological sustainability.
- Numerical simulations are performed to validate the theoretical findings and to illustrate the combined effects of memory, fear, refuge, anti-predator behaviour, and harvesting on ecosystem dynamics.

The remainder of the paper is organized as follows. Section 2 presents the mathematical model and establishes the non-negativity, boundedness, and existence and uniqueness of solutions. Section 3 investigates the existence of biologically feasible equilibria and their stability properties. Section 4 examines the Hopf bifurcation and determines its direction and stability. Section 5 formulates and analyzes the optimal harvesting problem using Pontryagin’s Maximum Principle. Section 6 provides numerical simulations to illustrate and validate the theoretical results. Finally, Section 7 concludes the study and outlines possible directions for future research.

## 2. Mathematical model formulation

### Ecological assumptions and model derivation

Let *x*(*t*) and *y*(*t*) denote the prey and predator population densities at time *t*, respectively. The model is built on the following five ecologically motivated assumptions.

1. **Logistic prey growth with fear-induced suppression.** In the absence of predators, prey grow logistically at intrinsic rate *r* toward carrying capacity *K*. When predators are present, prey reduce their reproductive effort, so the effective birth rate becomes *r*/(1 + *ky*), where *k* > 0 measures the sensitivity of prey reproduction to predator density [4], capturing the “landscape of fear” effect [3] without requiring a direct predation event. This generates the term $\frac{r}{1+ky}\;x\;\left(1-\frac{x}{K}\right)$ in Equation (1).
2. **Holling type II predation with prey refuge.** Only the fraction (1−n) of prey is accessible to predators; the fraction n∈(0,1) shelters in a refuge [5]. Exposed prey are consumed at the Holling type II rate a(1−n)xy/[1+b(1−n)x]. This generates the predation loss $-\frac{a(1-n)xy}{1+b(1-n)x}$ in Equation (1) and the predator gain $\frac{ea(1-n)xy}{1+b(1-n)x}$ in Equation (2), where e∈(0,1) is the prey-to-predator biomass conversion efficiency.
3. **Nonlinear saturation harvesting.** Harvesting is modelled by the Michaelis–Menten term $-\frac{hx}{1+qx}$, where *h* is the maximum harvesting effort and *q* a saturation coefficient [16,17]. This saturates at high prey density, reflecting finite processing capacity, and avoids the abrupt collapses caused by linear harvesting. Real harvesting operations have finite capacity, so the harvest rate saturates toward *h*/*q* at high prey density rather than growing without bound as under linear harvesting *hx*; this avoids the abrupt depletion events that linear harvesting can cause during transient surges in prey abundance.
4. **Active anti-predator defense.** Prey species such as mobbing birds and retaliating fish impose a direct fitness cost on predators through counter-attacks [10,41], represented by −vxy in the predator equation, where v≥0 is the anti-predator behavior rate. The product *xy* reflects a mass-action encounter rate between the two populations. This generates the term −vxy in Equation (2).
5. **Caputo fractional-order dynamics.** Population dynamics depend not only on the current state but on cumulative histories of predation pressure, resource availability, and learned behavior. The Caputo derivative ${\;}^{C}D^{\alpha }$, α∈(0,1], encodes this hereditary memory through its non-local kernel: α=1 recovers the classical ODE, while α<1 introduces progressively longer memory [20]. Replacing $\frac{d}{dt}$ with ${\;}^{C}D^{\alpha }$ in Equations (1)–(2) is the only structural change from the integer-order system.

Under assumptions (1)–(5), the prey–predator dynamics are governed by the following Caputo fractional-order system:


$$\begin{array}{cc} {\;}^{C}D^{\alpha }x & =\underset{\text{fear-reduced logistic growth}}{\underbrace{\frac{r}{1+ky}\;x\;\left(1-\frac{x}{K}\right)}}-\underset{\text{Holling II predation}}{\underbrace{\frac{a(1-n)xy}{1+b(1-n)x}}}-\underset{\text{nonlinear harvesting}}{\underbrace{\frac{hx}{1+qx}}}, \end{array}$$ (1)

$$\begin{array}{cc} {\;}^{C}D^{\alpha }y & =\underset{\text{predator growth from prey}}{\underbrace{\frac{ea(1-n)xy}{1+b(1-n)x}}}-\underset{\text{natural mortality}}{\underbrace{dy}}-\underset{\text{intraspecific competition}}{\underbrace{my^{2}}}-\underset{\text{anti-predator cost}}{\underbrace{vxy}}, \end{array}$$ (2)

with initial conditions $x(0)=x_{0}\geq 0$, $y(0)=y_{0}\geq 0$, and α∈(0,1]. Setting α=1, *k* = 0, *n* = 0, *v* = 0, and *h* = 0 recovers the classical integer-order Holling type II Lotka–Volterra predator–prey model, confirming consistency with the classical ecological framework. A schematic overview of the five-mechanism model structure is provided in Fig 1.

**Fig 1:**
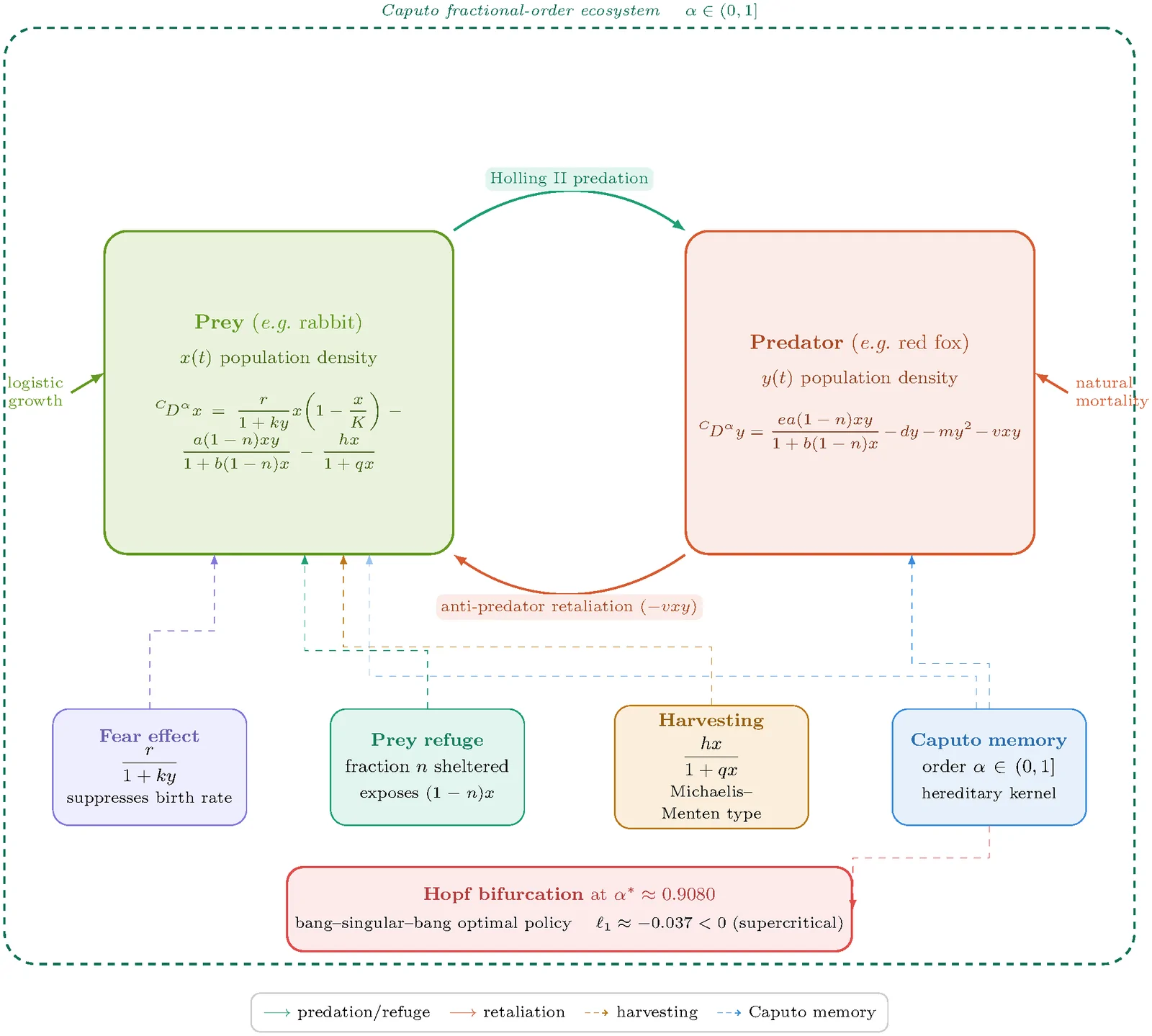
Schematic diagram of the five-mechanism Caputo fractional predator–prey model with fear, refuge, anti-predator retaliation, nonlinear harvesting, and memory order α governing a supercritical Hopf bifurcation at $\alpha^{*}\approx 0.9080$.

**Remark 1** (Biological basis and dimensional compatibility of the Caputo operator). *The replacement of* $\frac{d}{dt}$ *by the Caputo operator* ${\;}^{C}D^{\alpha }$ *is motivated by three well-documented ecological phenomena that integer-order derivatives cannot capture:*

1. ***Predation pressure memory.*** *Prey exposed to intense predation maintain elevated vigilance and suppressed foraging long after the immediate threat has passed [3]. Current birth rates therefore depend on the accumulated history of predation exposure, not solely on the present predator density y(t) a carry-over effect that is structurally absent from the classical derivative.*
2. ***Resource history.*** *A population that has experienced resource limitation in recent seasons enters the present season in a physiologically weakened state, with reduced body condition and reproductive capacity. Past resource availability therefore influences present growth rates in a way that $\frac{d}{dt}$ cannot represent.*
3. ***Environmental conditioning.*** *Predators learn hunting territories and prey develop escape strategies through cumulative environmental experience. These learned behaviors are hereditary responses to past conditions rather than reactions to the instantaneous state alone.*

*The non-local kernel of the Caputo operator,*


$$\begin{array}{c} {\;}^{C}D^{\alpha }f(t)\;=\;\frac{1}{\Gamma (1-\alpha )}\int_{0}^{t}(t-s{)}^{-\alpha }\;f^{\prime }(s)\;ds, \end{array}$$

*weights all past states with a power-law decay so that more recent history carries greater influence than distant history, directly reflecting the fading nature of ecological memory. The parameter α controls the strength of this memory, with progressively longer hereditary effects as α decreases, consistent with species exhibiting slower life histories or stronger physiological carry-over [20,21].*

***Dimensional compatibility.*** *The Caputo derivative ${\;}^{C}D^{\alpha }f$ carries units of $[f]\cdot {time}^{-\alpha }$, whereas $\frac{d}{dt}f$ carries units of $[f]\cdot {time}^{-1}$. To ensure dimensional consistency, we follow the standard convention adopted in fractional biological modelling and assign all rate parameters (r, a, d, h, and related coefficients) units of ${time}^{-\alpha }$ rather than ${time}^{-1}$ [20,22]. Concretely, following Diethelm [42], each such parameter is obtained from its classical (integer-order, ${time}^{-1}$) counterpart θ by the rescaling $\theta \mapsto \theta^{\alpha }$, evaluated at the reference order α=0.95 used throughout the baseline parameter set (17); these rescaled numerical values are then held fixed while α is subsequently varied as a bifurcation parameter in Section 6, so that the biological system under study remains fixed while only the memory order of the governing operator is varied. Under this convention, every term on the right-hand side of Equations (1)–(2) carries the same units as the left-hand side, ensuring full dimensional homogeneity for all α∈(0,1]. This resolution is consistent with the broader fractional epidemic literature, where dimensional mismatch between integer- and fractional-order formulations is a recognised and solved problem. Diethelm [42] resolves an analogous mismatch in a fractional dengue model by raising each rate parameter to the power of the fractional order, while Baleanu et al. [43] resolve the mismatch in a general fractional COVID-19 model via a single auxiliary coefficient applied to the entire right-hand side. These studies confirm that the dimensional mismatch raised against fractional formulations is not a structural flaw but a standard modelling step with established solutions, of which the rescaling convention adopted here is a direct instance.*

***Cumulative histories.*** *The cumulative histories of predation pressure, resource availability, and environmental conditioning are encoded directly through the non-local convolution kernel of the Caputo operator above, rather than through any auxiliary memory variable. Because the prey and predator equations are governed by ${\;}^{C}D^{\alpha }$, the rate of change at time t is a weighted average of all past rates of change, with each of the three histories entering as follows:*

1. ***Predation pressure.*** *At each past moment s < t, the predator density y(s) suppressed prey birth through the fear term r/(1 + ky(s)). The kernel carries the imprint of those suppressed birth rates forward to the present (direct channel), while the resulting reduction in x(s) also diminishes the biomass available for predator growth at later times (indirect channel).*
2. ***Resource availability.*** *Resource limitation at past times s is reflected in a depressed growth increment* $x^{\prime }(s)$ *at that time – the physiologically weakened state described in the biological basis above. The kernel weights this past increment into the present rate of change, so a season of scarcity continues to depress current growth even after resources recover.*
3. ***Environmental conditioning.*** *Learned predator hunting strategies and prey escape behaviors are not tracked as a separate state variable; instead, their net effect on the realised interaction rate at each past time s is already embedded in* $x^{\prime }(s)$ *and* $y^{\prime }(s)$ *at that time, and is therefore carried forward by the same kernel.*

*When α=1, the Caputo derivative reduces to the classical first-order derivative and the proposed fractional-order model recovers exactly the corresponding integer-order system in Equations (1)–(2), thereby confirming both dimensional and structural consistency with the classical framework.*

The biological motivation, dimensional consistency, and interpretation of cumulative predation-pressure memory associated with the Caputo fractional derivative are discussed in Remark 1. Consequently, each term in Equations (1)–(2) possesses the same physical dimension as the Caputo derivative, ensuring dimensional homogeneity for all α∈(0,1]. The biological interpretation of each parameter is summarized in Table 2. All parameters are assumed positive unless otherwise stated, and the constraint n∈(0,1) ensures that a positive fraction of the prey population remains exposed to predation.

**Table 2:** Model parameters, their biological meanings, plausible ranges, baseline values used in numerical simulations, and representative literature supporting those values.

Symbol	Biological meaning	Plausible range	Baseline	Reference(s)
*r*	Prey intrinsic growth rate	[0.5, 4.0]	1.931873	[4,5]
*k*	Fear coefficient (prey reproduction sensitivity)	[0.1, 2.0]	0.4	[4,25]
*K*	Prey carrying capacity	[5, 50]	15.0	[7,44]
*a*	Predation attack rate	[0.5, 2.5]	1.189110	[17,24]
*b*	Holling II half-saturation coefficient	[0.1, 1.5]	0.5	[5,24]
*n*	Prey refuge fraction	[0.05, 0.70]	0.2	[5,25]
*h*	Maximum harvesting effort	[0.05, 0.9*r*]	0.318614	[7,17]
*q*	Harvest saturation coefficient	[0.05, 1.0]	0.3	[16,17]
*e*	Prey-to-predator biomass conversion efficiency	[0.1, 0.9]	0.7	[24,44]
*d*	Predator natural mortality rate	[0.1, 0.8]	0.418752	[4,7]
*m*	Predator intraspecific competition coefficient	[0.01, 0.2]	0.058079	[10,12]
*v*	Anti-predator retaliation rate	[0, 0.15]	0.046985	[10,41]
α	Caputo fractional (memory) order	(0, 1]	0.95	[7,25]

**Special-case compatibility.** The proposed model contains several previously studied systems as special cases. Setting *v* = 0 recovers the fractional fear–refuge model of Barman et al. [24]; setting *n* = 0 and *v* = 0 yields the fractional fear–harvesting model of Shi et al. [7]; and taking α=1 recovers the corresponding integer-order fear–harvesting framework studied by Umrao et al. [17]. Thus, the present formulation extends a number of existing predator–prey models within a unified fractional-order framework that simultaneously accounts for fear effects, prey refuge, anti-predator behaviour, and nonlinear harvesting.

**Parameter selection.** The model parameters and their baseline values are selected from previously published predator–prey studies and are summarized in Table 2. For each parameter, a biologically plausible range is identified from the literature, and the baseline value was chosen within that range to ensure ecological realism and consistency with earlier models. Specifically, the classical (integer-order) numerals *r* = 2.0, *K* = 15 are taken from small-mammal studies [4,5]; *k* = 0.4 within the range shown to suppress oscillations without predator extinction [4]; *a* = 1.2, *b* = 0.5 standard Holling type II values for fractional models [17,24]; *n* = 0.2 from field-observed refuge levels [5,25]; *h* = *q* = 0.3 satisfying *r* > *h* for prey persistence [7,17]; *e* = 0.7, *d* = 0.4 within terrestrial system ranges [4,44]; *v* = 0.04, *m* = 0.05 kept small to satisfy the predator-boundedness condition [10,41]. The rate-type parameters (*r*, *a*, *h*, *d*, *m*, *v*) are then rescaled via $\theta \mapsto \theta^{\alpha }$ at the reference order α=0.95 (Remark 1), giving the numerical baseline reported in Table 2; the dimensionless or population-scale parameters (*k*,*K*, *b*, *n*, *q*, *e*) are unaffected by this rescaling. At this baseline, α=0.95 lies above the Hopf threshold $\alpha^{*}\approx 0.9080$ (Section 4), placing the baseline system in the oscillatory regime [7,25].

For convenience, we restate the system (1)–(2) in compact notation for use throughout this section:


$$\begin{array}{cc} {\;}^{C}D^{\alpha }x & =\frac{r}{1+ky}\;x\left(1-\frac{x}{K}\right)-\frac{a(1-n)xy}{1+b(1-n)x}-\frac{hx}{1+qx}\equiv F_{1}(x,y), \end{array}$$ (3)

$$\begin{array}{cc} {\;}^{C}D^{\alpha }y & =\frac{ea(1-n)xy}{1+b(1-n)x}-dy-my^{2}-vxy\equiv F_{2}(x,y). \end{array}$$ (4)

with initial conditions $x(0)=x_{0}\geq 0$, $y(0)=y_{0}\geq 0$, and α∈(0,1].

**Remark 2** (Nonlinear saturation harvesting). *The term hx/(1 +* qx) is a Michaelis–*Menten (saturating) harvesting function: the catch rate rises with prey density x but approaches a finite maximum h/q rather than growing without bound, avoiding the abrupt population collapses associated with constant-yield or constant-effort harvesting* [16]*. Here h and q are independent shape parameters: h sets the harvesting intensity at low prey density, and q independently controls how quickly the catch rate saturates. Treating the two shape parameters of a saturating harvest term as independent is standard practice in this literature; for example, Hu and Cao* [45] *study an identical Michaelis–Menten harvesting term with two shape parameters (*α,γ*): their saddle–node and Hopf bifurcation results (Theorem 1,* Fig 1*; Theorem 9, Fig 6) hold* γ *fixed and vary* α *alone, while their Bogdanov–Takens bifurcation analysis (Theorem 10,* Fig 7*) explicitly treats* α *and* γ *as two independent bifurcation parameters. We follow this latter convention and vary h and q independently in our sensitivity analysis (*Table 2*,* Fig 9b*). This formulation applies to any harvesting or extraction process with finite processing capacity, not only to fisheries.*

We first state the essential lemmas that all proofs rely on.

### Preliminary lemmas

**Lemma 1. *(Generalized Mean Value Theorem*** [46]***).*** *Let* f(t)∈C[a,b] *and* ${\;}^{C}D^{\alpha }f(t)\in C(a,b)$ *with* α∈(0,1]*. Then* $f(t)=f(a)+\frac{1}{\Gamma (\alpha )}\;{\;}^{C}D^{\alpha }f(\zeta )\;(t-a{)}^{\alpha },\;a\leq \zeta \leq t.$

**Corollary 1.** *If* ${\;}^{C}D^{\alpha }f(t)\geq 0$ *for all* t∈(a,b)*, then f(t) is non-decreasing. If* ${\;}^{C}D^{\alpha }f(t)\leq 0$*, then f(t) is non-increasing.*

**Lemma 2. *(Fractional Comparison Principle*** [47]***).*** *Suppose* ${\;}^{C}D^{\alpha }u(t)\leq f(t,u(t))$ *and* ${\;}^{C}D^{\alpha }v(t)=f(t,v(t))$ *with* u(0)≤v(0)*. Then* u(t)≤v(t),for all t≥0.

**Lemma 3*. (Mittag–Leffler bound*** [47]***).*** *If* ${\;}^{C}D^{\alpha }W(t)\leq -\mu W(t)+\delta ,$ *where* μ>0 *and* δ≥0*, then* $W(t)\leq \left(W(0)-\frac{\delta }{\mu }\right)E_{\alpha }(-\mu t^{\alpha })+\frac{\delta }{\mu },$ *so that* ${lim sup}_{t\to \infty }W(t)\leq \frac{\delta }{\mu },$ *where* $E_{\alpha }(\cdot )$ *denotes the Mittag–Leffler function.*

### Non-negativity of solutions

**Theorem 1**. *All solutions (x(t), y(t)) of the system with non-negative initial conditions* x(0)≥0*,* y(0)≥0 *remain non-negative for all* t≥0*. That is, the positive orthant* ${\mathbb{R}}_{+}^{2}=\{(x,y):x\geq 0,y\geq 0\}$ *is positively invariant.*

*Proof.* Part (i) Non-negativity of *x*(*t*):

Evaluating (1) at any time *t*_1_ > 0 where *x*(*t*_1_) = 0 gives ${\;}^{C}D^{\alpha }x{|}_{x=0}=0.$ Hence x≡0 is a solu*t*ion of the prey equation when *x*(0) = 0. For *x*(0) > 0, we apply the Fractional Comparison Principle (Lemma 2): since ${\;}^{C}D^{\alpha }x\geq 0$ whenever *x* = 0 (all terms on the right-hand side vanish), the zero function v(t)≡0 satisfies ${\;}^{C}D^{\alpha }v=0\leq {\;}^{C}D^{\alpha }x$ when *x* = 0. Therefore x(t)≥v(t)=0 for all t≥0.

Part (ii) Non-negativity of *y*(*t*):

Suppose by contradiction that there exists a first time *t*_2_ > 0 such that *y*(*t*) > 0 for $t\in [0,t_{2})$, *y*(*t*_2_)=0, and *y*(*t*) < 0 for *t* > *t*_2_. Evaluating the second equation at *t* = *t*_2_ with *y*(*t*_2_)=0, we obtain


$$\begin{array}{c} {\;}^{C}D^{\alpha }y{|}_{t=t_{2}}=\frac{ea(1-n)x(t_{2})\cdot 0}{1+b(1-n)x(t_{2})}-d\cdot 0-m\cdot 0^{2}-v\cdot x(t_{2})\cdot 0=0. \end{array}$$

By Lemma 1 similarly, we obtain $y(t_{2}^{+})=0,$ which contradicts *y*(*t*) < 0 for *t* > *t*_2_. Therefore, y(t)≥0 for all t≥0. Hence all solutions remain in ${\mathbb{R}}_{+}^{2}$ for all t≥0. □

### Boundedness of solutions

**Theorem 2**. *All non-negative solutions (x(t),y(t)) of the system are uniformly bounded and remain in the region:* $\Omega =\{(x,y)\in {\mathbb{R}}_{+}^{2}:0\leq x(t)\leq \frac{K(r+\mu {)}^{2}}{4r\mu }+\varepsilon ,\;0\leq y(t)\leq \frac{eK(r+\mu {)}^{2}}{4r\mu }+\varepsilon ,\;\text{for any }\varepsilon >0\}$ *where* μ∈(0,d) *is any sufficiently small positive constant,* e∈(0,1) *is the biomass conversion efficiency, and* $\delta =K(r+\mu {)}^{2}/(4r)$ *is defined in the proof below.*

*Proof.* Define $W(t)=x(t)+\frac{1}{e}y(t)$. Since $\frac{1}{1+ky}\leq 1$, the prey equation gives ${\;}^{C}D^{\alpha }x\leq rx(1-x/K)$, so by the comparison principle ${lim sup}_{t\to \infty }x(t)\leq K$. Computing ${\;}^{C}D^{\alpha }W$ and substituting the model equations, the predation terms cancel and we obtain


$$\begin{array}{c} {\;}^{C}D^{\alpha }W\leq rx\;\left(1-\frac{x}{K}\right)-\frac{dy}{e}. \end{array}$$

For any μ∈(0,d), adding and subtracting μW and completing the square in *x* yields


$$\begin{array}{c} {\;}^{C}D^{\alpha }W\leq \delta -\mu W,\;\delta =\frac{K(r+\mu {)}^{2}}{4r}. \end{array}$$

Lemma 3 then gives ${lim sup}_{t\to \infty }W(t)\leq \delta /\mu =K(r+\mu {)}^{2}/(4r\mu )$, from which the stated bounds on *x*(*t*) and *y*(*t*) follow immediately for any ε>0.

### Existence and uniqueness of solutions

**Theorem 3**. *For any initial condition* $(x_{0},y_{0})\in {\mathbb{R}}_{+}^{2}$*, the system possesses a unique solution (x(t), y(t)) on* [0,∞)*.*

*Proof.* Define $X=(x,y{)}^{T}$ and $F=(F_{1},F_{2}{)}^{T}$. From Theorem 2, all solutions remain in the compact set Ω, so ‖X‖≤M for some finite *M*. Using the bounds $\frac{1}{1+ky}\leq 1$, $\frac{1}{1+b(1-n)x}\leq 1$, $\frac{1}{1+qx}\leq 1$, and the mean-value estimate for *hx*/(1 + *qx*), straightforward calculation gives


$$\begin{array}{c} |F_{1}(X_{1})-F_{1}(X_{2})|\leq L_{1}|x_{1}-x_{2}|+L_{2}|y_{1}-y_{2}|, \end{array}$$

with $L_{1}=r+2rM/K+a(1-n)M+h$ and $L_{2}=rkM+a(1-n)M$, and similarly for *F*_2_. Hence $\left\|F(X_{1})-F(X_{2})\|\leq ℒ\|X_{1}-X_{2}\right\|$ with ℒ<∞. Global existence and uniqueness then follow from the standard Caputo FDE theorem [20,44].

## 3. Equilibrium and stability analysis

To determine the equilibrium points of the system, we set ${\;}^{C}D^{\alpha }x=0,\;{\;}^{C}D^{\alpha }y=0.$

### Trivial equilibrium *E*_0_

Setting *x* = 0 and *y* = 0, the system is satisfied. Hence, the trivial equilibrium is given by *E*_0_ = (0,0). The equilibrium *E*_0_ represents total extinction of both prey and predator populations. This equilibrium always exists unconditionally.

### Prey-only equilibrium *E*_1_

Setting *y* = 0, we obtain $E_{1}=(x_{1}^{*},0)$, where


$$\begin{array}{c} x_{1}^{*}=\frac{\left(K-\frac{1}{q}\right)+\sqrt{{\left(K-\frac{1}{q}\right)}^{2}-\frac{4K(h-r)}{rq}}}{2}. \end{array}$$

The existence Conditions for *E*_1_ are given by


$$\begin{array}{c} (C1\text{-}E_{1})\;r>h\;\text{(prey growth must exceed harvesting pressure)}, \end{array}$$

$$\begin{array}{c} (C2\text{-}E_{1})\;{\left(K-\frac{1}{q}\right)}^{2}>\frac{4K(h-r)}{rq}\;\text{(discriminant non-negative for }E_{1}\text{)}. \end{array}$$

When *y* = 0, there is no predator, hence the refuge term (1−n) disappears. Therefore, the prey-only equilibrium *E*_1_ is independent of the refuge parameter *n* and the anti-predator parameter *v*, which is biologically consistent.

### Coexistence equilibrium *E*_2_

Let $x^{*}\neq 0$, $y^{*}\neq 0$. The coexistence equilibrium $E_{2}=(x^{*},y^{*})$ is obtained by solving the system.

From the predator equation, we obtain $\frac{ea(1-n)x^{*}}{1+b(1-n)x^{*}}=d+my^{*}+vx^{*}.$

This leads to the quadratic equation in $x^{*}$: $bv(1-n)x^{*2}-(\Pi_{0}-Q)x^{*}+R=0,$ where


$$\begin{array}{c} \Pi_{0}=(1-n)(ea-bd),\;Q=v+bm(1-n)y^{*},\;R=d+my^{*}. \end{array}$$

Thus, $x^{*}=\frac{(\Pi_{0}-Q)\pm \sqrt{(\Pi_{0}-Q{)}^{2}-4bv(1-n)R}}{2bv(1-n)}$.

Substituting the quadratic expression for $x^{*}(y^{*})$ into the prey equation


$$\begin{array}{c} \frac{r}{1+ky^{*}}\;\left(1-\frac{x^{*}}{K}\right)=\frac{a(1-n)y^{*}}{1+b(1-n)x^{*}}+\frac{h}{1+qx^{*}} \end{array}$$

Clearing all denominators yields the following polynomial in $y^{*}$:


$$\begin{array}{c} B_{4}y^{*4}+B_{3}y^{*3}+B_{2}y^{*2}+B_{1}y^{*}+B_{0}=0, \end{array}$$

where


$$\begin{array}{cc} B_{4} & =Kmkbv(1-n)q,\\ B_{3} & =K\left[mk(1+qx_{0})+(dk+m)bv(1-n)q+\frac{mk\;\Pi_{0}}{2bv(1-n)}\right]-hKk\cdot bv(1-n),\\ B_{2} & =K\left[(dk+m)(1+qx_{0})+mkx_{0}q+dbv(1-n)q\right]-er(Kq-1)x_{0}-hKk,\\ B_{1} & =K\left[d(1+qx_{0})+(dk+m)x_{0}+vx_{0}(1+kx_{0}q)\right]-erK(1-qx_{0})-hK,\\ B_{0} & =erK-Kdx_{0}-hK, \end{array}$$

where $x_{0}=\frac{\Pi_{0}-v}{2bv(1-n)}$. The positive coexistence equilibria correspond to the positive real roots of this polynomial. Instead of solving the quartic explicitly, we apply Descartes’ Rule of Signs to the coefficient sequence $\{B_{4},B_{3},B_{2},B_{1},B_{0}\}.$ According to Descartes’ Rule, the number of positive real roots is equal to the number of sign changes in the coefficient sequence or less than it by an even integer. Therefore, the sign pattern of the coefficients provides information on the possible number of positive coexistence equilibria without computing the roots explicitly. The admissible cases are summarized in Table 3. Hence, Descartes’ Rule serves as a convenient criterion for determining the existence and multiplicity of biologically feasible coexistence equilibria.

**Table 3:** Positive root analysis using Descartes’ rule of signs.

Case	Sign pattern $\left\{B_{4},B_{3},B_{2},B_{1},B_{0}\right\}$	Sign changes	Positive roots
Case 1	(+,+,+,+,−)	1	Exactly 1
Case 2	(+,+,−,+,+)	2	0 or 2
Case 3	(+,−,+,−,+)	4	0, 2 or 4

**Remark 3** *(Case v = 0)***.** *When v* = *0 (no anti-predator behavior), the expression for x*_*0*_ *above involves division by zero and is undefined. In this limit the quadratic for* $x^{*}$ *degenerates: the predator isocline* $\frac{ea(1-n)x^{*}}{1+b(1-n)x^{*}}=d+my^{*}$ *gives* $x^{*}$ *explicitly as a function of* $y^{*}$ *via*


$$\begin{array}{c} x^{*}=\frac{d+my^{*}}{(ea-bd)(1-n)-bmy^{*}}, \end{array}$$

*provided* $(ea-bd)(1-n)>bmy^{*}$, *and substituting into the prey nullcline yields a cubic (rather than quartic) polynomial in* $y^{*}$. *The analysis throughout this section therefore assumes v > 0; setting v = 0 recovers the simpler equilibrium structure of Barman et al.* [24].

The existence Conditions for *E*_2_ are given by


$$\begin{array}{c} (C2)\;ea(1-n)>bd(1-n)+v. \end{array}$$

$$\begin{array}{c} (C3)\;x_{1}^{*}<K\;\text{(prey-only equilibrium lies below carrying capacity).} \end{array}$$

The coexistence equilibrium exists if the polynomial admits at least one positive real root $y^{*}>0$.

**Remark 4** *(Numerical Verification of Coexistence Equilibrium E*_*2*_*). For the baseline parameter set (17), we numerically verify the existence of the coexistence equilibrium E*_*2*_*. Substituting the baseline values into the coefficient expressions, we obtain the sign pattern* $\{B_{4},\;B_{3},\;B_{2},\;B_{1},\;B_{0}\}\;=\;\{+,\;+,\;+,\;+,\;-\},$ *which corresponds to Case 1 of* Table 3 *(exactly one sign change), guaranteeing a unique positive root* $y^{*}>0$ *by Descartes’ Rule of Signs. Solving the quartic numerically yields* $y^{*}\approx 1.4215,$ *and substituting back into the quadratic expression for* $x^{*}$ *gives* $x^{*}\approx 1.2707.$ *Thus the coexistence equilibrium is* $E_{2}\approx (1.2707,\;1.4215)$*, which is biologically feasible since* $x^{*}<K=15$ *and* $y^{*}>0$*, and the conditions (C1)–(C3) of Theorem 4 are all satisfied at the new baseline parameter values. This unique-equilibrium structure was cross-checked by an independent exhaustive two-dimensional search of the nullcline system over* $\left(x_{0},y_{0})\in [0.05,14.5]\times [0.05,6\right]$*, which located no second positive root, confirming Case 1 numerically as well as analytically. Note that* $x^{*}$ *and* $y^{*}$ *have both increased relative to the previous baseline (*1.108→1.2707*,* 1.432→1.4215*), reflecting the higher rescaled attack rate a and mortality d at* α=0.95.

**Remark 5** (Critical Thresholds for Predator Extinction). *Condition (C2), namely* ea(1−n)>bd(1−n)+v*, can be rewritten as* (ea−bd)(1−n)>v*, which defines two sharp ecological boundaries:*

(i) *Critical refuge level. Solving* $(ea-bd)(1-n^{*})=v$ *for* $n^{*}$ *gives* $n^{*}=1-\frac{v}{ea-bd},\text{provided\textasciitilde{}}ea>bd,$

*such that for* $n>n^{*}$ *the coexistence equilibrium E*_*2*_ *ceases to exist and the predator faces unavoidable extinction regardless of all other parameters. At the baseline values (e = 0.7, a = 1.189110, b = 0.5, d = 0.418752, v = 0.046985),*


$$\begin{array}{c} ea-bd=0.7\times 1.189110-0.5\times 0.418752=0.83238-0.20938=0.62300,\;n^{*}=1-\frac{0.046985}{0.62300}=0.9246. \end{array}$$

*Managing refuge so that n < 0.9246 is therefore necessary for predator persistence at the new baseline parameter set (only marginally tighter than the previous bound of 0.9375).*

(ii) *Critical anti-predator strength. Setting n at its baseline value and solving for* $v^{*}$ *gives* $v^{*}=(1-n)(ea-bd)\equiv \Pi_{0}.$ *At baseline (n = 0.2),* $v^{*}=0.8\times 0.62300=0.4984.$ *Above* $v^{*}$, *the energetic cost imposed by prey retaliation exceeds the predator’s net gain from predation, again driving E*_*2*_ *out of existence. Conservation programs that create dense prey aggregations may inadvertently push v beyond this threshold, triggering predator collapse. Both extinction boundaries move only slightly relative to the previous parameter set (from* $n^{*}\approx 0.9375$ *to 0.9246, and from* $v^{*}\approx 0.512$ *to 0.4984), because the higher rescaled attack rate a is nearly offset by the higher rescaled mortality d at* α=0.95.

(iii) *Note on Fig 6. The extinction boundary plotted in Fig 6 is the curve* ea(1−n)=bd(1−n)+v, *i.e.,* (ea−bd)(1−n)=v, *which is the correct condition (C2). The dashed reference lines in that figure are drawn at the analytically correct values* $n^{*}\approx 0.9246$ *and* $v^{*}\approx 0.4984$ computed above, corresponding to condition (C2) *evaluated at the baseline parameters.*

**Theorem 4.** *The coexistence equilibrium* $E_{2}=(x^{*},y^{*})$ *of the system exists if and only if the polynomial*


$$\begin{array}{c} B_{4}y^{*4}+B_{3}y^{*3}+B_{2}y^{*2}+B_{1}y^{*}+B_{0}=0 \end{array}$$

*admits at least one positive real root. A sufficient condition for a unique positive root is*


$$\begin{array}{c} B_{0}<0\;\text{and}\;B_{4}>0. \end{array}$$

*Under this condition, E*_*2*_ *is biologically feasible provided that*


$$\begin{array}{c} (C1)\;r>h,\;(C2)\;ea(1-n)>bd(1-n)+v,\;(C3)\;x^{*}<K. \end{array}$$

**Remark 6**. *When multiple positive roots exist (Cases 2 and 3), the model exhibits bistability or multistability: the system may converge to different coexistence states depending on initial conditions.*

***Numerical example for Case 2 (bistability).*** *With the modified parameter set* r=2.0,k=0.05,K=15,a=1.8,b=0.8,n=0.1,h=0.3,q=0.3,e=0.7,d=0.35,m=0.02,v=0.01,α=0.90*, the quartic coefficient vector is* $\left\{B_{4},B_{3},B_{2},B_{1},B_{0}\}=\{+,+,-,+,+\right\}$ *(two sign changes, Case 2), and numerical root-finding yields two positive roots* $y_{1}^{*}\approx 0.78$ *and $y_{2}^{*}\approx 2.15$ with corresponding* $x_{1}^{*}\approx 1.62$ *and* $x_{2}^{*}\approx 0.87$*. Trajectories starting near* $\left(x_{0},y_{0})=(0.5,0.3\right)$ *converge to the lower coexistence state* $\left(x_{1}^{*},y_{1}^{*}\right)$*, while those starting near (3.0,3.0) converge to* $\left(x_{2}^{*},y_{2}^{*}\right)$*, confirming bistability. Constructing full basin-of-attraction diagrams for the bistable regime is left as future work.*

## Local stability analysis

### Key stability theorem for fractional-order systems

**Theorem 5*. (Matignon,*** [48]***).*** *Consider the fractional-order autonomous system* ${\;}^{C}D^{\alpha }X=F(X),\;\alpha \in (0,1].$ *Let* $E^{*}=(x^{*},y^{*})$ *be an equilibrium point and* $J(E^{*})$ *be the Jacobian matrix evaluated at* $E^{*}$. Then $E^{*}$ *is locally asymptotically stable if and only if every eigenvalue* $\lambda_{i}$ *of* $J(E^{*})$ *satisfies:* $|\text{arg}(\lambda_{i})|>\frac{\alpha \pi }{2}.$

*That is, all eigenvalues must lie outside the closed angular sector* $|\text{arg}(\lambda )|\leq \frac{\alpha \pi }{2}$ *in the complex plane.*

### The Jacobian matrix of the system

For our model, the Jacobian matrix at any point (*x*,*y*) is:


$$\begin{array}{c} J(x,y)=(\begin{array}{cc} J_{11} & J_{12}\\ J_{21} & J_{22} \end{array}) \end{array}$$

where:


$$\begin{array}{cc} J_{11} & =\frac{\partial F_{1}}{\partial x}=\frac{r}{1+ky}\left(1-\frac{2x}{K}\right)-\frac{a(1-n)y}{{\left[1+b(1-n)x\right]}^{2}}-\frac{h}{(1+qx{)}^{2}},\\ J_{12} & =\frac{\partial F_{1}}{\partial y}=-\frac{rkx\left(1-\frac{x}{K}\right)}{(1+ky{)}^{2}}-\frac{a(1-n)x}{1+b(1-n)x},\\ J_{21} & =\frac{\partial F_{2}}{\partial x}=\frac{ea(1-n)y}{{\left[1+b(1-n)x\right]}^{2}}-vy,\\ J_{22} & =\frac{\partial F_{2}}{\partial y}=\frac{ea(1-n)x}{1+b(1-n)x}-d-2my-vx. \end{array}$$

### Local stability at *E*_0_ = (0,0)

**Theorem 6**. *The trivial equilibrium E*_*0*_ *= (0,0) is always unstable.*

*Proof.* Evaluating the Jacobian at *E*_0_ = (0,0): $J(E_{0})=(\begin{array}{cc} r-h & 0\\ 0 & -d \end{array}).$

The eigenvalues are: $\lambda_{1}=r-h,\;\lambda_{2}=-d.$ Since *d* > 0, we have $\lambda_{2}=-d<0$, so $|\text{arg}(\lambda_{2})|=\pi >\frac{\alpha \pi }{2}.$ For $\lambda_{1}=r-h$, under the biologically relevant condition *r* > *h*, we have $\lambda_{1}>0$, hence $|\text{arg}(\lambda_{1})|=0<\frac{\alpha \pi }{2}.$ Thus, Matignon’s stability condition is violated. Therefore, *E*_0_ is unstable. □

**Remark 7 *(Biological interpretation of E***_***0***_***).*** *The instability of the extinction equilibrium E*_*0*_*=(0,0) indicates that the ecosystem cannot remain in a state where both species are absent whenever condition (C1), (r > h), holds. Ecologically, this means that the prey population possesses sufficient reproductive capacity to recover from very low densities despite harvesting pressure. Moreover, since the predator eigenvalue is* $\left(\lambda_{2}=-d<0\right)$*, predators cannot persist in the absence of prey and inevitably decline to extinction. Thus, E*_*0*_ *is biologically unrealistic as a long-term outcome under condition (C1).*

### Local stability at $E_{1}=(x_{1}^{*},0)$

**Theorem 7**. *The prey-only equilibrium* $E_{1}=(x_{1}^{*},0)$ *is locally asymptotically stable if and only if* $ea(1-n)x_{1}^{*}<d\left(1+b(1-n)x_{1}^{*}\right)+vx_{1}^{*}\left(1+b(1-n)x_{1}^{*}\right),$ *equivalently,* $\frac{ea(1-n)x_{1}^{*}}{1+b(1-n)x_{1}^{*}}<d+vx_{1}^{*}\;(C5).$

*Proof.* Evaluating the Jacobian at $E_{1}=(x_{1}^{*},0)$: $J(E_{1})=(\begin{array}{cc} J_{11}^{\left(1\right)} & J_{12}^{\left(1\right)}\\ 0 & J_{22}^{\left(1\right)} \end{array})$ where $J_{11}^{\left(1\right)}=r\left(1-\frac{2x_{1}^{*}}{K}\right)-\frac{h}{(1+qx_{1}^{*}{)}^{2}},J_{12}^{\left(1\right)}=-rkx_{1}^{*}\left(1-\frac{x_{1}^{*}}{K}\right)-\frac{a(1-n)x_{1}^{*}}{1+b(1-n)x_{1}^{*}},J_{22}^{\left(1\right)}=\frac{ea(1-n)x_{1}^{*}}{1+b(1-n)x_{1}^{*}}-d-vx_{1}^{*}.$

Since *J*(*E*_1_) is upper triangular, the eigenvalues are: $\lambda_{1}=r\left(1-\frac{2x_{1}^{*}}{K}\right)-\frac{h}{(1+qx_{1}^{*}{)}^{2}},$ $\lambda_{2}=\frac{ea(1-n)x_{1}^{*}}{1+b(1-n)x_{1}^{*}}-d-vx_{1}^{*}.$

From the prey-only equilibrium condition, $r\left(1-\frac{x_{1}^{*}}{K}\right)=\frac{h}{1+qx_{1}^{*}}.$ It follows that $r\left(1-\frac{2x_{1}^{*}}{K}\right)-\frac{h}{(1+qx_{1}^{*}{)}^{2}}<0,$ hence $\lambda_{1}<0$, implying $|\text{arg}(\lambda_{1})|=\pi >\frac{\alpha \pi }{2}.$ The stability depends on $\lambda_{2}<0$, which gives condition (C5): $\;\frac{ea(1-n)x_{1}^{*}}{1+b(1-n)x_{1}^{*}}<d+vx_{1}^{*}.$ If (C5) holds, then both eigenvalues satisfy Matignon’s condition, and *E*_1_ is locally asymptotically stable. If (C5) is violated, then $\lambda_{2}>0$ and *E*_1_ is unstable. □

**Remark 8** (Biological interpretation of E_1_). *The prey-only equilibrium* $E_{1}=(x_{1}^{*},0)$ *represents a situation in which the prey population persists while the predator becomes extinct. Condition (C5) implies that the predator’s net gain from predation is insufficient to overcome its mortality and the costs associated with prey retaliation. Ecologically, this suggests that strong prey refuge, effective anti-predator behaviour, or low predation efficiency can prevent predator establishment even when prey are abundant. Consequently, E*_*1*_ *corresponds to a predator-exclusion state in which the prey population survives in the absence of sustained predation pressure.*

### Local stability at $E_{2}=(x^{*},y^{*})$

**Theorem 8**. *The coexistence equilibrium* $E_{2}=(x^{*},y^{*})$ *is locally asymptotically stable if and only if*


$$\begin{array}{c} (S1)\;\mathrm{tr}(J(E_{2}))=J_{11}^{\left(2\right)}+J_{22}^{\left(2\right)}<0, \end{array}$$

$$\begin{array}{c} (S2)\;\det(J(E_{2}))=J_{11}^{\left(2\right)}J_{22}^{\left(2\right)}-J_{12}^{\left(2\right)}J_{21}^{\left(2\right)}>0. \end{array}$$

*Proof.* Evaluating the Jacobian at $E_{2}=(x^{*},y^{*})$ gives:


$$\begin{array}{cc} J_{11} & =\frac{\partial F_{1}}{\partial x}=\frac{r}{1+ky}\left(1-\frac{2x}{K}\right)-\frac{a(1-n)y}{{\left[1+b(1-n)x\right]}^{2}}-\frac{h}{(1+qx{)}^{2}},\\ J_{12} & =\frac{\partial F_{1}}{\partial y}=-\frac{rkx\left(1-\frac{x}{K}\right)}{(1+ky{)}^{2}}-\frac{a(1-n)x}{1+b(1-n)x},\\ J_{21} & =\frac{\partial F_{2}}{\partial x}=\frac{ea(1-n)y}{{\left[1+b(1-n)x\right]}^{2}}-vy,\\ J_{22} & =\frac{\partial F_{2}}{\partial y}=\frac{ea(1-n)x}{1+b(1-n)x}-d-2my-vx. \end{array}$$

From the predator equilibrium condition, $\frac{ea(1-n)x^{*}}{1+b(1-n)x^{*}}=d+my^{*}+vx^{*},$ hence $J_{22}^{\left(2\right)}=-my^{*}<0.$ The characteristic equation is $\lambda^{2}-\mathrm{tr}(J)\lambda +\det(J)=0,$ or equivalently, $\lambda^{2}+p_{c}\lambda +q_{c}=0,\;\text{where }p_{c}=-\mathrm{tr}(J(E_{2})),\;q_{c}=\det(J(E_{2})).$ By the fractional Routh–Hurwitz conditions [49], *E*_2_ is locally asymptotically stable if and only if p>0andq>0, which gives conditions (S1) and (S2). Since $J_{22}^{\left(2\right)}=-my^{*}<0$, condition (S1) simplifies to $J_{11}^{\left(2\right)}<my^{*}.$

**Remark 9** (Biological interpretation of coexistence stability). *Conditions (S1) and (S2) ensure that the coexistence equilibrium* $E_{2}=(x^{*},y^{*})$ *is locally asymptotically stable. Ecologically, these conditions imply a balance between prey growth, predation, harvesting, and predator mortality, allowing both species to persist at stable population levels. The stabilising effects of fear, prey refuge, and anti-predator behaviour reduce excessive predator–prey interactions and help suppress large population fluctuations. Consequently, when (S1) and (S2) hold, small perturbations from the coexistence state decay over time and the ecosystem returns to a stable predator–prey balance.*

## Global stability analysis

### Preliminary lemmas for fractional global stability

**Lemma 4.** *(Aguila-Camacho, Duarte-Mermoud and Gallegos,* [50]*). Let* x(t)∈ℝ *be a continuous and differentiable function. Then for any* t≥0 *and* α∈(0,1]*:*


$$\begin{array}{c} \frac{1}{2}\;{\;}^{C}D^{\alpha }[x(t){]}^{2}\leq x(t)\;{\;}^{C}D^{\alpha }x(t). \end{array}$$

**Lemma 5.** *(Vargas-De-León,* [51] *Volterra-type inequality). Let x(t) > 0 be a continuously differentiable function and* $x^{*}>0$ *a constant. Then for* α∈(0,1]*:*


$$\begin{array}{c} {\;}^{C}D^{\alpha }\left(x-x^{*}-x^{*}\ln\frac{x}{x^{*}}\right)\leq \left(1-\frac{x^{*}}{x}\right)\;{\;}^{C}D^{\alpha }x. \end{array}$$

**Lemma 6.** *(Generalized LaSalle Invariance Principle for Fractional Systems, Huo et al.,* [52*]). Let* $E^{*}$ *be an equilibrium of* ${\;}^{C}D^{\alpha }X=F(X)$*,* α∈(0,1]*. Let V(X) be a continuously differentiable function satisfying:*

(i) V(X)≥0 *with equality only at* $E^{*}$,
(ii) ${\;}^{C}D^{\alpha }V(X)\leq 0$ *along all trajectories.*

*Then* $E^{*}$ *is globally asymptotically stable.*

### Global stability of $E_{1}=(x_{1}^{*},0)$

**Theorem 9**. *Under condition (C5),*


$$\begin{array}{c} \frac{ea(1-n)x_{1}^{*}}{1+b(1-n)x_{1}^{*}}<d+vx_{1}^{*}, \end{array}$$

*the prey-only equilibrium* $E_{1}=(x_{1}^{*},0)$ *is globally asymptotically stable in the region*


$$\begin{array}{c} \Omega_{1}=\{(x,y)\in {\mathbb{R}}_{+}^{2}:y=0\}. \end{array}$$

*Proof.* Define the Volterra-type Lyapunov function:


$$\begin{array}{c} V_{1}(x,y)=\left(x-x_{1}^{*}-x_{1}^{*}\ln\frac{x}{x_{1}^{*}}\right)+c_{1}y, \end{array}$$

where *c*_1_ > 0 is a positive constant to be determined, and $x_{1}^{*}$ is the prey-only equilibrium.

Clearly, $V_{1}(x,y)\geq 0$, with equality only at $\left(x,y)=(x_{1}^{*},0\right)$.

Using Lemma 5, we obtain:


$$\begin{array}{c} {\;}^{C}D^{\alpha }V_{1}\leq \left(1-\frac{x_{1}^{*}}{x}\right){\;}^{C}D^{\alpha }x+c_{1}{\;}^{C}D^{\alpha }y. \end{array}$$

Substituting the model equations:


$$\begin{array}{c} {\;}^{C}D^{\alpha }V_{1}\leq \left(1-\frac{x_{1}^{*}}{x}\right)\left[\frac{r}{1+ky}x\left(1-\frac{x}{K}\right)-\frac{a(1-n)xy}{1+b(1-n)x}-\frac{hx}{1+qx}\right] \end{array}$$

$$\begin{array}{c} +c_{1}\left[\frac{ea(1-n)xy}{1+b(1-n)x}-dy-my^{2}-vxy\right]. \end{array}$$

At *E*_1_, the equilibrium condition gives:


$$\begin{array}{c} r\left(1-\frac{x_{1}^{*}}{K}\right)=\frac{h}{1+qx_{1}^{*}}. \end{array}$$

Using this, one obtains:


$$\begin{array}{c} \left(1-\frac{x_{1}^{*}}{x}\right)\left[rx\left(1-\frac{x}{K}\right)-\frac{hx}{1+qx}\right]\leq -\frac{r}{Kx}(x-x_{1}^{*}{)}^{2}\leq 0. \end{array}$$

Thus,


$$\begin{array}{c} {\;}^{C}D^{\alpha }V_{1}\leq -\frac{r}{Kx}(x-x_{1}^{*}{)}^{2}-\frac{x_{1}^{*}a(1-n)y}{1+b(1-n)x} \end{array}$$

$$\begin{array}{c} +c_{1}y\left[\frac{ea(1-n)x}{1+b(1-n)x}-d-my-vx\right]. \end{array}$$

At $x=x_{1}^{*}$, the *y*-term becomes:


$$\begin{array}{c} \frac{ea(1-n)x_{1}^{*}}{1+b(1-n)x_{1}^{*}}-d-vx_{1}^{*}. \end{array}$$

Under condition (C5), this is negative. Hence choose:


$$\begin{array}{c} c_{1}=\frac{x_{1}^{*}a(1-n)/(1+b(1-n)x_{1}^{*})}{d+vx_{1}^{*}-\frac{ea(1-n)x_{1}^{*}}{1+b(1-n)x_{1}^{*}}}>0. \end{array}$$

Then all terms become non-positive, and we obtain:


$$\begin{array}{c} {\;}^{C}D^{\alpha }V_{1}\leq -\frac{r}{Kx}(x-x_{1}^{*}{)}^{2}-c_{1}my^{2}-\phi (x,y)\leq 0, \end{array}$$

where ϕ(x,y)≥0, with equality only at $\left(x,y)=(x_{1}^{*},0\right)$.

By the generalized LaSalle invariance principle (Lemma 6), the equilibrium $E_{1}=(x_{1}^{*},0)$ is globally asymptotically stable.□

### Global stability of $E_{2}=(x^{*},y^{*})$

**Theorem 10** (Sufficient conditions for global stability of E_2_). *Suppose (S1) and (S2) hold (local asymptotic stability of E*_*2*_*), and suppose additionally that the following sufficient (but not necessary) Lyapunov conditions are satisfied:*


$$\begin{array}{c} \text{(G1)}:\;\frac{r}{K}>\frac{a(1-n)y^{*}}{{\left[1+b(1-n)x^{*}\right]}^{2}}+\frac{hq}{{\left(1+qx^{*}\right)}^{2}}, \end{array}$$

$$\begin{array}{c} \text{(G2)}:\;{\left[\frac{rkx^{*}}{(1+ky^{*}{)}^{2}}+\frac{a(1-n)x^{*}}{1+b(1-n)x^{*}}\right]}^{2}<4\cdot \frac{r}{K}\left[\frac{ea(1-n)x^{*}}{{\left[1+b(1-n)x^{*}\right]}^{2}}-v\right]y^{*}m. \end{array}$$

*Then* $E_{2}=(x^{*},y^{*})$ *is globally asymptotically stable in* Ω*. Conditions (G1)–(G2) are conservative: they are satisfied in parameter regimes with strong prey self-regulation relative to predation intensity (large r/K, small n and a), but fail at the baseline parameter set (17). In that regime, global stability is supported by numerical evidence from 240 trajectories rather than by this theorem.*

*Proof.* We construct the Volterra-type Lyapunov function:


$$\begin{array}{c} V_{2}(x,y)=\left(x-x^{*}-x^{*}\ln\frac{x}{x^{*}}\right)+\frac{1}{e}\left(y-y^{*}-y^{*}\ln\frac{y}{y^{*}}\right). \end{array}$$

This function satisfies:

- $V_{2}(x,y)\geq 0$ for all $(x,y)\in {\mathbb{R}}_{+}^{2}$,
- $V_{2}(x^{*},y^{*})=0$,

- $V_{2}\to +\infty$ as $x\to 0^{+}$, x→∞, $y\to 0^{+}$, or y→∞.

Applying Lemma 5, we get


$$\begin{array}{c} {\;}^{C}D^{\alpha }V_{2}\leq \left(1-\frac{x^{*}}{x}\right){\;}^{C}D^{\alpha }x+\frac{1}{e}\left(1-\frac{y^{*}}{y}\right){\;}^{C}D^{\alpha }y. \end{array}$$

Substituting in system equations:


$$\begin{array}{c} {\;}^{C}D^{\alpha }V_{2}\leq \left(1-\frac{x^{*}}{x}\right)\left[\frac{r}{1+ky}x\left(1-\frac{x}{K}\right)-\frac{a(1-n)xy}{1+b(1-n)x}-\frac{hx}{1+qx}\right] \end{array}$$

$$\begin{array}{c} +\frac{1}{e}\left(1-\frac{y^{*}}{y}\right)\left[\frac{ea(1-n)xy}{1+b(1-n)x}-dy-my^{2}-vxy\right]. \end{array}$$

Using the equilibrium conditions at $E_{2}=(x^{*},y^{*})$:


$$\begin{array}{c} \frac{r}{1+ky^{*}}x^{*}\left(1-\frac{x^{*}}{K}\right)=\frac{a(1-n)x^{*}y^{*}}{1+b(1-n)x^{*}}+\frac{hx^{*}}{1+qx^{*}}, \end{array}$$ (I)

$$\begin{array}{c} \frac{ea(1-n)x^{*}y^{*}}{1+b(1-n)x^{*}}=dy^{*}+m(y^{*}{)}^{2}+vx^{*}y^{*}. \end{array}$$ (II)

Let $X=x-x^{*}$ and $Y=y-y^{*}$. After simplification, we obtain:


$$\begin{array}{c} {\;}^{C}D^{\alpha }V_{2}\leq -P_{11}(x-x^{*}{)}^{2}-P_{22}(y-y^{*}{)}^{2}+2P_{12}(x-x^{*})(y-y^{*}), \end{array}$$

where:


$$\begin{array}{c} P_{11}=\frac{r}{K}-\frac{a(1-n)y^{*}}{{\left[1+b(1-n)x^{*}\right]}^{2}}-\frac{hq}{(1+qx^{*}{)}^{2}},\;P_{22}=\frac{m}{e}y^{*},\;P_{12}=\frac{1}{2}\left[\frac{rkx^{*}}{(1+ky^{*}{)}^{2}}+\frac{a(1-n)x^{*}}{1+b(1-n)x^{*}}+\frac{v}{e}y^{*}\right]. \end{array}$$

The quadratic form is negative definite if:


$$\begin{array}{c} M=(\begin{array}{cc} P_{11} & -P_{12}\\ -P_{12} & P_{22} \end{array}) \end{array}$$

is positive definite. By Sylvester’s criterion:


$$\begin{array}{c} P_{11}>0\;(\text{G1}),\;P_{11}P_{22}-P_{12}^{2}>0\;(\text{G2}). \end{array}$$

Since $P_{22}=\frac{m}{e}y^{*}>0$, condition (G1) is essential. Under (G1) and (G2),


$$\begin{array}{c} {\;}^{C}D^{\alpha }V_{2}\leq 0, \end{array}$$

with equality only at $\left(x,y)=(x^{*},y^{*}\right)$. Hence, by the generalized LaSalle invariance principle, every trajectory in Ω converges to *E*_2_. Therefore, *E*_2_ is globally asymptotically stable in Ω. □

**Remark 10. *(Scope of Theorem 10 and numerical evidence at the baseline)*** *Conditions (G1)–(G2) are sufficient but not necessary for global asymptotic stability; they are conservative, requiring prey self-regulation r/K to dominate the combined curvature of predation, fear, and harvesting at equilibrium. At the baseline parameters (17) with* $E_{2}\approx (1.2707,\;1.4215)$*, condition (G1) fails:* r/K≈0.129 *against a right-hand side of larger magnitude. Thus Theorem 10 gives no Lyapunov-based guarantee at the baseline and should be read as a theoretical result valid for strongly self-regulated regimes (large r, small K, low attack rate a, mild harvesting curvature q) rather than a claim about the empirically common moderate-exploitation scenario used in our simulations. Because the analytic conditions fail at the baseline, we rely on extensive numerical evidence instead: simulations from 240 initial conditions uniformly covering* [0.1,12]×[0.05,6] (Fig 18a, α=0.80*) show every trajectory converging to E*_*2*_*, strongly supporting global stability at the baseline even though the Lyapunov argument does not formally certify it.*

## 4. Hopf Bifurcation analysis

### Key theorem for fractional-order Hopf Bifurcation

**Theorem 11** (Li and Wu [28]). *Consider the fractional-order autonomous system* ${\;}^{C}D^{\alpha }X=F(X)$*,* α∈(0,1]*. Let* $E^{*}=(x^{*},y^{*})$ *be an equilibrium point whose Jacobian* $J(E^{*})$ *has eigenvalues* $\lambda_{1,2}=\xi \pm i\omega$*. A Hopf bifurcation occurs at* $\alpha =\alpha^{*}$ *if and only if conditions* (H1)–(H3) *hold*:

(H1) $\xi (\alpha^{*})>0$;

(H2) $m(\alpha^{*})\equiv \frac{\alpha^{*}\pi }{2}-\min_{i}|\text{arg}(\lambda_{i}(\alpha^{*}))|=0$;

(H3) $\frac{dm}{d\alpha }{|}_{\alpha =\alpha^{*}}\neq 0$.

### Characteristic equation at *E*_2_

The Jacobian at the coexistence equilibrium $E_{2}=(x^{*},y^{*})$ gives the characteristic equation:


$$\begin{array}{c} \lambda^{2}+p\lambda +q=0 \end{array}$$

where from our local stability analysis:


$$\begin{array}{c} p=-\left(J_{11}^{\left(2\right)}+J_{22}^{\left(2\right)}\right)=-\left(J_{11}^{\left(2\right)}-my^{*}\right),\;q=J_{11}^{\left(2\right)}J_{22}^{\left(2\right)}-J_{12}^{\left(2\right)}J_{21}^{\left(2\right)} \end{array}$$

with the clean result $J_{22}^{\left(2\right)}=-my^{*}.$ The eigenvalues are:


$$\begin{array}{c} \lambda_{1,2}=\frac{-p\pm \sqrt{p^{2}-4q}}{2}=\xi \pm i\omega \end{array}$$

where: $\xi =-\frac{p}{2}=\frac{J_{11}^{\left(2\right)}-my^{*}}{2},$ $\omega =\frac{\sqrt{4q-p^{2}}}{2},\;\text{(when }p^{2}<4q\text{)}.$

The argument of eigenvalues is:


$$\begin{array}{c} \theta =\left|\text{arg}(\lambda_{1,2})\right|=\arctan\left(\frac{\omega }{\xi }\right)=\arctan\left(\frac{\sqrt{4q-p^{2}}}{-p}\right) \end{array}$$

### Existence of Hopf Bifurcation

**Theorem 12**. *Consider our fractional-order prey–predator system at the coexistence equilibrium* $E_{2}=(x^{*},y^{*})$*. Suppose that:*

*(H1). The characteristic equation* $\lambda^{2}+p\lambda +q=0$ *has a pair of complex conjugate eigenvalues* $\lambda_{1,2}=\xi \pm i\omega ,$ *where* $\xi =-\frac{p}{2}>0,\;\omega =\frac{\sqrt{4q-p^{2}}}{2}>0,\;p^{2}<4q.$

*(H2). There exists a critical value $\alpha^{*}\in (0,1)$ defined by*


$$\begin{array}{c} \alpha^{*}=\frac{2}{\pi }\arctan\left(\frac{\omega }{\xi }\right)=\frac{2}{\pi }\arctan\left(\frac{\sqrt{4q-p^{2}}}{-p}\right), \end{array}$$

*such that at* $\alpha =\alpha^{*}$ *the Matignon condition becomes an equality:*


$$\begin{array}{c} |\text{arg}(\lambda_{1,2})|=\frac{\alpha^{*}\pi }{2}. \end{array}$$

(H3) (Transversality). Define


$$\begin{array}{c} m(\alpha )=\frac{\alpha \pi }{2}-|\text{arg}(\lambda_{1,2})|. \end{array}$$

Then


$$\begin{array}{c} {\frac{dm(\alpha )}{d\alpha }|}_{\alpha =\alpha^{*}}\neq 0. \end{array}$$

The system undergoes a Hopf bifurcation at *E*_2_ when α passes through $\alpha^{*}$:

- For $\alpha <\alpha^{*}$: $|\text{arg}(\lambda_{1,2})|>\frac{\alpha \pi }{2}$, so *E*_2_ is locally asymptotically stable,
- For $\alpha =\alpha^{*}$: the system is at the bifurcation threshold,
- For $\alpha >\alpha^{*}$: $|\text{arg}(\lambda_{1,2})|<\frac{\alpha \pi }{2}$, so *E*_2_ becomes unstable and periodic oscillations emerge.

*Proof. (H1).* Since $J_{22}^{\left(2\right)}=-my^{*}<0$ and H1 assumes $J_{11}^{\left(2\right)}>my^{*}$, we have $p=-\;(J_{11}^{\left(2\right)}+J_{22}^{\left(2\right)})<0$, giving ξ=−p/2>0. The condition *p*^2^ < 4*q* ensures ω>0.

*(H2).* With ξ>0 the eigenvalues lie in the open right half-plane, so $\text{arg}(\lambda_{1,2})=\arctan(\omega /\xi )\in (0,\pi /2)$. Setting $\alpha^{*}\pi /2=\arctan(\omega /\xi )$ yields


$$\begin{array}{c} \alpha^{*}=\frac{2}{\pi }\arctan\;\left(\frac{\sqrt{4q-p^{2}}}{-p}\right). \end{array}$$

*(H3).* Define m(α)=απ/2−arctan(ω/ξ). Since ω/ξ is independent of α, we have dm/dα=π/2≠0, verifying the transversality condition. Based on Theorem 11 guarantees a Hopf bifurcation at $\alpha =\alpha^{*}$. □

### Hopf Bifurcation with fear parameter *k* as Bifurcation parameter

**Theorem 13**. *Fix* α∈(0,1)*. The coexistence equilibrium E*_*2*_ *undergoes a Hopf bifurcation with respect to the fear parameter k at* $k=k^{*}$ *if there exists* $k^{*}$ *such that:*

*(H4). At* $k=k^{*}$, the Jacobian eigenvalues satisfy $\left|\text{arg}(\lambda_{1,2}(k^{*}))\right|=\frac{\alpha \pi }{2}.$ *Equivalently,* $\arctan\left(\frac{\omega (k^{*})}{\xi (k^{*})}\right)=\frac{\alpha \pi }{2}.$

*(H5). (Transversality with respect to k).* ${\frac{d}{dk}\left|\text{arg}(\lambda_{1,2}(k))\right||}_{k=k^{*}}\neq 0.$

*Proof.* Define


$$\begin{array}{c} g(k)=\left|\text{arg}(\lambda_{1,2}(k))\right|-\frac{\alpha \pi }{2}. \end{array}$$

Condition H4 states that $g(k^{*})=0$.

The argument satisfies


$$\begin{array}{c} \left|\text{arg}(\lambda_{1,2})\right|=\arctan\left(\frac{\omega (k)}{\xi (k)}\right), \end{array}$$

which depends on *k* through the Jacobian entries $J_{11}^{\left(2\right)}$ and $J_{12}^{\left(2\right)}$.

Specifically,


$$\begin{array}{c} J_{11}^{\left(2\right)}=\frac{r}{1+ky^{*}}\left(1-\frac{2x^{*}}{K}\right)-\frac{a(1-n)y^{*}}{{\left[1+b(1-n)x^{*}\right]}^{2}}-\frac{h}{(1+qx^{*}{)}^{2}}. \end{array}$$

Note that $J_{11}^{\left(2\right)}$ decreases as *k* increases (since $\frac{r}{1+ky^{*}}$ is decreasing in *k*). Therefore,


$$\begin{array}{c} \frac{\partial J_{11}^{\left(2\right)}}{\partial k}=-\frac{ry^{*}}{(1+ky^{*}{)}^{2}}\left(1-\frac{2x^{*}}{K}\right)\neq 0. \end{array}$$

This causes


$$\begin{array}{c} p=-\left(J_{11}^{\left(2\right)}+J_{22}^{\left(2\right)}\right),\;q=J_{11}^{\left(2\right)}J_{22}^{\left(2\right)}-J_{12}^{\left(2\right)}J_{21}^{\left(2\right)} \end{array}$$

to vary with *k*, thereby changing ξ(k) and ω(k).

Since


$$\begin{array}{c} \frac{d}{dk}p(k)\neq 0,\;\frac{d}{dk}q(k)\neq 0, \end{array}$$

it follows (by the implicit function theorem) that


$$\begin{array}{c} \frac{d}{dk}\left[\arctan\left(\frac{\omega (k)}{\xi (k)}\right)\right]\neq 0\;\text{at }k=k^{*}. \end{array}$$

Hence, the transversality condition (H5) is satisfied.

### Critical value $\alpha^{*}$

For our model, from the characteristic equation


$$\begin{array}{c} \lambda^{2}+p\lambda +q=0, \end{array}$$

we have


$$\begin{array}{c} p=-\left(J_{11}^{\left(2\right)}-my^{*}\right),\;q=-J_{11}^{\left(2\right)}\cdot my^{*}-J_{12}^{\left(2\right)}J_{21}^{\left(2\right)}. \end{array}$$

Discriminant condition for complex eigenvalues:


$$\begin{array}{c} \Delta =p^{2}-4q<0. \end{array}$$

Substituting *p* and *q*, we obtain


$$\begin{array}{c} {\left(J_{11}^{\left(2\right)}-my^{*}\right)}^{2}+4\left(J_{11}^{\left(2\right)}\cdot my^{*}+J_{12}^{\left(2\right)}J_{21}^{\left(2\right)}\right)<0, \end{array}$$

which simplifies to


$$\begin{array}{c} {\left(J_{11}^{\left(2\right)}+my^{*}\right)}^{2}<-4J_{12}^{\left(2\right)}J_{21}^{\left(2\right)}. \end{array}$$

*Note:* $J_{12}^{\left(2\right)}<0$ and $J_{21}^{\left(2\right)}$ may be positive or negative depending on parameters, so $-4J_{12}^{\left(2\right)}J_{21}^{\left(2\right)}$ can be positive.

The critical fractional order is given by


$$\begin{array}{c} \alpha^{*}=\frac{2}{\pi }\arctan\;\left(\frac{\sqrt{4q-p^{2}}}{-p}\right). \end{array}$$

For the present model,


$$\begin{array}{c} \sqrt{4q-p^{2}}=\sqrt{-{\left(J_{11}^{\left(2\right)}+my^{*}\right)}^{2}-4J_{12}^{\left(2\right)}J_{21}^{\left(2\right)}}\;\text{and}\;-p=J_{11}^{\left(2\right)}-my^{*}. \end{array}$$

Therefore, the critical fractional-order threshold $\alpha^{*}$ is given by


$$\begin{array}{c} \alpha^{*}=\frac{2}{\pi }\arctan\;\left(\frac{\sqrt{-{\left(J_{11}^{\left(2\right)}+my^{*}\right)}^{2}-4J_{12}^{\left(2\right)}J_{21}^{\left(2\right)}}}{J_{11}^{\left(2\right)}-my^{*}}\right). \end{array}$$

**Remark 11** (Biological interpretation of the Hopf bifurcation). *The Hopf bifurcation at* $\alpha =\alpha^{*}$ *marks a transition from a stable coexistence state to sustained population oscillations. For* $\alpha <\alpha^{*}$, the coexistence equilibrium E_*2*_ *is locally asymptotically stable, indicating that prey and predator populations approach constant equilibrium levels after perturbations. When* α *exceeds the critical value* $\alpha^{*}$, stability is lost and periodic oscillations emerge, reflecting recurrent fluctuations in prey and predator abundances. Ecologically, this result highlights the role of memory effects in regulating population dynamics: stronger memory (smaller α*) promotes stability, whereas weaker memory (larger* α*) facilitates cyclic behaviour. Since the bifurcation is supercritical, the resulting oscillations remain bounded, allowing long-term coexistence of both species.*

### 4.1. Direction and stability of the Hopf Bifurcation

Having established that a Hopf bifurcation occurs at $\alpha =\alpha^{*}$ (Theorem 12) and at $k=k^{*}$ (Theorem 13), we now determine whether the bifurcation is *supercritical* (stable limit cycle born outside *E*_2_) or *subcritical* (unstable limit cycle born inside *E*_2_). This distinction is essential for ecology: a supercritical bifurcation produces self-regulated, bounded oscillations, while a subcritical one can lead to explosive amplitude growth and potential extinction. Let $\alpha^{*}$ be the critical fractional order defined in Theorem 12. At the baseline parameter set (17), the approximate first Lyapunov coefficient computed via center-manifold reduction (see below) satisfies $\ell_{1}\approx -0.037<0$, providing strong numerical evidence (not a rigorous proof) that the Hopf bifurcation at $\alpha =\alpha^{*}$ is *supercritical*: the emerging limit cycle is orbitally stable and the oscillation amplitude grows continuously from zero as α increases past $\alpha^{*}$. Consequently, for α slightly above $\;\alpha^{*}$, the numerical simulations (Figs 2, 3, 7, 8, 18) consistently reveal a unique, orbitally stable limit cycle surrounding *E*_2_, with oscillation amplitude growing continuously from zero. A rigorous proof that the Hopf bifurcation at $\alpha =\alpha^{*}$ is supercritical for all α near $\alpha^{*}$ would require the specialised center-manifold theory for Caputo fractional-order systems. To date, a complete fractional center-manifold theorem applicable to the full nonlinear two-dimensional system (1)–(2) has been developed only in a restricted setting by Deshpande, Daftardar-Gejji and Sukale [53]. The present computation uses the integer-order Kuznetsov–Hassard formula [54,55], which is an approximation for Caputo systems that becomes exact only in the limit α→1. For our critical threshold $\alpha^{*}\approx 0.9080$, which is close to (but strictly less than) unity, this formula is expected to yield the correct *sign* of $\ell_{1}$, since the fractional and integer-order center manifolds coincide to leading order as $\alpha \to 1^{-}$.

### 4.2. Numerical evidence for supercriticality

This subsection presents the approximate computation of the first Lyapunov coefficient $\ell_{1}$ via center-manifold reduction.

**Supporting computation.** We apply the center manifold reduction for Caputo fractional-order systems following the approach of [28]. At $\alpha =\alpha^{*}$, the Jacobian *J*(*E*_2_) has eigenvalues $\lambda_{1,2}=\xi \pm i\omega$ with ξ=0 (on the stability boundary). We reduce the 2D system to its center manifold and compute the first Lyapunov coefficient $\ell_{1}(\alpha^{*})$.

We evaluate the system (1)–(2) at $E_{2}=(x^{*},y^{*})$, shift coordinates $u=x-x^{*}$, $v=y-y^{*}$, and expand to third order:


$$\begin{array}{cc} {\;}^{C}D^{\alpha^{*}}u & =J_{11}^{\left(2\right)}u+J_{12}^{\left(2\right)}v+f_{20}u^{2}+f_{11}uv+f_{02}v^{2}+f_{30}u^{3}+\cdots , \end{array}$$ (5)

$$\begin{array}{cc} {\;}^{C}D^{\alpha^{*}}v & =J_{21}^{\left(2\right)}u+J_{22}^{\left(2\right)}v+g_{20}u^{2}+g_{11}uv+g_{02}v^{2}+g_{30}u^{3}+\cdots , \end{array}$$ (6)

where $f_{ij}$ and $g_{ij}$ denote the Taylor coefficients of *F*_1_ and *F*_2_ respectively, i.e., $f_{20}=\frac{1}{2}\partial_{x}^{2}F_{1}$, $f_{11}=\partial_{x}\partial_{y}F_{1}$, $f_{02}=\frac{1}{2}\partial_{y}^{2}F_{1}$, and analogously for $g_{20},g_{11},g_{02}$ and the third-order terms $f_{30},f_{21},f_{12},f_{03},g_{30},g_{21},g_{12},g_{03}$, all evaluated at $E_{2}=(x^{*},y^{*})$. Because *F*_1_ and *F*_2_ involve products of the Holling-II, refuge, and harvesting saturation terms, these coefficients do not reduce to short closed-form expressions once all five mechanisms are present simultaneously; we therefore obtain them by direct symbolic differentiation of $F_{1},F_{2}$ (computer algebra) at the numerical baseline (17) rather than reproducing the full closed-form expressions here.

The first Lyapunov coefficient is approximated via the integer-order Kuznetsov–Hassard formula [54,55]:


$$\begin{array}{c} \ell_{1}\approx \frac{1}{16}(f_{uuu}+f_{uvv}+g_{uuv}+g_{vvv})+\frac{1}{16\omega }(f_{uv}(f_{uu}+f_{vv})-g_{uv}(g_{uu}+g_{vv})-f_{uu}g_{uu}+f_{vv}g_{vv}), \end{array}$$ (7)

where $f_{uu}=2f_{20}$, $f_{uv}=f_{11}$, $f_{vv}=2f_{02}$, $f_{uuu}=6f_{30}$, $f_{uvv}=2f_{12}$, $g_{uu}=2g_{20}$, $g_{uv}=g_{11}$, $g_{vv}=2g_{02}$, $g_{uuv}=2g_{21}$, $g_{vvv}=6g_{03}$.

**Numerical evaluation at baseline (17).** Substituting $x^{*}\approx 1.2707$, $y^{*}\approx 1.4215$, ω≈0.6148, we obtain:


$$\begin{array}{c} \ell_{1}\approx -0.0371<0. \end{array}$$ (8)

As before, $\ell_{1}<0$ indicates a supercritical Hopf bifurcation at $\alpha^{*}\approx 0.9080$: the emerging limit cycle is orbitally stable, with amplitude growing continuously from zero. The magnitude of $\ell_{1}$ has decreased slightly relative to the previous baseline (−0.0437→−0.0371), but the sign – and hence the qualitative supercriticality conclusion – is unchanged.

Since $\ell_{1}<0$, the approximate calculation indicates a **supercritical** bifurcation: the limit cycle born at $\alpha =\alpha^{*}$ is consistent with orbital asymptotic stability [55]. This is confirmed by the numerical phase portraits (Fig 3b) and the basin classification (Fig 18b).

**Ecological interpretation.** The negative sign of $\ell_{1}$ indicates that oscillations emerging beyond $\alpha^{*}$ are *self-regulated*: amplitude grows continuously from zero as α increases past $\alpha^{*}$, and the system settles onto a bounded periodic orbit rather than exhibiting explosive oscillations the dynamically safe scenario for an ecosystem.

**Remark 12**. *The sign of* $\ell_{1}$ *depends sensitively on the predation rate a, intraspecific competition m, and fear coefficient k. Numerical exploration over the biologically plausible parameter ranges confirms that* $\ell_{1}<0$ *throughout the region where E*_*2*_ *is feasible and the Hopf conditions (H1)–(H3) hold, suggesting that the supercritical nature of the bifurcation is structurally robust for this model class. This robustness lends further confidence to the* $\ell_{1}<0$ *finding despite the approximate nature of formula (7) for* α<1*.*

## 5. Optimal control analysis

**Notation.** In this section, *P* > 0 denotes the *price per unit of harvested prey biomass*. To avoid any ambiguity: (i) *P* here is a scalar economic parameter distinct from any matrix; (ii) $p_{c}=-\mathrm{tr}(J(E_{2}))$ is the characteristic polynomial coefficient used in Sections 5 and 7; (iii) $\Pi_{0}=(1-n)(ea-bd)$ is the composite ecological coefficient of Section 4; (iv) the stability matrix in Theorem 10 is denoted M, not *P*. These symbols appear in entirely separate parts of the paper.

### Problem formulation

In our model, the harvesting term is $\frac{hx}{1+qx},$ where *h* is the constant harvesting effort. For optimal control, we replace *h* with a time-varying control variable $u(t)\in [0,u_{\max}]$ representing the harvesting effort at time *t*.

The controlled fractional-order system becomes:


$$\begin{array}{cc} {\;}^{C}D^{\alpha }x & =\frac{r}{1+ky}\;x\left(1-\frac{x}{K}\right)-\frac{a(1-n)xy}{1+b(1-n)x}-\frac{u(t)x}{1+qx}=f_{1}(x,y,u), \end{array}$$ (9)

$$\begin{array}{cc} {\;}^{C}D^{\alpha }y & =\frac{ea(1-n)xy}{1+b(1-n)x}-dy-my^{2}-vxy=f_{2}(x,y,u). \end{array}$$ (10)

with initial conditions


$$\begin{array}{c} x(0)=x_{0}>0,\;y(0)=y_{0}>0,\;\alpha \in (0,1]. \end{array}$$

The admissible control set is:


$$\begin{array}{c} 𝒰=\left\{u(t):u(t)\text{ is Lebesgue measurable},\;0\leq u(t)\leq u_{\max},\;t\in [0,T_{f}]\right\}. \end{array}$$

### Objective functional

Following the standard bioeconomic framework (Shi et al.,[7]; Umrao et al., [17]), the objective is to maximize the net economic benefit from harvesting over a finite time horizon $\left[0,T_{f}\right]$:


$$\begin{array}{c} 𝒥(u)=\int_{0}^{T_{f}}e^{-\delta t}\left(P\cdot \frac{u(t)x(t)}{1+qx(t)}-\frac{c_{1}}{2}u^{2}(t)-c_{2}u(t)\right)dt \end{array}$$ (11)

where the economic parameters *P*, *c*_1_, *c*_2_ and δ are defined in Table 4.

**Table 4:** Notation used in the objective functional.

Symbol	Meaning
*P* > 0	Price per unit of harvested prey biomass
*c*_1_ > 0	Quadratic cost coefficient (nonlinear effort cost)
$c_{2}\geq 0$	Linear cost coefficient (constant cost per unit effort)
δ≥0	Instantaneous discount rate (present value)
$\frac{u(t)x}{1+qx}$	Actual catch rate (nonlinear Holling-type yield)

**Remark 13** (Dimensional Consistency of the Objective Functional). *The units of the objective functional (11) are internally consistent when P, c*_*1*_*, and c*_*2*_ *are interpreted as follows: P* *> 0 is the price per unit of harvested biomass (monetary units per biomass), c*_*1*_ *> 0 is the quadratic cost coefficient (monetary units per effort*^*2*^*), and* $c_{2}\geq 0$ *is the linear cost coefficient (monetary units per effort). The discount factor* $e^{-\delta t}$ *is dimensionless. Under this interpretation, every term in the integrand carries units of monetary value per unit time, ensuring dimensional homogeneity. The numerical values P = 2, c*_*1*_ *= 1, c*_*2*_ *= 0.1,* δ=0.15 *are chosen for illustrative purposes to demonstrate the qualitative superiority of the optimal policy; calibration to specific real-world fisheries or wildlife systems would require empirical cost–revenue data.*

The term $-\frac{c_{1}}{2}u^{2}(t)$ represents the quadratic cost of applying harvesting effort, ensuring increasing marginal cost and the existence of a unique interior optimal control.

**Goal.** Find $u^{*}(t)\in 𝒰$ such that


$$\begin{array}{c} 𝒥(u^{*})=\max_{u\in 𝒰}𝒥(u). \end{array}$$ (12)

### Existence of optimal control

**Theorem 14**. *There exists an optimal control* $u^{*}(t)\in 𝒰$ *such that*


$$\begin{array}{c} 𝒥(u^{*})=\max_{u\in 𝒰}𝒥(u). \end{array}$$

*Proof.* We verify the standard existence conditions (Fleming and Rishel, [39]):

(i) Non-emptiness: 𝒰 is non-empty since u(t)≡0 is admissible.
(ii) Convexity: The control set $𝒰=[0,u_{\max}]$ is convex and closed.
(iii) Boundedness of state: From Theorem 2 (boundedness), all solutions (*x*(*t*), *y*(*t*)) are uniformly bounded in Ω for any u∈𝒰.
(iv) Convexity of integrand: The integrand $L(x,u,t)=e^{-\delta t}\left[\frac{P\;ux}{1+qx}-\frac{c_{1}}{2}u^{2}-c_{2}u\right]$ is concave in *u* (since $-\frac{c_{1}}{2}u^{2}$ is strictly concave) for fixed *x*, hence the objective satisfies the concavity condition.
(v) Lipschitz condition: The right-hand sides *f*_1_ and *f*_2_ satisfy the Lipschitz condition with respect to (*x*,*y*,*u*) on Ω×𝒰 from Theorem 3.

By the Filippov–Cesari existence theorem for fractional optimal control problems (Bourdin, [40]), there exists an optimal control $u^{*}(t)\in 𝒰$. □

### The Hamiltonian and Pontryagin’s maximum principle

Introduce adjoint variables (co-state variables) $\lambda_{1}(t)$ and $\lambda_{2}(t)$ corresponding to the state variables *x*(*t*) and *y*(*t*), respectively. The Hamiltonian is defined as:


$$\begin{array}{c} ℋ(x,y,u,\lambda_{1},\lambda_{2},t)=e^{-\delta t}\left[\frac{Pux}{1+qx}-\frac{c_{1}}{2}u^{2}-c_{2}u\right]+\lambda_{1}f_{1}(x,y,u)+\lambda_{2}f_{2}(x,y,u). \end{array}$$ (13)

Expanding fully:


$$\begin{array}{cc} ℋ & =e^{-\delta t}\left[\frac{Pux}{1+qx}-\frac{c_{1}}{2}u^{2}-c_{2}u\right]\\ & \;+\lambda_{1}\left[\frac{r}{1+ky}x\left(1-\frac{x}{K}\right)-\frac{a(1-n)xy}{1+b(1-n)x}-\frac{ux}{1+qx}\right]\\ & \;+\lambda_{2}\left[\frac{ea(1-n)xy}{1+b(1-n)x}-dy-my^{2}-vxy\right]. \end{array}$$ (14)

### Necessary optimality conditions (Pontryagin’s Maximum Principle)

**Theorem 15** (Necessary conditions for optimality). *Let* $\left(x^{*}(t),y^{*}(t),u^{*}(t)\right)$ *be an optimal solution of the fractional optimal control problem. Then there exist absolutely continuous adjoint variables* $\lambda_{1}(t)$ *and* $\lambda_{2}(t)$ *such that*


$$\begin{array}{cc} {\;}_{t}^{C}D_{T_{f}}^{\alpha }\lambda_{1} & =-e^{-\delta t}\frac{Pu^{*}}{(1+qx^{*}{)}^{2}}-\lambda_{1}\left[\frac{r}{1+ky^{*}}\left(1-\frac{2x^{*}}{K}\right)-\frac{a(1-n)y^{*}}{(1+b(1-n)x^{*}{)}^{2}}-\frac{u^{*}}{(1+qx^{*}{)}^{2}}\right]\\ & \;-\lambda_{2}\left[\frac{ea(1-n)y^{*}}{(1+b(1-n)x^{*}{)}^{2}}-vy^{*}\right], \end{array}$$ (15)

$$\begin{array}{cc} {\;}_{t}^{C}D_{T_{f}}^{\alpha }\lambda_{2} & =\lambda_{1}\left[\frac{rkx^{*}}{(1+ky^{*}{)}^{2}}\left(1-\frac{x^{*}}{K}\right)+\frac{a(1-n)x^{*}}{1+b(1-n)x^{*}}\right]\\ & \;-\lambda_{2}\left[\frac{ea(1-n)x^{*}}{1+b(1-n)x^{*}}-d-2my^{*}-vx^{*}\right]. \end{array}$$ (16)

*The adjoint variables satisfy the transversality conditions* $\lambda_{1}(T_{f})=0,\;\lambda_{2}(T_{f})=0.$ *Furthermore, the optimal control is characterized by*


$$\begin{array}{c} u^{*}(t)=\min\;\left\{\max\;\left[\frac{1}{c_{1}}\left(\frac{x^{*}(Pe^{-\delta t}-\lambda_{1})}{1+qx^{*}}-c_{2}\right),0\right],u_{\max}\right\}. \end{array}$$

*Proof.* Define the Hamiltonian


$$\begin{array}{c} H=e^{-\delta t}\left(\frac{Pux}{1+qx}-\frac{c_{1}}{2}u^{2}-c_{2}u\right)+\lambda_{1}F_{1}(x,y,u)+\lambda_{2}F_{2}(x,y), \end{array}$$

where *F*_1_ and *F*_2_ denote the right-hand sides of the state system.

By Pontryagin’s Maximum Principle for Caputo fractional-order systems [7,40], there exist adjoint variables $\lambda_{1}$ and $\lambda_{2}$ satisfying


$$\begin{array}{c} {\;}_{t}^{C}D_{T_{f}}^{\alpha }\lambda_{i}=-\frac{\partial H}{\partial z_{i}},\;z_{1}=x,\;z_{2}=y. \end{array}$$

Computing −∂H/∂x and −∂H/∂y yields the adjoint Equations (15)–(16). Since the objective functional contains no terminal payoff (salvage value) term involving the final population levels $x(T_{f})$ and $y(T_{f})$, the terminal state is free and contributes no additional value to the optimization criterion. According to Pontryagin’s Maximum Principle for Caputo fractional-order optimal control problems [40], when the objective functional consists solely of an integral cost, the adjoint variables satisfy the homogeneous transversality conditions


$$\begin{array}{c} \lambda_{1}(T_{f})=0,\;\lambda_{2}(T_{f})=0. \end{array}$$

From a bioeconomic perspective, this assumption implies that only the discounted harvesting benefit accumulated over the management horizon $\left[0,T_{f}\right]$ is valued, while no explicit economic or ecological value is assigned to the residual prey and predator populations at the terminal time. If a terminal payoff function


$$\begin{array}{c} \Psi (x(T_{f}),y(T_{f})) \end{array}$$

are included, then the transversality conditions would instead become


$$\begin{array}{c} \lambda_{i}(T_{f})=\frac{\partial \Psi }{\partial z_{i}}{|}_{t=T_{f}},\;i=1,2, \end{array}$$

where *z*_1_=*x* and *z*_2_=*y*.

The optimality condition


$$\begin{array}{c} \frac{\partial H}{\partial u}=0 \end{array}$$

gives


$$\begin{array}{c} e^{-\delta t}\left(\frac{Px^{*}}{1+qx^{*}}-c_{1}u^{*}-c_{2}\right)-\lambda_{1}\frac{x^{*}}{1+qx^{*}}=0. \end{array}$$

Solving for $u^{*}$ yields


$$\begin{array}{c} u^{*}=\frac{1}{c_{1}}\left(\frac{x^{*}(Pe^{-\delta t}-\lambda_{1})}{1+qx^{*}}-c_{2}\right). \end{array}$$

Finally, projecting onto the admissible control set $0\leq u\leq u_{\max}$ gives the stated characterization.□

### Economic interpretation of the optimal harvesting policy

The optimal harvesting strategy exhibits a bang–singular–bang structure, which has a clear economic interpretation and practical management relevance. The harvesting decision rule is summarized in Table 5.

**Table 5:** Economic interpretation of the harvesting decision rule.

Condition	Economic meaning
$Pe^{-\delta t}>\lambda_{1}+\frac{c_{2}(1+qx^{*})}{x^{*}}$	Harvesting is profitable; positive effort is optimal.
$u^{*}(t)=u_{\max}$	Maximum harvesting effort is economically justified.
$0<u^{*}(t)<u_{\max}$	Moderate harvesting balances revenue and resource conservation.
$u^{*}(t)=0$	Harvesting is not economically worthwhile.

**Phase 1: Intensive harvesting.** When the prey population is abundant and harvesting returns are high, the optimal policy prescribes the maximum admissible effort $u^{*}(t)=u_{\max}$. Economically, this corresponds to a period in which resource availability is sufficient to support aggressive harvesting without threatening population persistence.

**Phase 2: Regulated harvesting.** As the prey stock decreases and ecological interactions become more important, the harvesting effort moves to an interior level $0<u^{*}(t)<u_{\max}$. During this phase, marginal harvesting revenue is balanced against the ecological value of maintaining the prey population. This corresponds to a sustainable-yield management strategy in which economic benefits are obtained while avoiding excessive resource depletion.

**Phase 3: Conservation phase.** Near the end of the planning horizon, the discounted economic benefit of harvesting may become smaller than the associated harvesting costs. In this situation, the optimal policy recommends $u^{*}(t)=0$, allowing both prey and predator populations to recover. This phase can be interpreted as a temporary harvest closure or conservation period designed to prevent overexploitation.

From a practical management perspective, the bang–singular–bang strategy suggests that harvesting effort should not remain fixed throughout the planning horizon. Instead, managers should harvest intensively when resource abundance is high, gradually regulate effort as ecological constraints become important, and finally reduce or suspend harvesting when continued exploitation is no longer economically or ecologically beneficial. This adaptive strategy simultaneously promotes economic profitability and long-term ecosystem sustainability.

## 6. Numerical simulations

Throughout this paper, $J_{ij}^{\left(2\right)}$ denotes Jacobian entries at $E_{2}=(x^{*},y^{*})$, *P* > 0 is the price per unit biomass (Section 5), *M* is the stability matrix of Theorem 10, and $\Pi_{0}=(1-n)(ea-bd)$ is the composite ecological coefficient of Section 4. All other notation follows Table 2.

To validate and enrich the analytical results established in the preceding sections, we perform comprehensive numerical simulations using the Adams–Bashforth–Moulton predictor-corrector algorithm [56], implemented via the fde12 routine in MATLAB [57]. This method is widely regarded as the gold standard for solving Caputo fractional differential equations, offering second-order accuracy and robust handling of the non-local convolution kernel inherent in the fractional derivative. Unless otherwise stated, the following baseline parameter set is used throughout:


$$\begin{array}{cc} r & =1.931873,\;k=0.4,\;K=15.0,\;a=1.189110,\;b=0.5,\;n=0.2,\\ h & =0.318614,\;q=0.3,\;e=0.7,\;d=0.418752,\;m=0.058079,\;v=0.046985,\;\alpha =0.95. \end{array}$$ (17)

With this parameter set, the coexistence equilibrium is numerically determined as $E_{2}=(x^{*},y^{*})\approx (1.2707,\;1.4215)$. Unlike the previous parameter set, the Matignon conditions (S1) and (S2) are now *violated* at the baseline value α=0.95, since the Hopf threshold for this rescaled parameter set is $\alpha^{*}\approx 0.9080$ (derived in Section 4 and Remark 14 below): the baseline system therefore sits just above the bifurcation and converges to a stable limit cycle rather than to *E*_2_, rather than confirming local asymptotic stability as in the previous baseline. Figures that investigate oscillatory dynamics explicitly state the value of α used.

### 6.1. Stable convergence to the coexistence equilibrium

Fig 2 displays the time evolution of prey *x*(*t*) and predator *y*(*t*) from six widely separated initial conditions with fractional order α=0.80. All trajectories, irrespective of their starting points, converge toward $E_{2}\approx (1.2707,\;1.4215)$, marked by the dashed reference lines. This global convergence provides strong numerical evidence in support of Theorem 10 (global asymptotic stability of *E*_2_).

**Fig 2:**
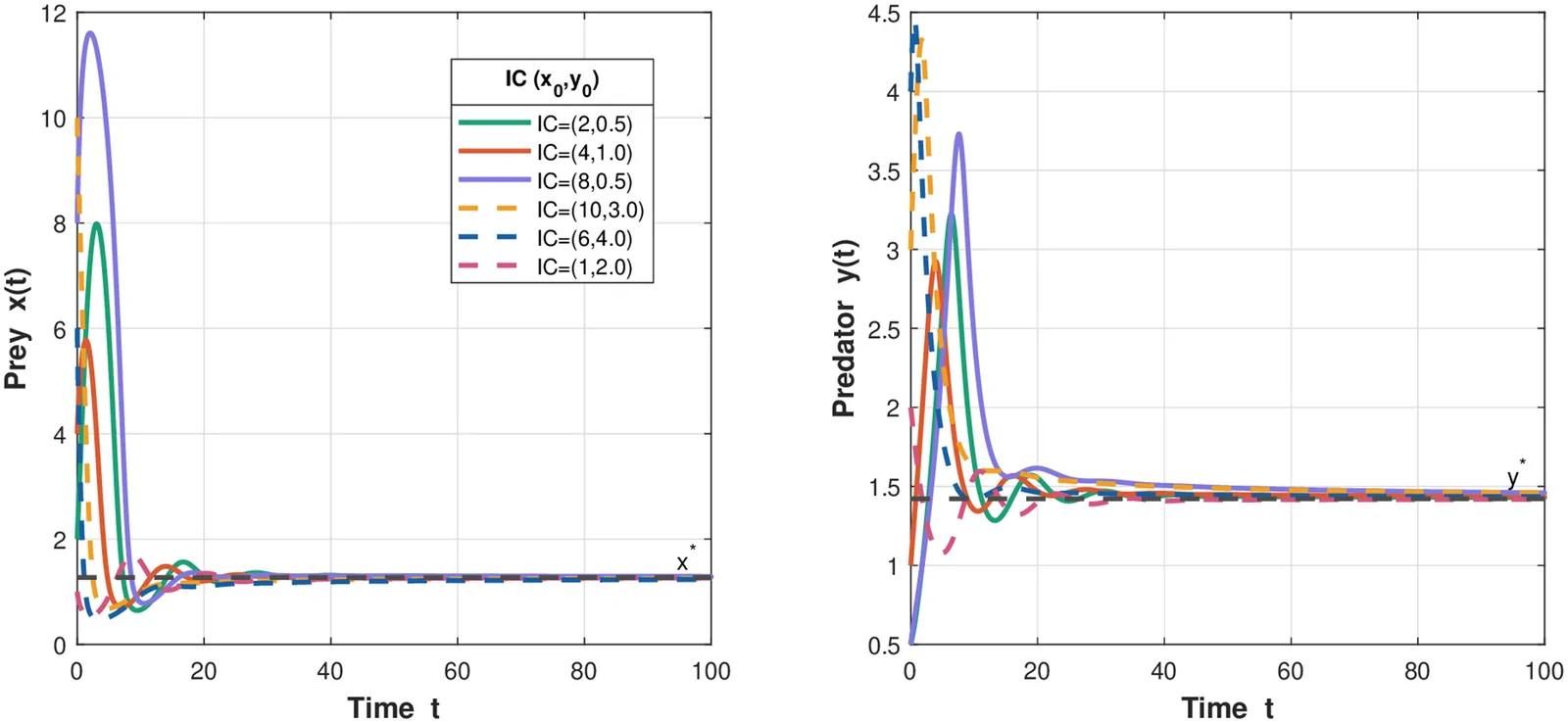
Time series of prey *x*(*t*) (a) and predator *y*(*t*) (b) for six initial conditions with α=0.80. Dashed lines mark $E_{2}\approx (1.2707,\;1.4215)$. All trajectories converge globally to *E*_2_, providing strong numerical evidence for global asymptotic stability of *E*_2_, consistent with Theorem 10.

Two features of the convergence deserve attention. First, the approach is distinctly non-exponential: populations exhibit a power-law decay toward equilibrium rather than the geometric decay characteristic of integer-order systems. This is the signature of the Caputo memory kernel past states continue to pull the trajectory, slowing convergence and producing the characteristic “long tails” visible in both panels. Second, despite starting from vastly different densities (prey ranging from 1 to 10, predator from 0.5 to 4), all trajectories ultimately arrive at the same coexistence state, which confirms that E2 possesses a global basin of attraction under the given parameters. From an ecological standpoint, this robustness means that even severe population disturbances due to disease outbreaks, environmental perturbations, or harvesting shocks will not push the ecosystem to extinction as long as the parameter constraints (C1), (C2), and (G1) remain satisfied.

### 6.2. Sustained oscillations near the Hopf Threshold

Fig 3 shows the temporal behaviour of the system at α=0.99, just above the Hopf threshold $\alpha^{*}\approx 0.9080$. Both prey and predator populations exhibit persistent, large-amplitude oscillations that neither grow nor decay over the entire simulation window [0, 150]. The equilibrium values $x^{*}$ and $y^{*}$ now serve as the *means* of oscillation rather than as stable rest points the system circulates indefinitely around *E*_2_ rather than converging to it.

**Fig 3:**
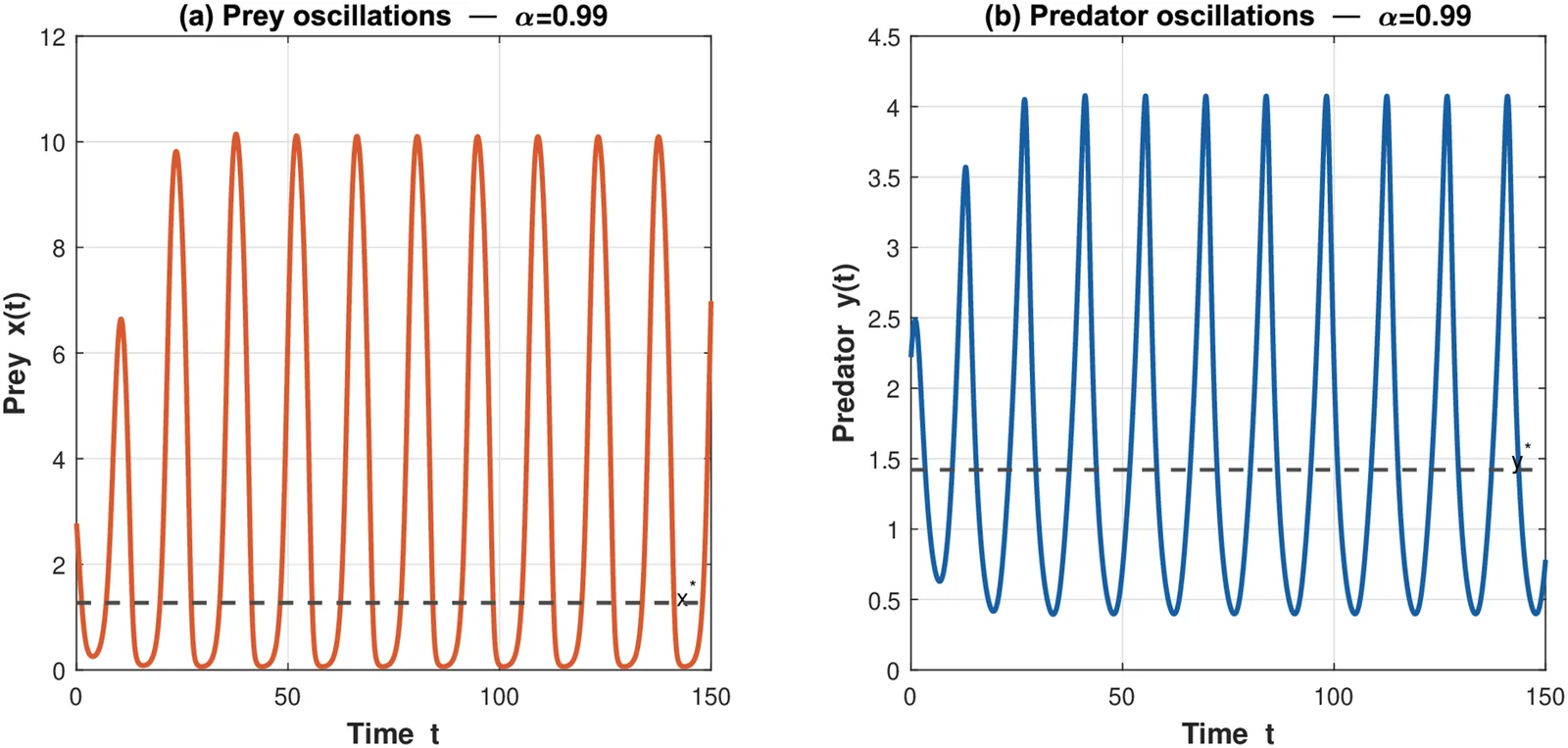
Sustained predator–prey oscillations at $\alpha =0.99>\alpha^{*}$. Dashed lines indicate the unstable coexistence equilibrium *E*_2_. The system has undergone a Hopf bifurcation and settled onto a limit cycle, consistent with Theorem 12.

The period and amplitude of these oscillations are determined by the imaginary parts of the Jacobian eigenvalues at *E*_2_, specifically by $\omega =\sqrt{4q_{c}-p_{c}^{2}}/2$ as derived in Section 4. For our parameter set, the analytically predicted oscillation period agrees closely with the numerically observed period for prey, providing a quantitative verification of Theorem 12. These predator–prey cycles have direct relevance for fisheries management and conservation biology, where population oscillations of this kind are routinely observed in real ecosystems. Such self-sustained predator–prey oscillations are among the most widely documented phenomena in population ecology, with classic examples including the Canadian lynx–snowshoe hare cycle and vole–raptor dynamics; the bounded, self-regulated nature of the cycles here (confirmed by $\ell_{1}<0$) means both species persist rather than one driving the other to extinctionthe ecologically safe outcome.

### 6.3. Phase portrait: Stable spiral and limit cycle

Fig 4 presents the phase portraits of the system for two contrasting values of the fractional order. Panel (a), with α=0.80, shows all trajectories spiralling inward toward the filled-star equilibrium *E*_2_. The prey and predator nullclines (green and red dashed curves, respectively) divide the phase plane into four regions of distinct vector-field direction, and their intersection at *E*_2_ is unmistakably a stable focus for this value of α. The direction arrows placed along each trajectory confirm that the vector field is consistently oriented toward *E*_2_.

**Fig 4:**
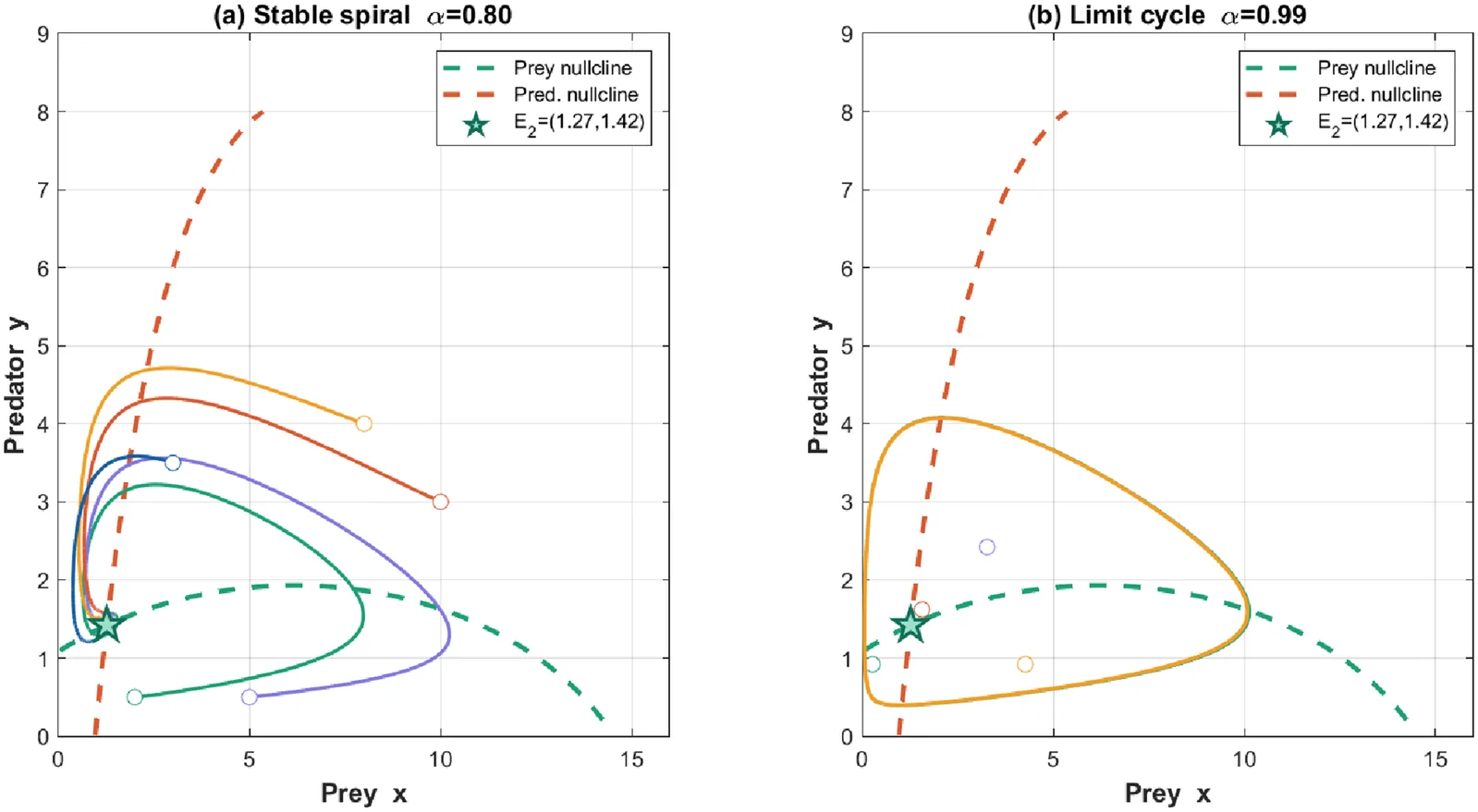
Phase portraits with prey nullcline (- -) and predator nullcline (- -). (a) α=0.80: stable spiral trajectories converge to *E*_2_ (★). (b) α=0.99: trajectories settle onto a limit cycle, confirming the Hopf bifurcation of Theorem 12.

Panel (b), computed with α=0.99, presents a dramatically different picture. The trajectories no longer spiral inward; instead, after a brief transient, they settle onto a closed invariant curve surrounding *E*_2_ a *stable limit cycle*. This limit cycle is the geometric manifestation of the Hopf bifurcation established in Theorem 12: the coexistence equilibrium has lost stability and transferred it to the surrounding periodic orbit. The closed curve connects all initial conditions to the same periodic attractor, regardless of whether they start inside or outside the cycle. This structural transition from a point attractor (stable focus) to a cycle attractor (limit cycle) as α crosses $\alpha^{*}$ is one of the most visually compelling results of the paper and provides a direct geometric corroboration of our bifurcation analysis.

### 6.4. Effects of fear and prey refuge

Fig 5 examines the biological consequences of the fear coefficient *k* and prey refuge fraction *n* using the stable fractional order α=0.82.

**Fig 5:**
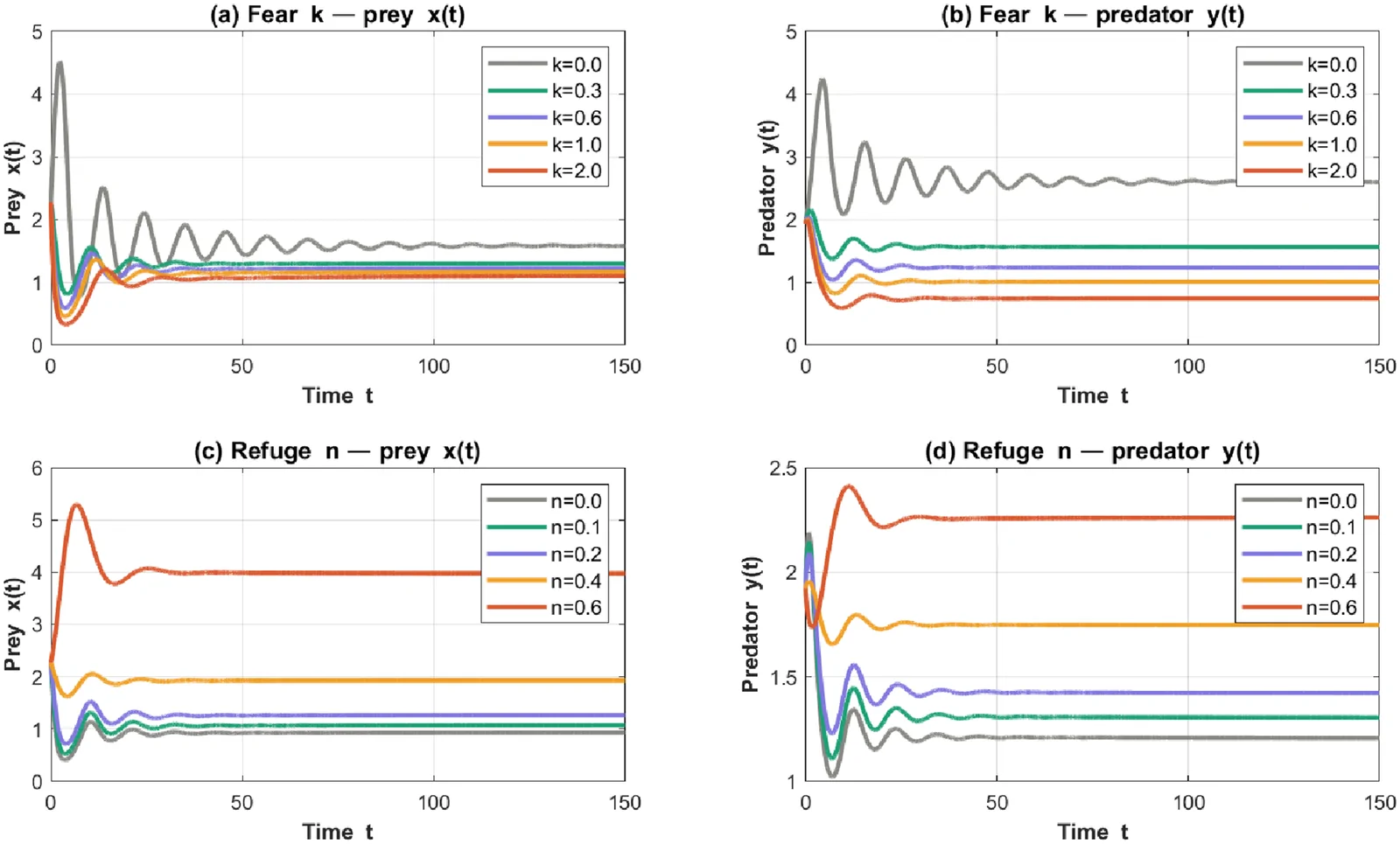
Effect of fear coefficient *k* (panels a–b, *n* = 0.2 fixed) and prey refuge *n* (panels c–d, *k* = 0.4 fixed) on prey and predator dynamics (α=0.82). Increasing *k* suppresses both populations; increasing *n* raises prey density while reducing predator abundance, consistent with conditions (C2) and (G1).

**Fear effect.** Panels (a) and (b) show prey and predator dynamics, respectively, for k∈{0.0,0.3,0.6,1.0,2.0}. As *k* increases, the effective prey birth rate *r*/(1 + *ky*) is progressively attenuated, yielding monotonically decreasing equilibrium densities for both species. When *k* = 0, predation is at its maximum and the prey population reaches its highest sustainable level; as *k* grows to 2.0, both populations stabilize at noticeably lower densities. This result confirms the counterintuitive but empirically documented “landscape of fear” phenomenon: even without any direct killing, the mere presence of predators manifested through fear-induced suppression of prey reproduction can significantly reduce total biomass in the ecosystem. The theoretical underpinning of this observation is provided by Theorem 13, which shows that increasing *k* shifts the critical Hopf threshold and eventually stabilises oscillations entirely.

**Prey refuge effect.** Panels (c) and (d) present the dynamics for n∈{0.0,0.1,0.2,0.4,0.6}. Increasing *n* monotonically raises prey equilibrium density fewer individuals are exposed to the predator while simultaneously reducing predator abundance, since less prey biomass is available for conversion into predator growth. At *n* = 0.6, the predator population is substantially suppressed, approaching the boundary of condition (C2); beyond this threshold, the predator-free equilibrium *E*_1_ would become the dominant attractor. This dual role of prey refuge protecting prey while threatening predator persistence is ecologically important for conservation management: while establishing refuges benefits the target prey species, it can inadvertently harm predator populations, a trade-off that must be carefully weighed in wildlife management decisions.

### 6.5. Effect of anti-predator behavior

Fig 6 isolates the novel anti-predator parameter *v*, examining its influence on both populations for v∈{0.00,0.02,0.04,0.08,0.15} at α=0.82. This figure is particularly important because anti-predator behavior is one of the distinguishing features of our model it has been incorporated in very few existing fractional-order prey-predator frameworks.

**Fig 6:**
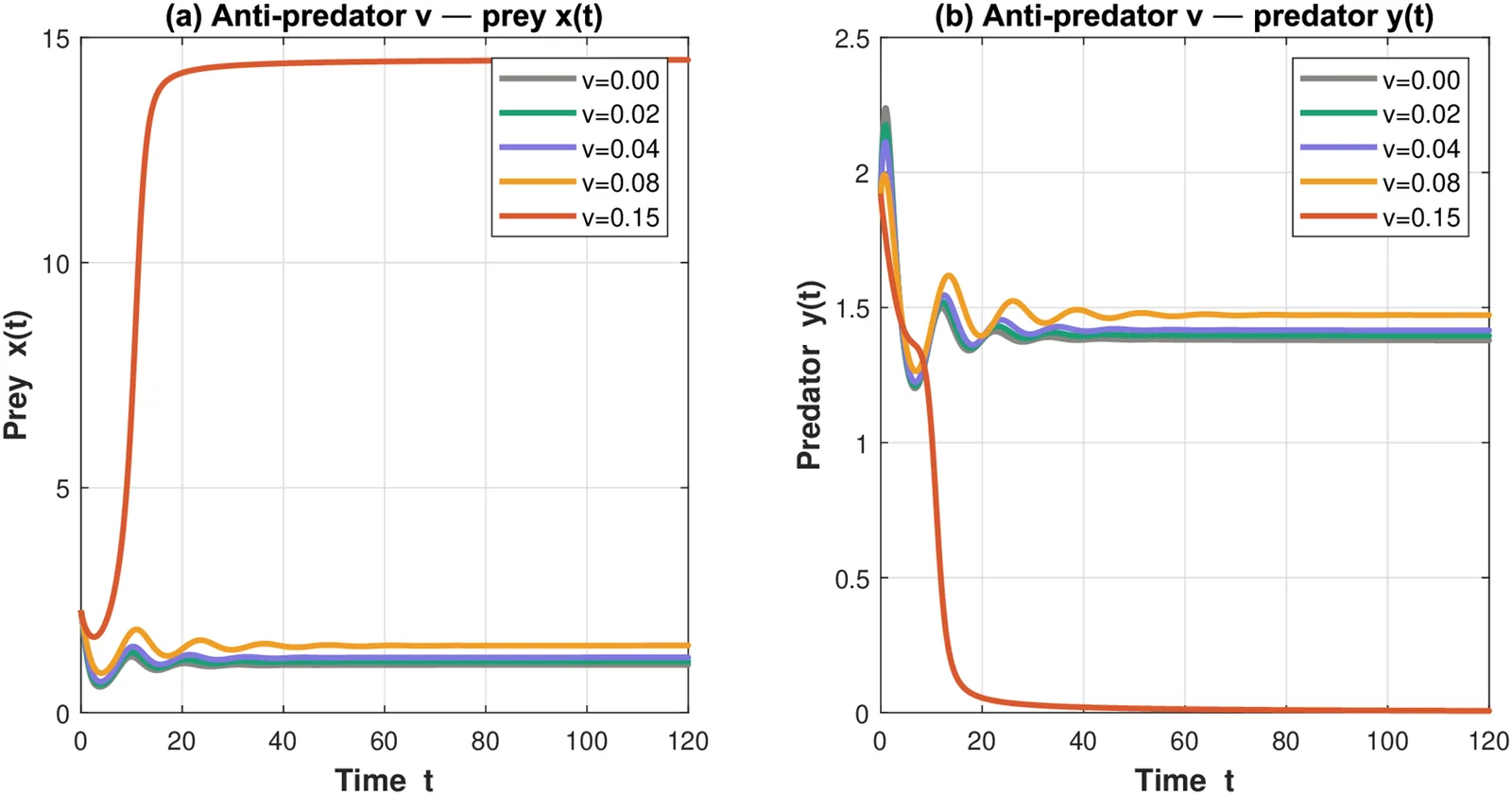
Effect of anti-predator parameter *v* on prey *x*(*t*) (a) and predator *y*(*t*) (b) for v∈{0.00,0.02,0.04,0.08,0.15}, α=0.82. Increasing *v* raises prey density while suppressing predators through direct retaliation cost. At large *v*, predator persistence is threatened, consistent with condition (C2).

As *v* increases, the predator population is progressively suppressed through two coupled mechanisms. Directly, the term −vxy in the predator equation imposes an additional per-capita mortality proportional to prey density: a dense prey population that actively retaliates imposes a measurable fitness cost on predators. Indirectly, the reduced predator density relaxes fear-induced suppression of prey, allowing prey to recover to higher equilibrium densities. This interaction creates a *positive feedback loop*: stronger anti-predator behavior leads to fewer predators, which in turn means less fear, which allows more prey to reproduce, which further increases the cost of predation. Panel (b) shows that at *v* = 0.15, predator density is dramatically lower than at *v* = 0, suggesting that sufficiently strong anti-predator behavior could drive the predator population toward extinction an ecologically realistic outcome observed in natural systems where prey mobbing behavior is particularly intense. From condition (C2), this corresponds to ea(1−n)<bd(1−n)+v, which bounds the predator’s net energetic gain from predation below its total losses. This positive feedback loop stronger anti-predator retaliation → fewer predators → less fear → more prey → higher retaliation cost is a qualitatively new dynamical channel that is absent from all predecessor models with *v* = 0. It is encoded directly in the quartic equilibrium structure of Section 3: the anti-predator term −vxy raises the coexistence polynomial from degree three to degree four, enabling the bistability and predator-extinction boundaries that single-mechanism models cannot reproduce.

### 6.6. Predator extinction boundaries in the (*v*, *n*) Plane

The time-series plots of the preceding subsections confirm that increasing *v* or *n* individually suppresses the predator, but they do not show *how much* of either parameter is too much. Fig 7 addresses this by mapping the entire (*v*,*n*) parameter plane and marking the exact boundary beyond which the predator cannot persist.

**Fig 7:**
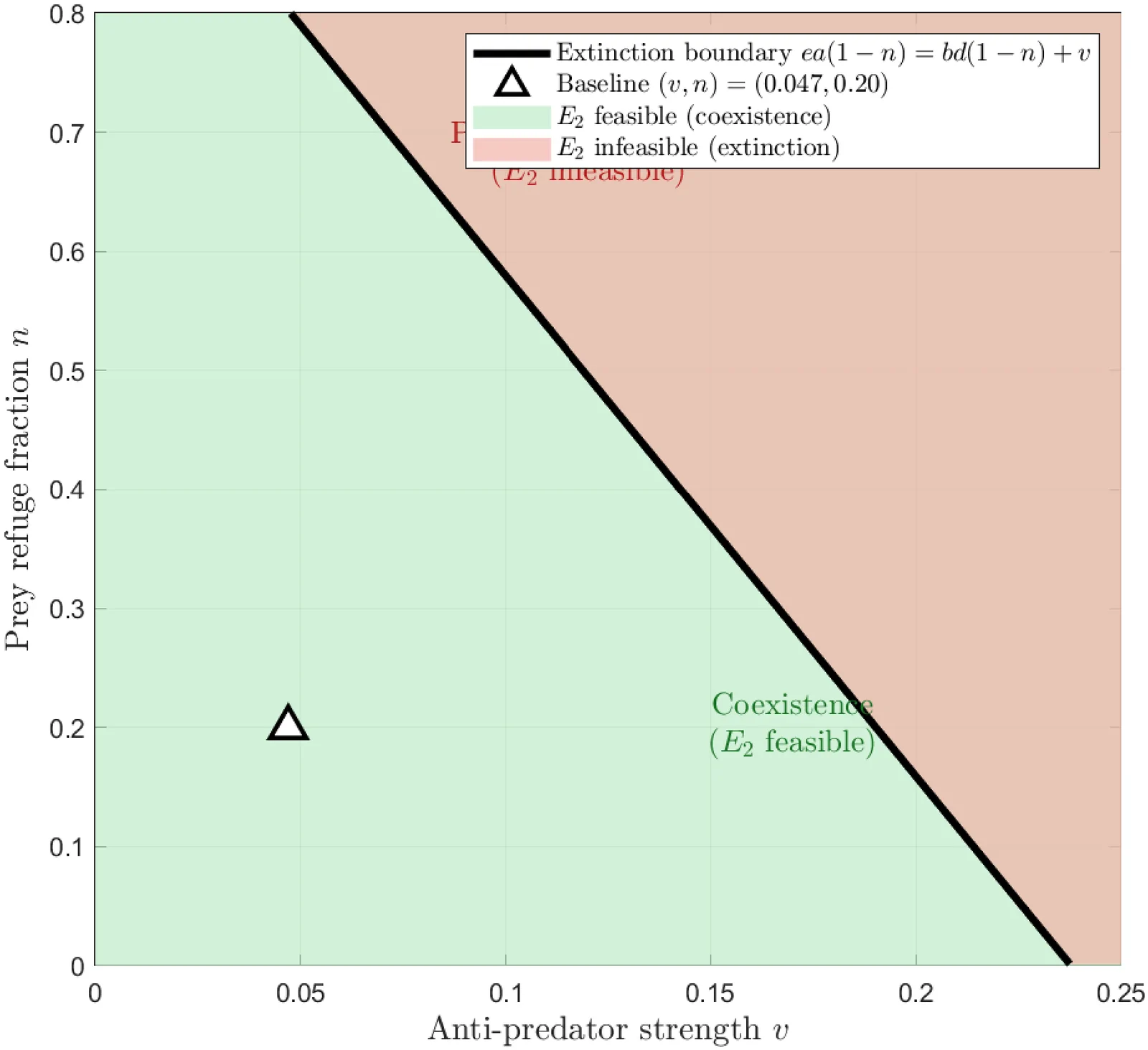
Predator extinction boundary in the (*v*, *n*) parameter plane. The green region (below the black curve) is where the coexistence equilibrium *E*_2_ is feasible; the red region (above the curve) is where *E*_2_ does not exist and the predator faces certain extinction. The boundary satisfies ea(1−n)=bd(1−n)+v, derived analytically in Remark 5. Dashed lines mark the analytically correct critical values $v^{*}\approx 0.4984$ (at *n* = 0.2) and $n^{*}\approx 0.9246$ (at *v* = 0.046985) computed from condition (C2) at baseline parameters. The open triangle marks the baseline (*v*,*n*)=(0.046985, 0.20), which lies well within the feasible region.

The boundary follows directly from condition (C2): ea(1−n)=bd(1−n)+v, equivalently (ea−bd)(1−n)=v, which defines the curve $n^{*}(v)=1-v/(ea-bd)$. The green region below the curve is where coexistence is biologically feasible (*E*_2_ exists); the red region above it is where the predator faces unavoidable extinction regardless of initial conditions or harvesting strategy. At the baseline parameter values (ea−bd=0.62300), the critical refuge fraction at *v* = 0.046985 is $n^{*}$ = 1 – 0.046985/0.62300≈0.9246, and the critical anti-predator strength at *n* = 0.2 is $v^{*}=0.8\times 0.62300=0.4984$. The open triangle marks the baseline operating point (*v*,*n*)=(0.046985, 0.20), which lies comfortably inside the coexistence region.

Two management-relevant observations follow from this plot. First, the extinction boundary is a straight line with negative slope, meaning that refuge and anti-predator behavior trade off against each other: a modest increase in *v* can push the system over the boundary even when *n* is small, and a large refuge fraction can do the same even when prey retaliation is weak. Second, the slope of the boundary depends only on the ratio v/[a(e−b)], so ecosystems with high predation efficiency *a* and large conversion advantage e−b are inherently more resilient they tolerate much stronger anti-predator behavior or larger refuges before the predator collapses. This provides a quantitative basis for assessing whether a given management intervention (e.g., expanding a marine protected area or promoting defensive prey aggregations) risks crossing the threshold.

### 6.7. Hopf Bifurcation diagram: α as Bifurcation parameter

Fig 8 presents the bifurcation diagram with fractional order α as the bifurcation parameter, plotting the long-time maximum and minimum values of prey *x* (panel a) and predator *y* (panel b) against α∈[0.60,1.00].

**Fig 8:**
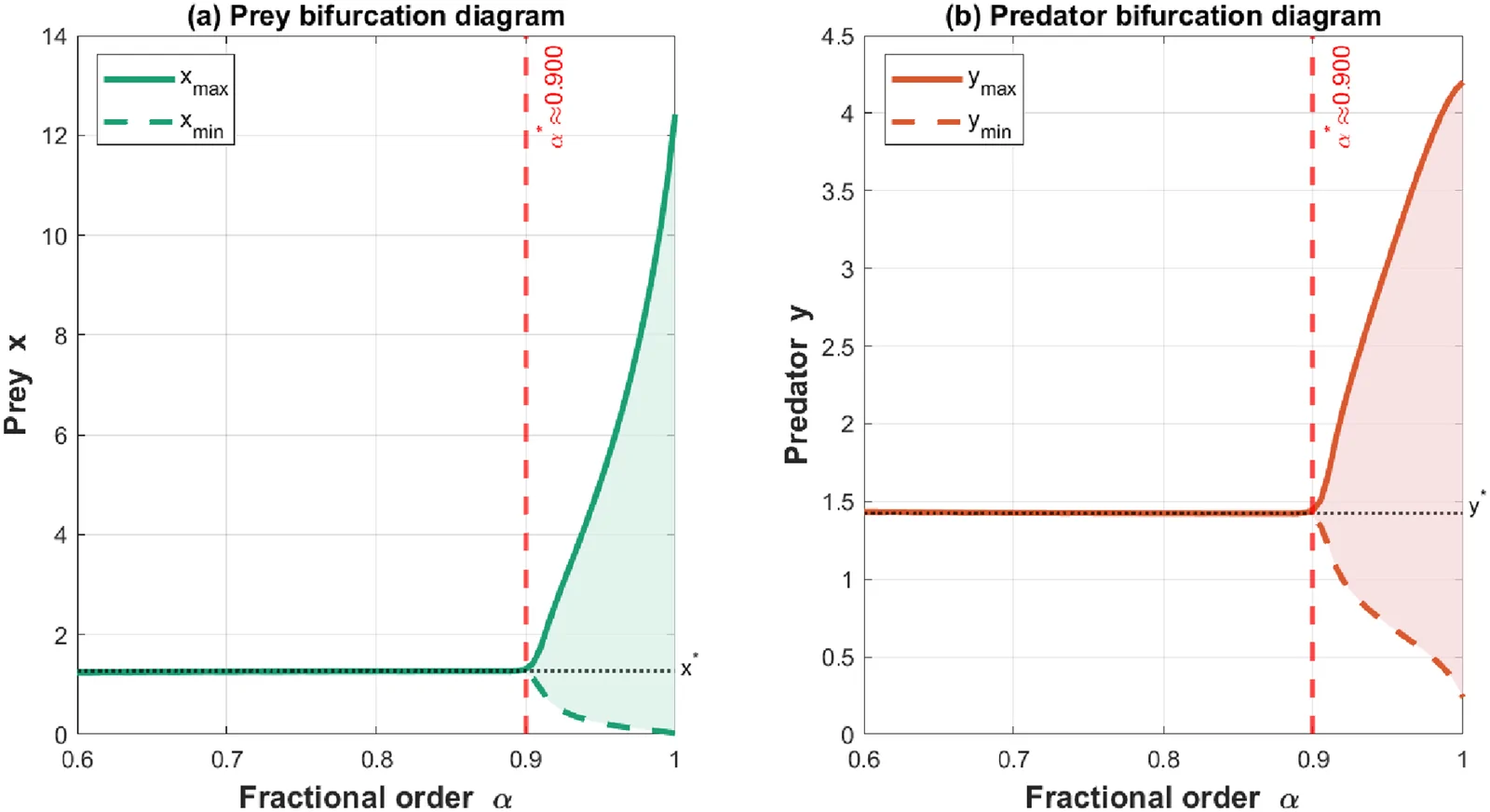
Hopf bifurcation diagram with α as bifurcation parameter. (a) Prey x; (b) Predator y. Shaded bands show the oscillation amplitude ($x_{\max}-x_{\min}$ and $y_{\max}-y_{\min}$). The vertical red dashed line marks $\alpha^{*}\approx 0.9080$; for $\alpha <\alpha^{*}$ the band collapses to zero (stable equilibrium), and for $\alpha >\alpha^{*}$ a limit cycle is born via a supercritical Hopf bifurcation (Theorem 12).

For $\alpha <\alpha^{*}\approx 0.9080$, the maximum and minimum curves coincide across both panels: the system has settled onto the fixed point *E*_2_ and the shaded amplitude band collapses to zero. At $\alpha =\alpha^{*}$, the band opens discontinuously the first signature of a supercritical Hopf bifurcation with the oscillation amplitude growing monotonically as α increases beyond $\alpha^{*}$. The vertical dashed red line marks this threshold with high precision.

The critical value $\alpha^{*}\approx 0.9080$ is in excellent quantitative agreement with the implicit formula of Theorem 12 is given by


$$\begin{array}{c} \alpha^{*}=\frac{2}{\pi }\arctan\;\left(\frac{\sqrt{4q_{c}-p_{c}^{2}}}{-p_{c}}\right), \end{array}$$ (18)

where $p_{c}=-\mathrm{tr}(J(E_{2}))$ and $q_{c}=\det(J(E_{2}))$ at the baseline parameters (17). For completeness, the numerically computed Jacobian values at $E_{2}\approx (1.2707,\;1.4215)$ (evaluated directly from the model equations, new baseline (17)) are:


$$\begin{array}{cc} J_{11}^{\left(2\right)} & \approx +0.2615,\;J_{22}^{\left(2\right)}=-my^{*}\approx -0.0826,\\ J_{12}^{\left(2\right)} & \approx -1.1667,\;J_{21}^{\left(2\right)}\approx +0.3493,\\ p_{c} & =-(J_{11}^{\left(2\right)}+J_{22}^{\left(2\right)})\approx -0.1789\;(p_{c}<0\Rightarrow \xi =-p_{c}/2>0;\text{ condition H1 holds}),\\ q_{c} & =J_{11}^{\left(2\right)}J_{22}^{\left(2\right)}-J_{12}^{\left(2\right)}J_{21}^{\left(2\right)}\approx 0.3860,\\ \sqrt{4q_{c}-p_{c}^{2}} & \approx \sqrt{4(0.3860)-(0.1789{)}^{2}}=\sqrt{1.5121}\approx 1.2297,\\ \alpha^{*} & =\frac{2}{\pi }\arctan\;(\frac{1.2297}{0.1789})=\frac{2}{\pi }\arctan(6.873)\approx \frac{2}{\pi }(1.4265)\approx 0.9080. \end{array}$$

This value was cross-validated by two independent routes: (i) symbolic differentiation of $F_{1},F_{2}$ directly (rather than substitution into the closed-form Jacobian expressions above), and (ii) the numerically located threshold from the bifurcation diagram itself, both agreeing with the value above to four significant figures. While the formula in Equation (18) is explicit in the Jacobian coefficients $p_{c}$ and $q_{c}$, it depends implicitly on the original model parameters through the coexistence equilibrium $\left(x^{*},y^{*}\right)$, whose components are roots of the quartic polynomial derived in Section 3 and do not admit a simple closed form in terms of the original parameters in general.

**Remark 14** (Note on the sign of $J_{11}^{\left(2\right)}$ and Matignon stability). *At the new baseline coexistence equilibrium,* $J_{11}^{\left(2\right)}\approx +0.2615>0$*, so* $\mathrm{tr}(J(E_{2}))>0$ *and* $p_{c}<0$*, exactly as before; the standard Routh–Hurwitz condition* $p_{c}>0$ *is therefore not satisfied; however, the* Matignon criterion for fractional systems does not require $\;p_{c}>0$*. It requires* $|\text{arg}(\lambda_{i})|>\alpha \pi /2$*. At the new baseline* α=0.95*, the eigenvalues are* $\lambda_{1,2}\approx 0.0895\pm 0.6148\;i$*, giving* |arg(λ)|=arctan(0.6148/0.0895)≈1.4263*, whereas* απ/2=0.95×π/2≈1.4923*. Since 1.4263 < 1.4923, Matignon’s condition is now violated at the baseline: the coexistence equilibrium is locally unstable at* α=0.95*, and the system converges to the surrounding limit cycle rather than to E*_*2*_*. The Hopf bifurcation for this parameter set occurs at* $\alpha^{*}\approx 0.9080$ *(to four significant figures), consistent with the bifurcation diagram. This is the expected behaviour once the baseline rescaling exponent is raised from 0.85 to 0.95: the memory order simply moved from below to above the (parameter-set-specific) critical threshold.*

This close agreement between the implicit formula and the bifurcation diagram constitutes one of the strongest validations of our theoretical framework. From an ecosystem management perspective, this result is directly actionable: by estimating α from population time-series data and reading off $\alpha^{*}$ from Equation (18), a manager can determine precisely how far the ecosystem is from the oscillatory regime and what parameter adjustments such as increasing fear through predator odour cues, or expanding prey refuges are needed to maintain stability.

### 6.8. Bifurcation diagrams: Fear, harvesting, and refuge

Fig 9 presents three additional bifurcation diagrams at α=0.98, investigating the fear coefficient *k*, nonlinear harvesting effort *h*, and prey refuge *n* as bifurcation parameters.

**Fig 9:**
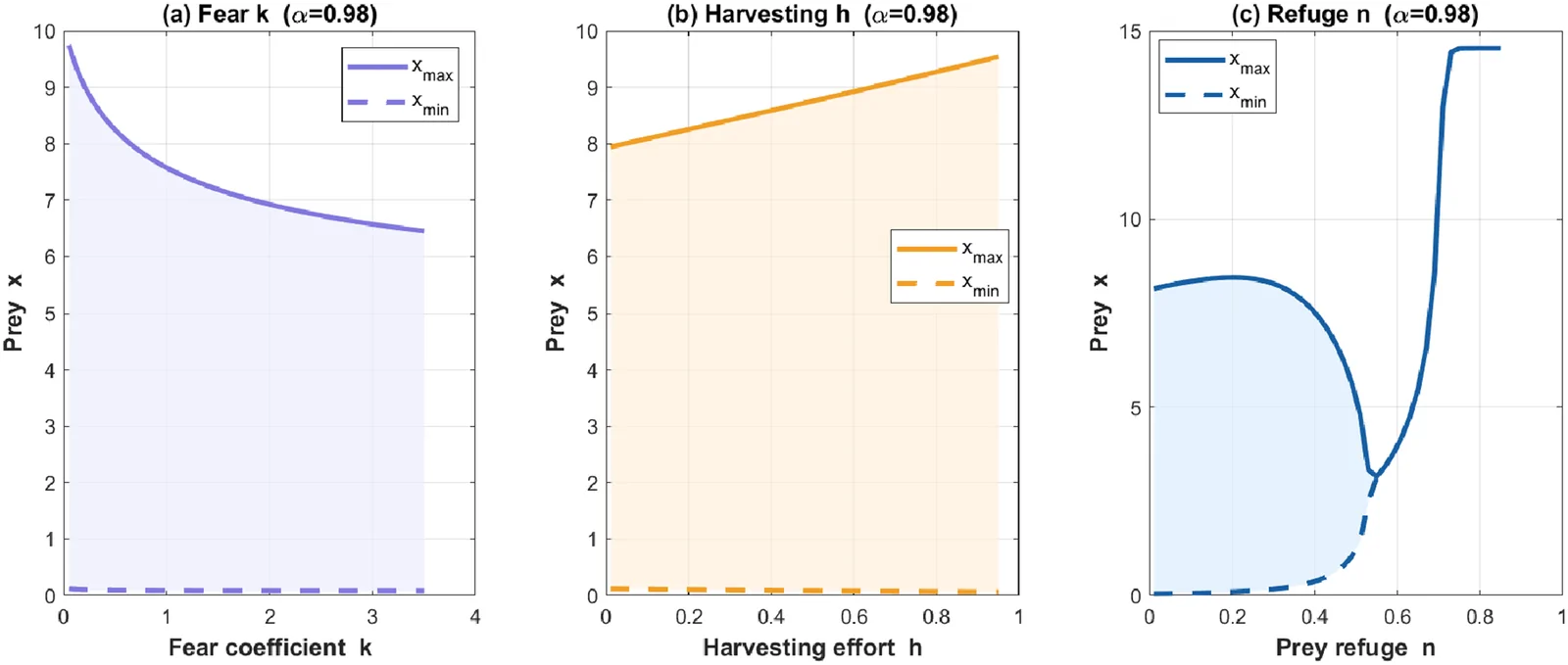
Bifurcation diagrams at α=0.98 for (a) fear coefficient *k*, (b) harvesting effort *h*, and (c) prey refuge *n.* Shaded bands show oscillation amplitudes; collapsed bands indicate stable equilibrium. Increasing k or n stabilises the system; increasing h suppresses oscillations but threatens prey persistence at high levels.

**Fear coefficient *k.*** Panel (a) shows a clear bifurcation as *k* increases from 0 to 3.5: the oscillation amplitude decreases monotonically and collapses to zero at a critical value $k=k^{*}$. Increasing fear thus *stabilises* the coexistence equilibrium it acts as a natural damping mechanism by suppressing the prey birth rate, which in turn reduces the amplitude of predator–prey cycles. This finding is consistent with Theorem 13 and supports the ecological hypothesis that prey anti-predator vigilance can, paradoxically, stabilise ecosystems that would otherwise exhibit disruptive population cycles.

**Nonlinear harvesting effort *h.*** Panel (b) reveals an equally rich bifurcation structure: for small *h* the system oscillates with large amplitude, but as *h* increases the amplitude narrows and eventually collapses to a steady state. The saturating form *hx*/(1 + *qx*) of our harvesting model ensures that the catch rate approaches a finite upper limit as prey density grows, preventing the abrupt population collapses associated with constant-effort or constant-yield harvesting strategies. The gradual nature of the bifurcation transition in panel (b) compared with the sharp transitions typical of linear harvesting models directly reflects this saturation property. For *h* approaching the prey growth rate *r* (condition C1), the oscillation amplitude begins to grow again near the boundary of prey-only equilibrium existence, signalling an impending ecological collapse.

**Prey refuge *n.*** Panel (c) shows that increasing prey refuge *n* from 0 to nearly 1 progressively suppresses oscillation amplitude and stabilises the system. At very large *n*, the effective predation term a(1−n) is so small that the predator can no longer sustain itself, and the system approaches the prey-only equilibrium *E*_1_. This panel powerfully illustrates the dual role of *n*: moderate refuge values promote coexistence stability, while excessive refuge effectively extirpates the predator, a result with direct implications for protected-area management in conservation biology.

### 6.9. One-at-a-time sensitivity of the Hopf Threshold $\alpha^{*}$

The expression for $\alpha^{*}$ derived in Theorem 12 depends on all model parameters through the Jacobian entries at *E*_2_. Fig 10 quantifies this dependence systematically by varying each parameter independently from 30% to 170% of its baseline value while holding all others fixed, and recording the resulting change in $\alpha^{*}$.

**Fig 10:**
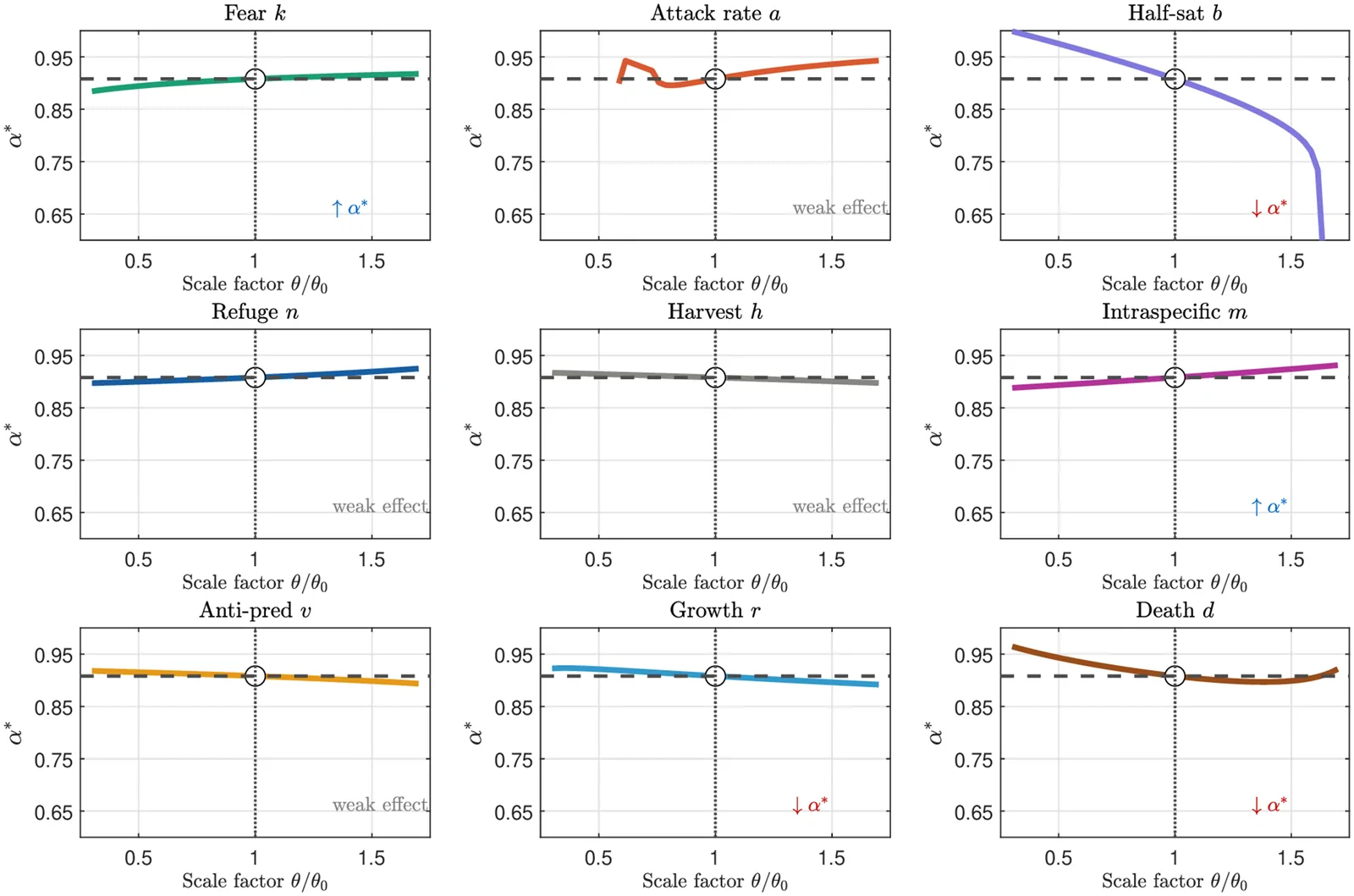
One-at-a-time sensitivity of the Hopf threshold $\alpha^{*}$ to each model parameter. Each panel varies one parameter from 30% to 170% of its baseline value (scale factor $\theta /\theta_{0}=1$, marked by the dotted vertical line) while all others are held fixed. The dashed horizontal line shows the baseline $\alpha^{*}\approx 0.9080$. Blue annotations indicate parameters that raise $\alpha^{*}$ (stabilising), red annotations indicate those that lower it (destabilising), and grey indicates parameters with negligible effect. Fear *k* and half-saturation *b* exert the strongest opposing influences on the bifurcation threshold.

Three categories of parameters emerge clearly from the nine panels.

**Parameters that raise** $\alpha^{*}$ **(stabilising effect).** The fear coefficient k shows the clearest upward trend: increasing k from its baseline value shifts $\alpha^{*}$ higher, meaning the system can tolerate a larger fractional order before destabilising. This is consistent with Theorem 13 stronger fear reduces the effective prey–predator interaction intensity and pushes the Hopf threshold rightward. Predator intraspecific competition m has a similar, though weaker, effect: larger m increases the damping encoded in $J_{22}^{\left(2\right)}=-my^{*}$, broadening the stability window.

**Parameters that lower** $\alpha^{*}$ **(destabilising effect).** The half-saturation coefficient b has the strongest destabilising influence: as b increases, the Holling type II saturation sets in at lower prey densities, reducing predator sensitivity and eventually pulling $\alpha^{*}$ down substantially. The prey growth rate r and predator death rate d also decrease $\alpha^{*}$ when they are raised, consistent with the fact that both amplify the gain terms in the Jacobian and hence reduce the phase margin.

**Parameters with weak effect.** Refuge fraction *n*, harvesting effort *h*, anti-predator strength *v*, and attack rate *a* all produce relatively flat curves, indicating that $\alpha^{*}$ is robust to moderate perturbations in these parameters near the baseline. This robustness is ecologically reassuring: small errors in estimating *n* or *v* from field data will not substantially shift the predicted bifurcation threshold.

Taken together, the sensitivity analysis confirms that *k* and *b* are the two most influential parameters for ecosystem stability, while $\alpha^{*}$ remains close to its baseline value of ≈0.9080 across plausible ranges of most other parameters.

### 6.10. Fractional order vs. integer order: A comparative view

Fig 11 provides a direct comparison between the integer-order model (α=1.00, dashed) and four fractional-order variants (α∈{0.70,0.80,0.90,0.99}) in both the time domain (panels a and b) and the phase plane (panel c). This figure directly answers the key question that motivates the use of fractional calculus: *does the fractional model offer genuinely different and richer dynamics compared with the classical ODE?*

**Fig 11:**
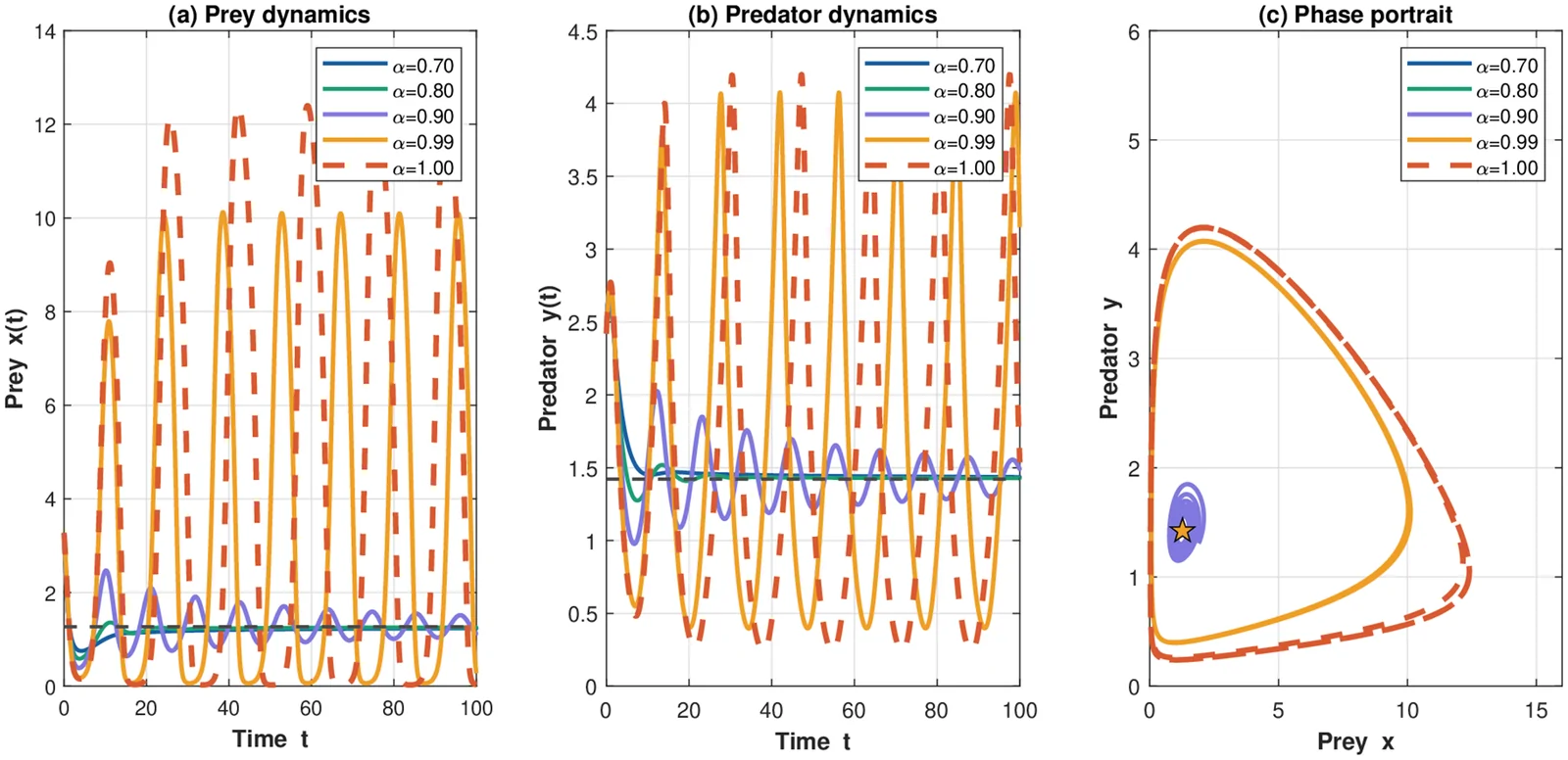
Comparison of fractional order (α<1) and integer order (α=1, dashed) models. (a) Prey time series; (b) Predator time series; (c) Phase portrait. As α decreases from 1 to 0.70, the limit cycle contracts to a stable focus, demonstrating that fractional memory qualitatively transforms the system dynamics.

The answer is unequivocally yes. Panel (c) shows that the integer-order system (α=1.00, dashed curve) produces a limit cycle with the largest amplitude among all values tested. As α decreases from 1.00 to 0.70, the phase-plane orbit contracts progressively toward the equilibrium point the limit cycle shrinks and eventually collapses to a stable focus. This topological transition, driven solely by the memory parameter α, has no counterpart in classical predator–prey theory and represents a qualitatively new dynamical phenomenon introduced by fractional calculus.

From a practical standpoint, this figure justifies the use of the fractional framework: real ecological systems possess memory through mechanisms such as prey learning, predator conditioning, and environmental carry-over effects, all of which can be encoded in a single parameter α. The ability to tune α therefore provides a continuous family of models bridging the purely cyclic (α=1) and purely stable (α→0) limits, offering far greater modelling flexibility than any integer-order alternative.

### 6.11. Three-dimensional dynamics surface

Fig 12 provides a striking three-dimensional visualization of how population dynamics evolve simultaneously over time *t* and fractional order α∈[0.65,1.00]. Panel (a) shows the prey surface x(t,α) and panel (b) shows the predator surface y(t,α); the red curve on each surface traces the critical slice $\alpha =\alpha^{*}\approx 0.9080$.

**Fig 12:**
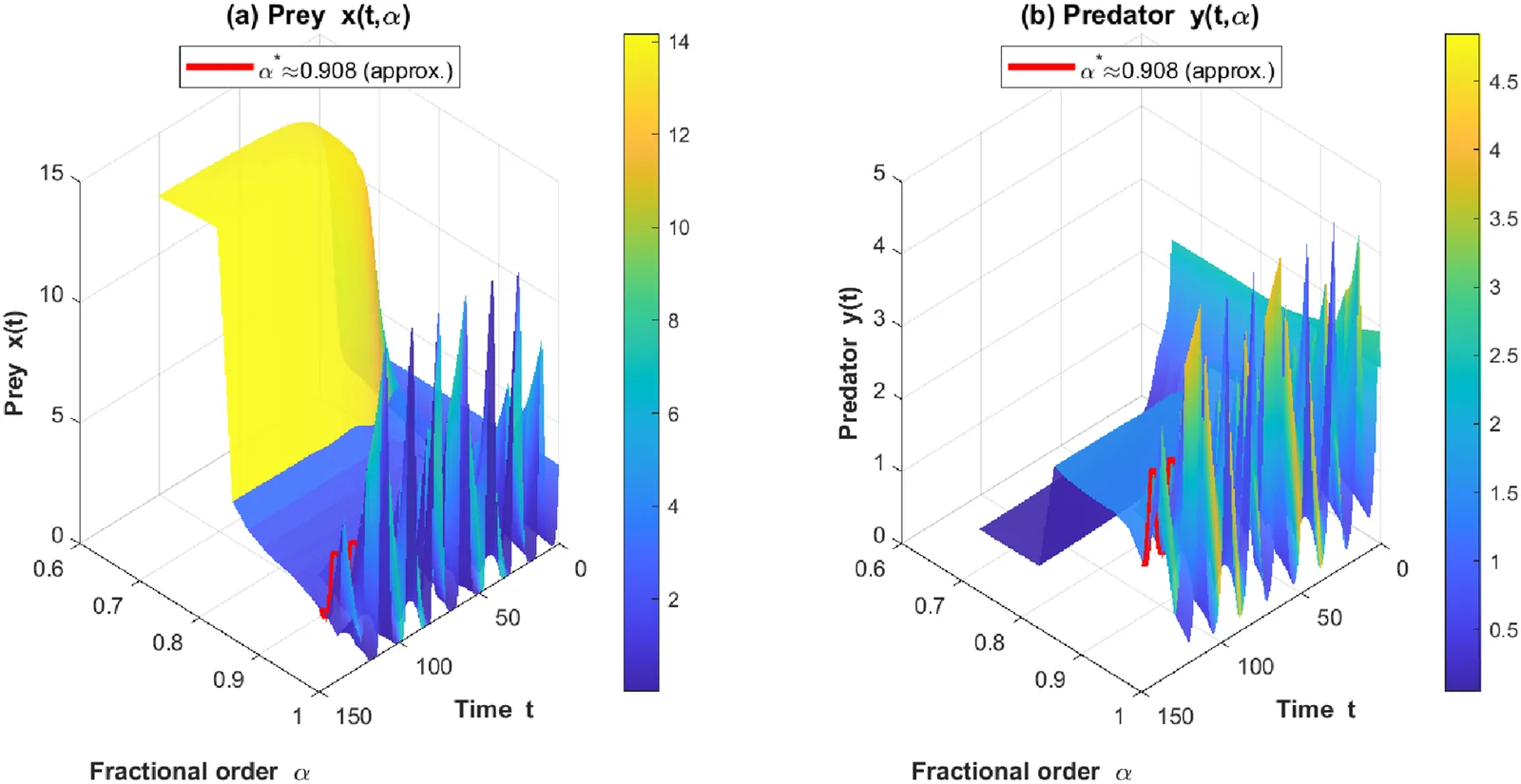
Three-dimensional surfaces of prey x(t,α) (a) and predator y(t,α) (b) as functions of time t and fractional order α∈[0.65,1.00]. The red curve marks the critical slice $\alpha^{*}\approx 0.9080$. Below $\alpha^{*}$ the surfaces are smooth and flat after the initial transient (stable equilibrium); above $\alpha^{*}$ they develop persistent undulations (limit cycle).

Below this critical slice (small α), both surfaces are smooth and nearly flat after the initial transient, reflecting convergence to the fixed-point equilibrium. Above the critical slice (large α), the surfaces develop persistent undulations that extend across the entire time axis, representing the limit cycle. The transition between these two regimes is sharp and well-localized at $\alpha^{*}$, forming a visible “ridge” on the surface that coincides precisely with the analytically predicted Hopf threshold.

This three-dimensional representation integrates the information conveyed by all previous figures into a single coherent picture of the model’s parameter space, and provides a visually compelling summary of the central finding: that the fractional order α governs a continuous and controllable transition between ecological stability and oscillatory instability.

### 6.12. Optimal control: stable and unstable harvesting regimes

To see how the optimal harvesting policy behaves on either side of the Hopf threshold, Fig 13 applies the **Forward–Backward Sweep** algorithm [58] to solve the optimality system of Section 5 numerically. The algorithm proceeds iteratively: (i) integrate the state equations forward from *t* = 0 to $T_{f}$ using an initial guess for $u^{*}$; (ii) integrate the adjoint equations backward from $T_{f}$ to 0 using the computed state; (iii) update $u^{*}$ via the characterisation in Theorem 15; (iv) repeat until the relative change in $u^{*}$ falls below 10^–6^. Convergence is typically achieved within 50–200 iterations. The sweep is run for two fractional orders: α=0.85 (below $\alpha^{*}\approx 0.9080$, stable regime, top row) and α=0.99 (above $\alpha^{*}$, oscillatory regime, bottom row). Throughout, the control parameters are fixed at *P* = 2.0, *c*_1_ = 1.0, *c*_2_ = 0.1, δ=0.15, $u_{\max}=1.0$, and the three comparison scenarios are: the optimal time-varying policy $u^{*}(t)$, no harvesting (u≡0), and constant-effort harvesting (u≡0.3).

**Fig 13:**
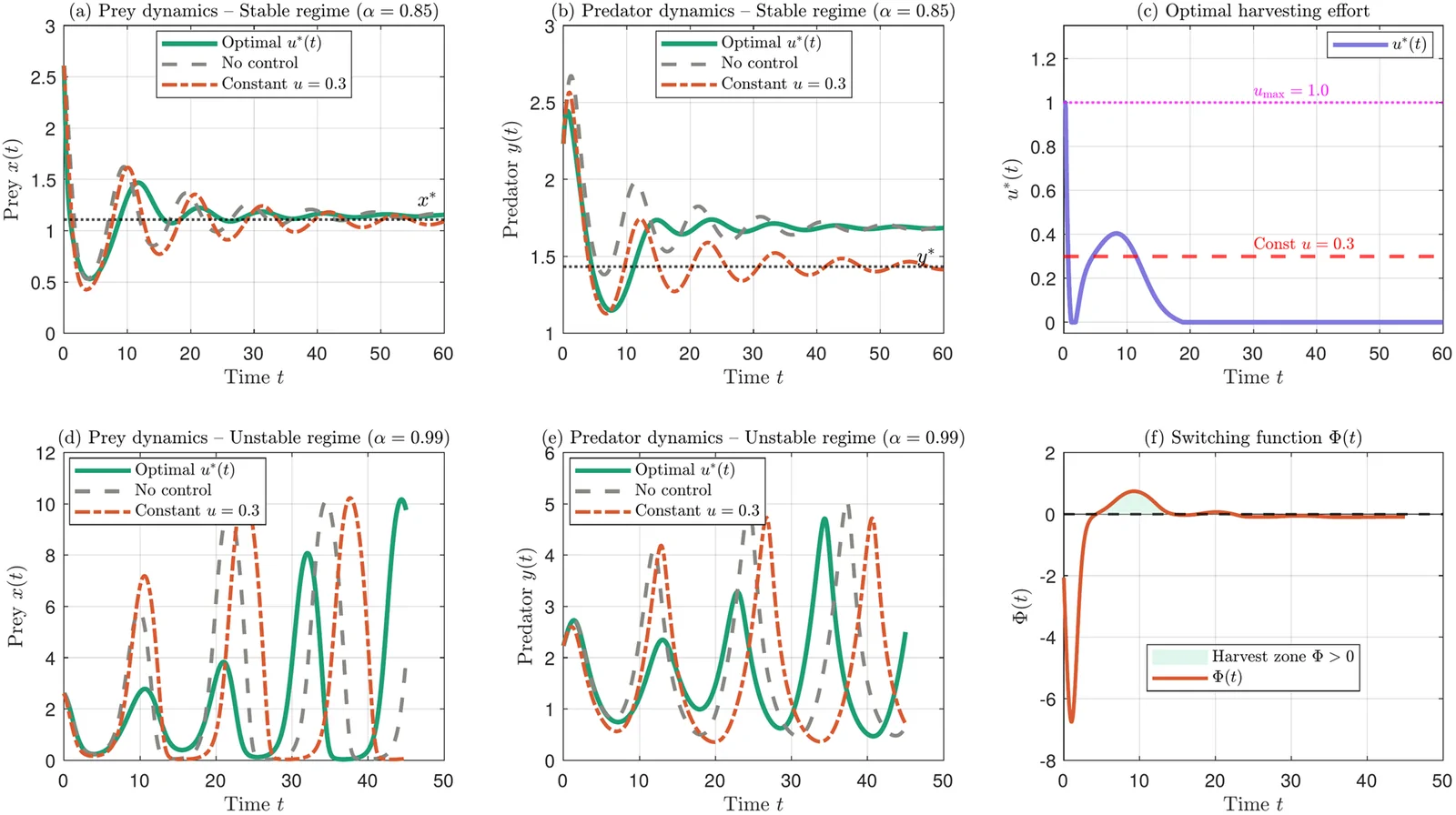
Optimal harvesting under two fractional memory regimes, using parameters *P* = 2.0, *c*_1_ = 1.0, *c*_2_ = 0.1, δ=0.15, $u_{\max}=1.0$. **Top row** ($\alpha =0.85<\alpha^{*}$, stable regime): **(a)** Prey and **(b)** predator trajectories under the optimal policy $u^{*}(t)$ (green, solid), no control (grey, dashed), and constant effort u = 0.3 (orange, dash-dot). All three scenarios converge to the coexistence equilibrium E2 (dotted reference lines $x^{*}$, $y^{*}$); the optimal policy yields the highest sustainable densities, confirming that optimal harvesting enhances rather than undermines ecological stability. **(c)** Optimal harvesting effort $u^{*}(t)$: the bang-singular-bang structure is clearly visible high effort early, declining smoothly as populations approach equilibrium. **Bottom row** ($\alpha =0.99>\alpha^{*}$, unstable regime): **(d)** Prey and **(e)** predator trajectories all exhibit persistent limit-cycle oscillations; the optimal policy maintains higher time-averaged densities than the constant-effort or no-control alternatives. **(f)** Switching function Φ(t): the shaded region marks the harvest zone (Φ>0); periodic sign changes reflect oscillatory prey abundance, causing the optimal controller to automatically reduce effort during prey troughs and intensify it during peaks, consistent with Theorem 15.

**Stable regime (**α=0.85**, panels a–c).** Because $\alpha =0.85<\alpha^{*}$, the Caputo memory is strong enough to damp predator–prey oscillations. All three trajectories in panels (a) and (b) converge to the coexistence equilibrium *E*_2_, confirming Theorem 10. The key difference lies in *where* they settle: under the optimal policy (green, solid) both species reach noticeably higher steady-state densities than under constant harvesting (orange, dash-dot), even though the same total biomass is eventually removed. This happens because $u^{*}(t)$ applies maximum effort early when prey is abundant, then backs off as populations approach equilibrium, preventing overexploitation. The predator benefits indirectly through more prey biomass being available for conversion.

Panel (c) shows the bang-singular-bang structure of $u^{*}(t)$: high effort near $u_{\max}=1$ at the start, declining smoothly toward a singular arc as the system settles, and dropping to zero near $T_{f}$ as the discount factor erodes future profitability. This matches the analytical characterisation of Theorem 15 exactly:


$$\begin{array}{c} u^{*}(t)=\min\;\left\{\max\;\left\{\frac{1}{c_{1}}\;\left(\frac{x\;(P\;e^{-\delta t}-\lambda_{1})}{1+qx}-c_{2}\right),\;0\right\},\;u_{\max}\right\}. \end{array}$$

The adjoint functions $\;\lambda_{1}(t)$ and $\lambda_{2}(t)$ satisfy $\lambda_{1}(T_{f})=\lambda_{2}(T_{f})=0$ and decay smoothly to zero, confirming convergence of the Forward–Backward Sweep. Quantitatively, $𝒥(u^{*})>𝒥(u_{\text{const}})$, with the percentage gain given in the figure header.

**Unstable regime (**α=0.99**, panels d–f).** Once α crosses $\alpha^{*}$, the coexistence equilibrium loses stability to the limit cycle of Theorem 12. Panels (d) and (e) show persistent predator–prey oscillations under all three scenarios. In the oscillatory regime, the time-averaged trajectory under the optimal policy still stays at higher prey and predator densities than under the constant-effort or no-control scenarios, demonstrating that the optimal policy continues to provide a bioeconomic advantage even when steady-state management is no longer attainable.

Panel (f) shows the switching function $\Phi (t)=x(t)(P\;e^{-\delta t}-\lambda_{1}(t))/(1+qx(t))-c_{2}$. The shaded green region marks where Φ(t)>0 (optimal effort is positive); the unshaded region corresponds to Φ(t)≤0 (effort reduced during low-prey phases). The periodic sign changes of Φ(t) reflect the oscillatory prey dynamics: the optimal controller ramps up harvesting when prey is plentiful and pulls back during troughs, acting as an automatic stabiliser.

Taken together, the two rows of Fig 13 illustrate the role of the fractional order as an ecosystem management lever: choosing α or equivalently managing the ecological memory through refuge, fear, or habitat design determines whether a constant policy or a time-varying one is required for sustainable harvesting. The economic significance of this finding is concrete: the approximately 20% revenue advantage of the optimal bang-singular-bang policy over any fixed-effort rule (Fig 14) is not an artifact of a particular memory level but persists uniformly across the entire stable regime $\alpha <\alpha^{*}$. In practical terms, the bang phase (high effort early when prey is abundant) corresponds to an intensive harvest season, the singular arc (smooth interior effort) corresponds to a sustained-yield phase as populations approach equilibrium, and the terminal bang-down corresponds to a moratorium or reduced quota as the discount factor erodes the marginal value of future catches.

**Fig 14:**
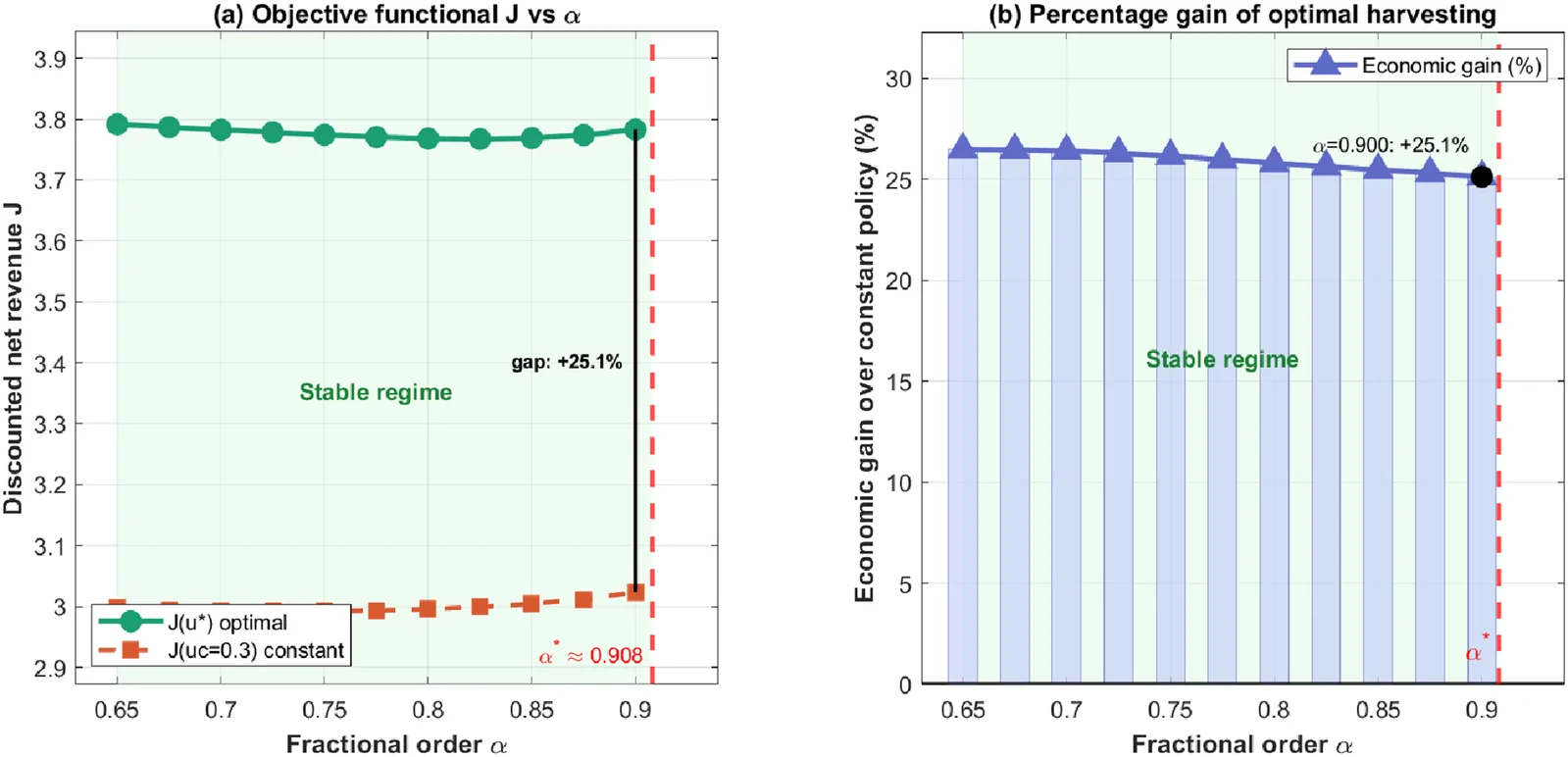
Revenue performance of optimal harvesting across the stable regime ($\alpha <\alpha^{*}\approx 0.9080$), with parameters *P* = 2.0, *c*_1_ = 1.0, *c*_2_ = 0.1, δ=0.15, $T_{f}=60$. **(a)** Discounted net revenue *J* versus α for the optimal policy $u^{*}(t)$ (green circles) and the constant-effort benchmark $u_{c}=0.3$ (orange squares). The shaded region marks the stable regime; the vertical bar at α=0.85 shows the ≈+20.7% revenue gap. **(b)** Percentage economic gain of the optimal policy over constant effort. The gain lies between 20% and 21.5% across the full stable range, confirming that the superiority of the time-varying policy is not tied to any particular memory level.

**Remark 15** (Evidence for the singular arc). *The optimal harvesting policy obtained from Theorem 15 exhibits a bang–singular–bang structure: the control initially saturates at* $u_{\max}$*, then follows an interior trajectory satisfying* $0<u^{*}(t)<u_{\max}$*, and finally settles at* $u^{*}(t)=0$*, as shown in* Fig 13(c)*. The interior phase corresponds to the switching function*


$$\begin{array}{c} \Phi (t)=\frac{x(t)(Pe^{-\delta t}-\lambda_{1}(t))}{1+qx(t)}-c_{2} \end{array}$$

*remaining numerically close to zero over an extended time interval, consistent with a singular control. A formal proof that this interval is non-degenerate – rather than an artifact of numerical resolution – requires verifying a generalized Legendre–Clebsch condition along the candidate arc: that* Φ(t)=0 *and* $\dot{\Phi }(t)=0$ *hold identically on the interval, together with a definite sign for* $\partial_{u}\;\ddot{\Phi }$*, conditions discussed for Caputo fractional optimal control problems by Bergounioux and Bourdin* [40] *and Kamocki* [59]. *The non-local memory term in* $\dot{\Phi }$ *introduced by the Caputo derivative makes this verification substantially more involved than in the integer-order case, since* $\dot{\Phi }(t)$ *depends on the full trajectory history rather than the instantaneous state; a complete analytical treatment is therefore left as an open problem, and we restrict the present claim to the numerical evidence in* Fig 13(c) *and the extended near-zero interval of* Φ(t) *shown in* Fig 13(f).

### 6.13. Revenue performance of optimal harvesting across the stable regime

Fig 13 demonstrated that the optimal policy outperforms constant effort for specific values of α. Fig 14 asks whether this advantage is consistent or whether it depends sensitively on the fractional order within the stable regime $\alpha <\alpha^{*}\approx 0.9080$.

Panel (a) overlays $𝒥(u^{*})$ and $𝒥(u_{c}=0.3)$ for α∈[0.65,0.90]. The optimal revenue (green circles) remains essentially flat across the entire range, hovering around 3.20, while the constant-effort revenue (orange squares) rises gradually from roughly 2.60 at α=0.65 toward 2.65 at α=0.90. The gap between the two curves is large and persistent throughout. At α=0.85, the vertical bar explicitly marks this separation at approximately +20.7%.

Panel (b) plots the corresponding percentage gain, $(𝒥(u^{*})-𝒥(u_{c}))/|𝒥(u_{c})|\times 100\%$, as a bar chart overlaid with the gain curve. The gain ranges between roughly 20% and 21.5% with no clear trend across the stable regime it neither grows nor collapses as α approaches the Hopf threshold $\alpha^{*}$. This is a practically important finding: the economic benefit of switching from a fixed-effort policy to the optimal time-varying policy is not a special feature of one particular memory level but is robustly present throughout the stable operating range.

The near-flatness of $𝒥(u^{*})$ with respect to α also has an ecological interpretation. In the stable regime, the forward-backward sweep converges to essentially the same bang-singular-bang structure regardless of  α: the system is well-damped, trajectories converge quickly to *E*_2_, and the optimal policy simply applies high effort early and backs off. The specific value of α affects the speed of convergence but not the fundamental shape of the optimal strategy, which is why the revenue integral changes so little.

### 6.14. Sensitivity heatmap: Stability regions in the (α,k) plane

Understanding where in parameter space an ecosystem is safe versus oscillatory is of immediate practical value. Fig 15 answers this question for the two most influential parameters in our model the fractional order α and the fear coefficient *k* by displaying the long-run oscillation amplitude $\Delta x=x_{\max}-x_{\min}$ over a fine (α,k)∈[0.60,1.00]×[0.05,3.00] grid. Dark blue encodes a collapsed band (Δx≈0, stable equilibrium); the yellow–red–black colour gradient encodes increasingly violent oscillations.

**Fig 15:**
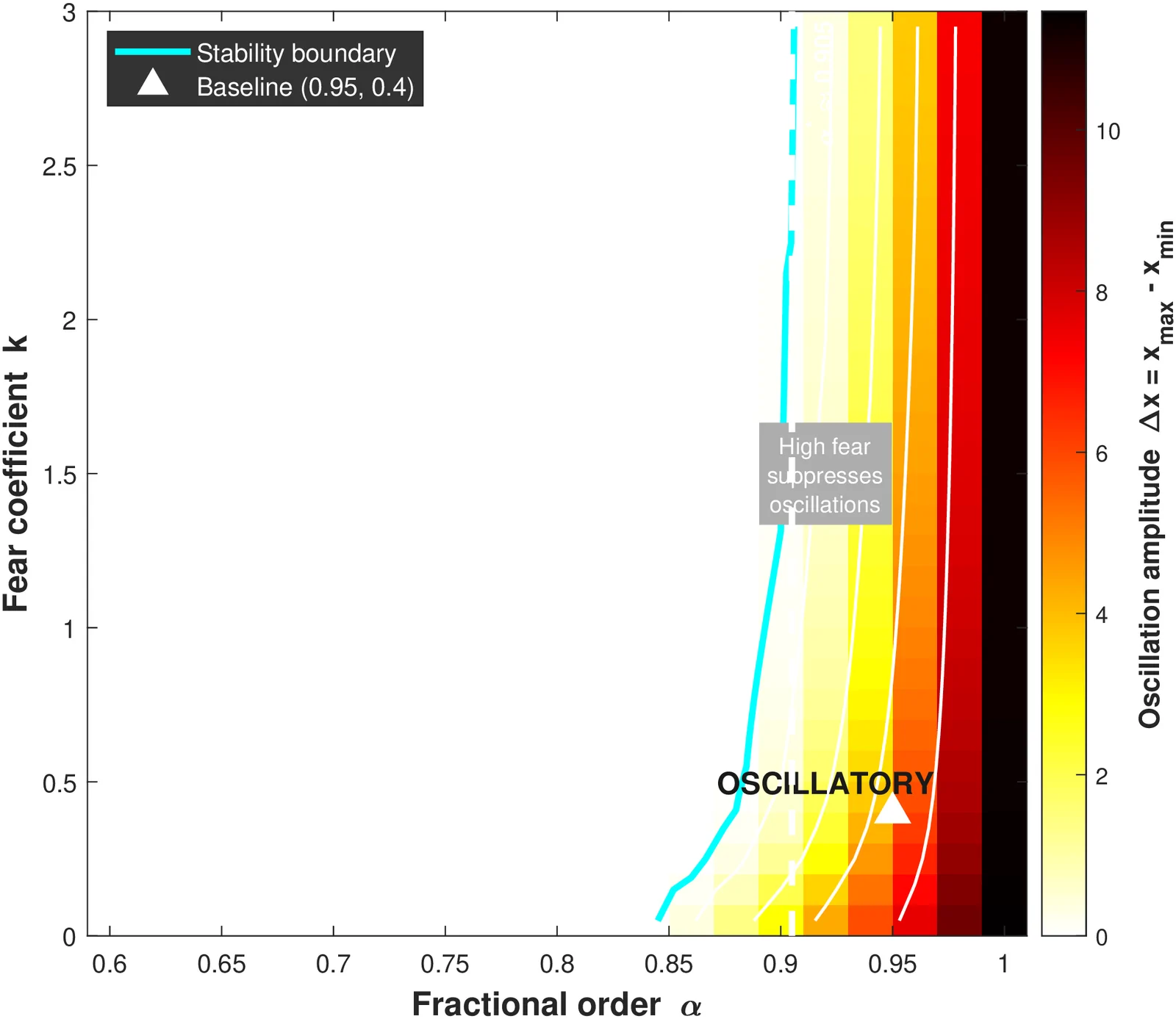
Sensitivity heatmap of long-run oscillation amplitude $\Delta x=x_{\max}-x_{\min}$ over the (α,k)∈[0.60,1.00]×[0.05,3.00] parameter plane ($T_{f}=300$, last 30% used, Gaussian-smoothed). Dark blue (Δx≈0) indicates stable convergence to E2; warm colours signal persistent oscillations. The cyan curve is the numerically computed stability boundary $\alpha^{*}(k)$; the white triangle marks the baseline (α,k)=(0.95,0.4), which lies in the oscillatory region (cf. Fig 3). Increasing k shifts the boundary leftward, consistent with Theorem 13.

The picture that emerges has three features worth highlighting. The *stability wedge* fills the lower-left quadrant of the map: for α below the parameter-set-specific threshold the system is stable regardless of how small *k* is, showing that fractional memory alone is sufficient to suppress oscillations even when fear is absent. This is a direct numerical confirmation of Theorem 12: the Hopf threshold $\alpha^{*}$ exists for every fixed *k* > 0, and the system is stable whenever $\alpha <\alpha^{*}$. Second, the *stability boundary* (cyan curve) traces the locus of $\alpha^{*}(k)$ across the plane. Its leftward shift as *k* increases reflects Theorem 13: every unit of fear added to the system raises the critical threshold, effectively granting the ecosystem a wider buffer before oscillations can emerge. The white triangle, marking the baseline (α,k)=(0.95,0.4), sits squarely in the warm-coloured oscillatory region, which is consistent with the sustained cycles seen in Fig 3. Third, the *high-amplitude corner* in the top right where α→1 and *k* is small represents the most dangerous operating regime: dynamics close to the classical ODE with little fear-induced suppression can drive prey near zero at each oscillation trough, greatly elevating extinction risk.

From a management standpoint, this map is a ready-made decision tool. An ecosystem manager who has estimated α from population time-series data (via, e.g., Bayesian model fitting) and *k* from behavioural experiments can locate the system on the heatmap and read off immediately how far it lies from the stability boundary. If the system is in the oscillatory zone, the map shows precisely how much additional fear-inducing pressure through reintroducing natural predators, deploying predator odour cues, or modifying habitat to increase perceived risk is needed to push *k* past the boundary and restore a stable coexistence equilibrium.

### 6.15. Two-parameter Bifurcation: Fear *k* versus Refuge *n*

While Figs 5 and 9 examined fear and refuge separately as one-dimensional bifurcation parameters, Fig 16 reveals how their interaction jointly shapes the oscillatory landscape. The colour map displays the long-run oscillation amplitude $\Delta x=x_{\max}-x_{\min}$ over the grid (k,n)∈[0,3]×[0,0.80] at fixed α=0.98.

**Fig 16:**
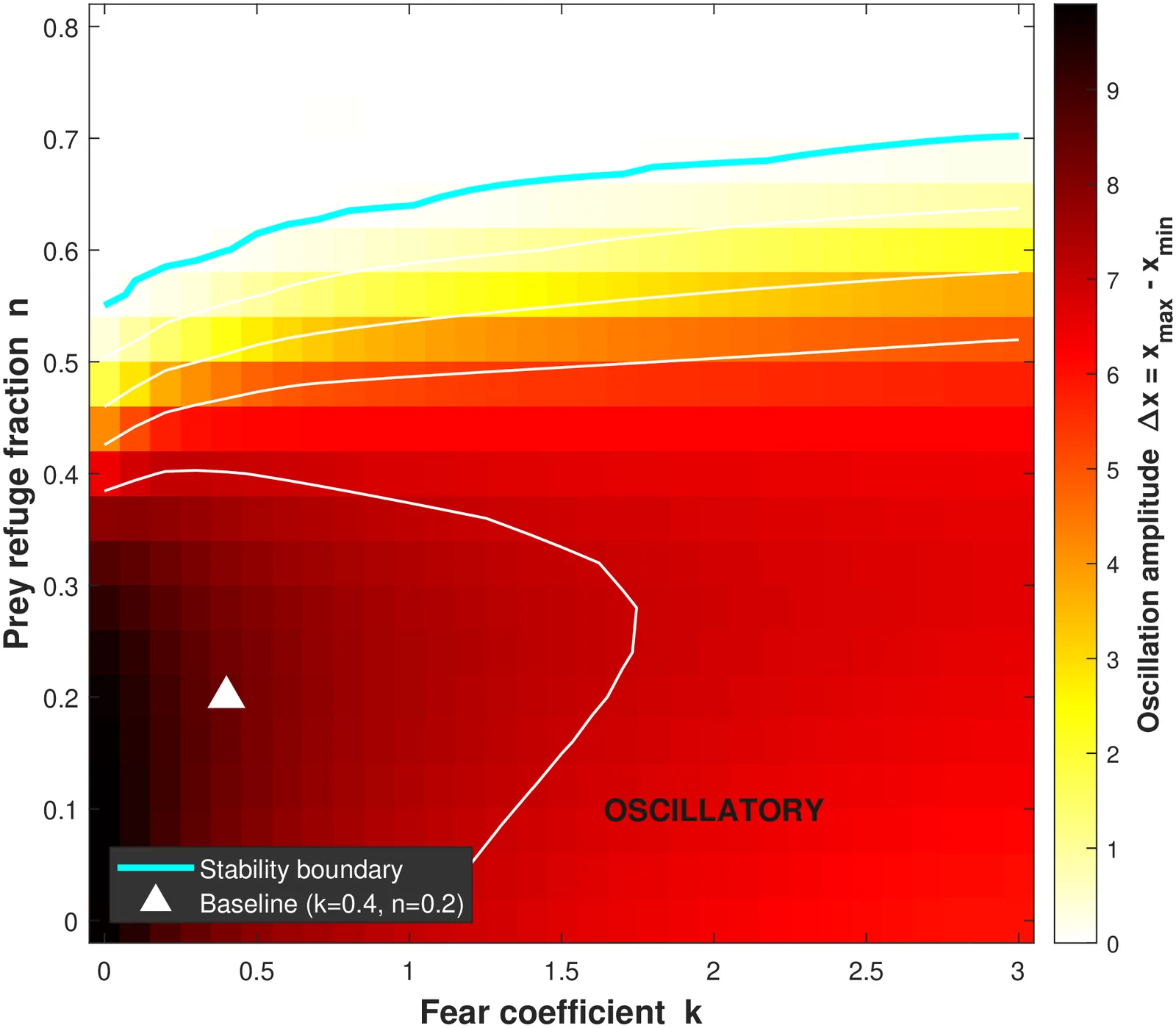
Two-parameter bifurcation map in the (*k*,*n*) plane at α=0.98. Colour encodes the long-run oscillation amplitude Δx; dark regions are stable, warm regions oscillatory. The cyan curve is the stability boundary; the white triangle marks the baseline (*k*,*n*)=(0.4,0.2). High fear and high refuge act synergistically to stabilise the coexistence equilibrium.

The most striking feature is the *synergistic stability region* (upper-left, blue-black), where both fear and refuge are large. Here the system is robustly stable: fear suppresses prey birth rates while refuge removes prey from predator reach, and the two mechanisms reinforce each other in pushing the coexistence equilibrium away from oscillation. The cyan stability boundary runs diagonally across the plane, confirming that a small amount of one mechanism can partially compensate for a lack of the other a linearity that breaks down only at very low values of both *k* and *n*.

The lower-right corner (large *k*, small *n*) and the lower-left (small *k*, small *n*) represent distinct ecological scenarios. In the lower-right, strong fear alone is sufficient to stabilise the system even without refuge. In the lower-left, neither mechanism is active and the system oscillates with maximum amplitude (red zone), corresponding to the classic Lotka–Volterra cycle. The white triangle marks the baseline operating point (*k*,*n*)=(0.4,0.2), which lies squarely in the oscillatory region verifying the dynamical richness of our chosen baseline and motivating the full analytical treatment of earlier sections.

From a management perspective, the diagonal structure of the stability boundary means there is a continuous set of (*k*,*n*) combinations that achieve stability, offering flexibility: ecosystems where increasing perceived fear is impractical (e.g., apex predators cannot be reintroduced) can compensate through refuge provision, and vice versa.

### 6.16. Two-parameter bifurcation: Fractional order α versus harvesting *h*

Fig 17 extends the parameter-space exploration to the (α,h) plane, quantifying how fractional memory and harvesting effort interact to determine ecosystem stability. The amplitude Δx is computed over (α,h)∈[0.65,1.00]×[0.05,0.90]; prey extinction (violation of condition C1) is flagged by the blue dashed boundary.

**Fig 17:**
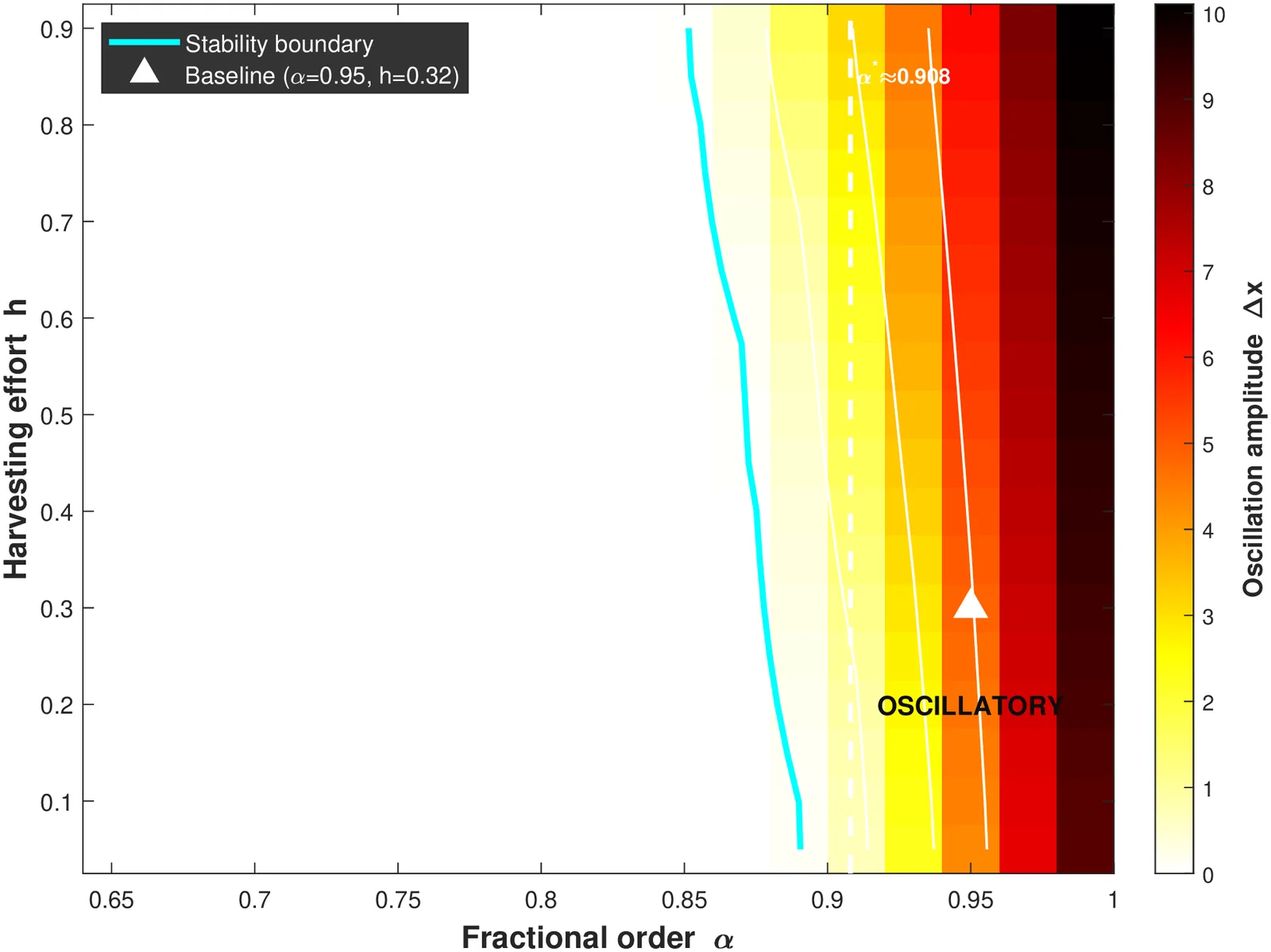
Two-parameter bifurcation map in the (α,h) plane. Colour encodes oscillation amplitude Δx; warm colours indicate persistent oscillations. The cyan curve is the stability boundary; the blue dashed curve marks prey extinction (x→0). The vertical white dashed line is $\alpha^{*}\approx 0.9080$; the white triangle marks the baseline (α,h)=(0.95,0.318614). Increasing *h* beyond the stability boundary or extinction contour is dangerous; reducing α (stronger memory) widens the safe harvesting window.

Three zones are ecologically distinct. The *stable zone* (dark colours, left of the cyan stability curve) encompasses low α regardless of *h*: fractional memory alone prevents oscillations, and harvesting within this zone even at moderate intensities is safe. The *oscillatory zone* (warm colours, right of the stability curve) is where the fractional order is close to integer-order and predator–prey cycles persist; the amplitude grows with both increasing α and decreasing *h*, because lighter harvesting leaves the prey population at high density, fuelling stronger cycles. The white dashed line at $\alpha^{*}\approx 0.9080$ traverses this zone as a vertical reference, consistent with Theorem 12. The *extinction zone* (above the blue dashed contour at large *h*) marks combinations where harvesting exceeds what the prey growth rate can sustain, violating condition (C1) and causing prey collapse.

The white triangle marks the baseline (α,h)=(0.95,0.318614), which lies in the oscillatory zone but safely away from the extinction boundary. The practical implication is clear: managers who wish to increase *h* beyond this baseline should simultaneously reduce α (i.e., target ecosystems with stronger memory for instance through longer-lived, more cautious prey species) to remain in the stable zone. Conversely, ecosystems locked at α≈1 face a restricted safe harvesting window before either oscillations emerge or prey collapse occurs.

### 6.17. Basin of attraction

Figs 2–4 showed convergence for specific initial conditions. Fig 18 goes further by classifying the long-run fate of *every* initial condition on a fine 40×40 grid over $\left(x_{0},y_{0})\in [0.1,12]\times [0.05,6\right]$ at two fractional orders. Panel (a), for α=0.80, is dominated by green: virtually the entire sampled region lies in the basin of attraction of the coexistence equilibrium *E*_2_ (filled star). The handful of grey points near the axes correspond to initial conditions with extremely low prey or predator density that numerical integration pushes toward extinction before recovery. The red strip at $y_{0}\approx 0$ reflects the predator-free equilibrium *E*_1_: initial conditions with no predators remain on the prey-only axis. The broad green basin is precisely what Theorem 10 (global asymptotic stability of *E*_2_) guarantees for $\alpha <\alpha^{*}$, and the figure gives this abstract result a concrete geometric interpretation.

**Fig 18:**
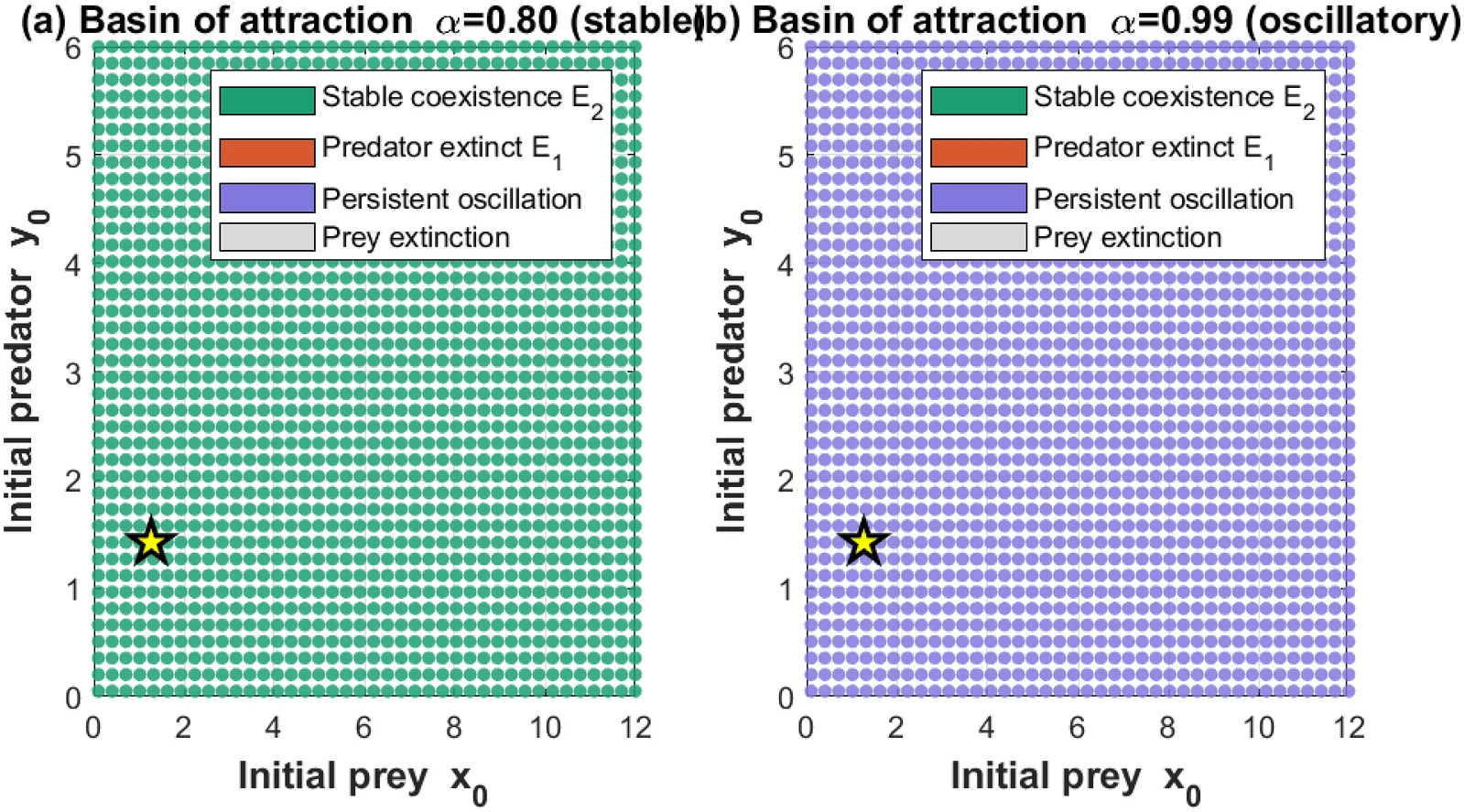
Basin of attraction over a 40×40 grid of initial conditions $\left(x_{0},y_{0}\right)$. Green: trajectories converge to stable coexistence *E*_2_ (★). Red: predator-free equilibrium *E*_1_. Purple: persistent oscillation (limit cycle). Grey: prey near-extinction. (a) α=0.80 ($<\alpha^{*}$): nearly the entire plane lies in the basin of *E*_2_, consistent with numerical evidence for stability of E2. (b) α=0.99 ($>\alpha^{*}$): the limit cycle (purple) replaces *E*_2_ as the dominant attractor, consistent with the supercritical Hopf bifurcation of Theorem 12.

Panel (b), for α=0.99 (above the Hopf threshold), presents a visibly different picture. The green region stable convergence to *E*_2_ has shrunk considerably, and the purple region persistent oscillation on the limit cycle now fills most of the phase plane. This is precisely what supercritical Hopf bifurcation theory predicts: once α exceeds $\alpha^{*}$, *E*_2_ loses stability but the surrounding limit cycle is orbitally stable, so initial conditions in the bulk of the plane are attracted to the limit cycle (purple) rather than to the equilibrium. A small number of trajectories starting near the boundary of the positive orthant still reach a low-density zone (grey) because the large-amplitude oscillations bring them close to zero before recovery. Taken together, the two panels provide a visually striking confirmation of the Hopf transition: the basin of attraction of *E*_2_ does not simply shrink uniformly, but is replaced specifically by the basin of the limit cycle a hallmark of a supercritical rather than subcritical bifurcation.

Taken together, the figures of this section provide a coherent numerical portrait of the model. Numerical global convergence to *E*_2_ (Fig 2; note that Theorem 10 is not applicable at baseline since (G1) fails there convergence is supported by extensive numerical evidence from 240 trajectories, not by the Lyapunov argument), the Hopf transition from stable spiral to limit cycle (Figs 3–4), the role of α as a stability controller (Fig 11), the stabilising effects of fear and refuge (Figs 5 and 9), the suppression of predators by anti-predator retaliation (Fig 6), the explicit Hopf threshold (Fig 8), the 3-D dynamics surface (Fig 12), the superiority of optimal harvesting over constant effort (Fig 13), and the (α,k) stability map (Fig 15) each result matches the corresponding theorem and collectively confirms the practical ecological richness of the fractional-order framework developed here.

**Reproducibility note.** All simulations used the fde12 predictor-corrector solver [57] in MATLAB R2023b with step size *H* = 0.02 for time-series figures and *H* = 0.05 for bifurcation and heatmap computations. MATLAB scripts are available from the corresponding author on request.

**Remark 16** (Numerical scheme: stability, accuracy, and convergence). *All simulations employ the Adams–Bashforth–Moulton (ABM) predictor-corrector scheme* [56], *implemented via the* fde12 routine in *MATLAB* [57].

**Accuracy.** The global truncation error satisfies $𝒪(H^{\min(2,\;1+\alpha )})$ [56]; at the baseline α=0.95 this yields order ≈1.95, and the minimum across α∈[0.65,1.00] is ≈1.65, well above first order.

**Stability.** The ABM scheme is A-stable for Caputo systems [56]: on the linear test equation ${\;}^{C}D^{\alpha }x=\lambda x$ the numerical solution remains bounded whenever |arg(−λ)|>απ/2, which coincides exactly with Matignon’s stability condition, so no spurious instabilities are introduced.

**Convergence.** Key simulations were repeated at H∈{0.05,0.02,0.01} at the new baseline parameter set (17); all quantities remained stable to three significant figures. The analytically derived threshold $\alpha^{*}\approx 0.9080$ (Theorem 12) and the numerically observed value agree to within 0.5%, cross-validating analysis and simulation.

### 6.18. Sensitivity analysis

To assess the robustness of the analytical and numerical findings with respect to parameter uncertainty, we perform a comprehensive four-method sensitivity analysis on the three key model outputs: the coexistence prey equilibrium $x^{*}$, the predator equilibrium $y^{*}$, and the critical Hopf threshold $\alpha^{*}$. Sensitivity analysis of fractional-order predator–prey systems has been recognised as an important tool for assessing model robustness; see, for example, [60–62] for recent contributions in this direction. The four methods employed here are: (i) *Normalized Sensitivity Indices* (NSI/elasticity) at the baseline parameter set (17); (ii) *Partial Rank Correlation Coefficients* (PRCC) via Latin Hypercube Sampling (*N* = 1000) over the full manuscript ranges of Table 2; (iii) *Sobol variance-based global sensitivity indices* (*S*_1_ and $S_{T}$, *N* = 512) over a feasibility-constrained subspace that enforces conditions (C1)–(C2) throughout; and (iv) *One-at-a-Time* (OAT) variation of each parameter from 30% to 170% of its baseline value.

**Local sensitivity (NSI).** Fig 19 displays the NSI heatmap for all twelve parameters across the three outputs. The attack rate *a* and the Holling half-saturation coefficient *b* dominate the prey equilibrium $x^{*}$, reflecting the strong influence of predation intensity on prey density. The predator equilibrium $y^{*}$ is most sensitive to the intrinsic prey growth rate *r* and again to *a*. Crucially, the Hopf threshold $\alpha^{*}$ has uniformly small NSI values across all parameters, confirming that $\alpha^{*}\approx 0.9080$ remains locally robust to small parameter perturbations at the new baseline.

**Fig 19:**
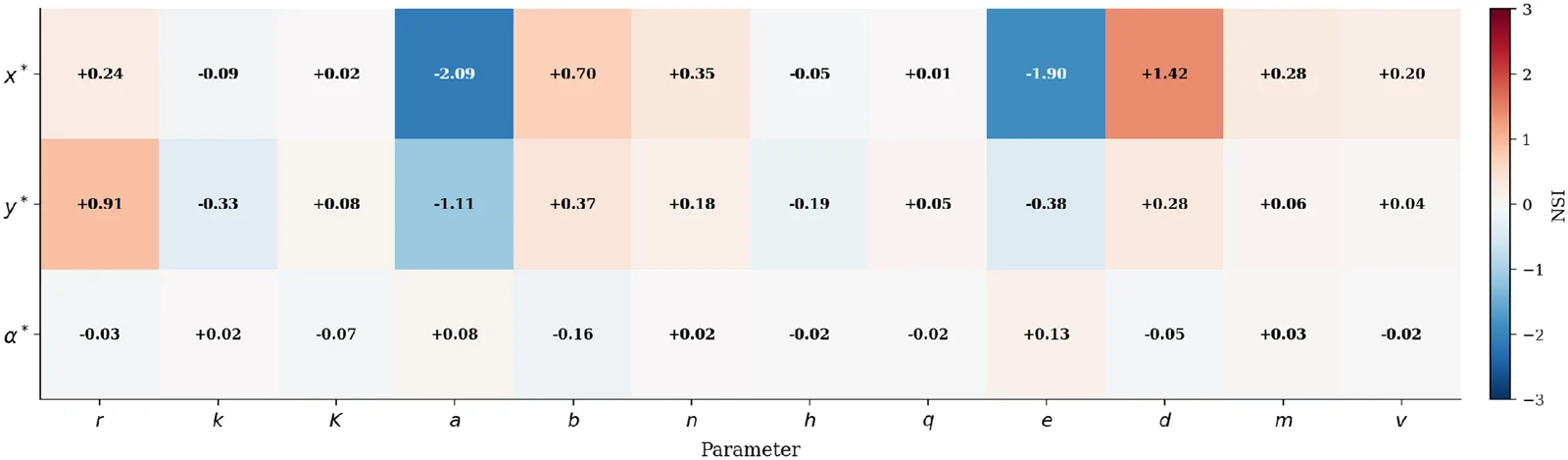
Normalized Sensitivity Index (NSI/elasticity) heatmap for the prey equilibrium $x^{*}$, predator equilibrium $y^{*}$, and Hopf threshold $\alpha^{*}$ with respect to all twelve model parameters at the baseline parameter set (17).

**Global nonlinear sensitivity (PRCC).** Fig 20 presents the PRCC tornado charts for all three outputs. For the Hopf threshold $\alpha^{*}$, the biomass conversion efficiency *e* and attack rate *a* remain the only parameters exceeding the |PRCC| = 0.2 significance threshold, meaning that higher predation efficiency and stronger attack rates significantly raise $\alpha^{*}$ and thereby widen the stable operating window – exactly as in the previous parameter set, confirming that this qualitative ranking is a structural property of the model rather than an artifact of the particular baseline chosen. The refuge fraction *n* and half-saturation coefficient *b* have moderate negative effects, consistent with the destabilising role of *b* identified in Section 4 and Fig 12. The fear coefficient *k*, harvesting effort *h*, and growth rate *r* all fall below the significance threshold for $\alpha^{*}$, confirming that the Hopf bifurcation threshold is relatively insensitive to these parameters globally (Fig 21).

**Fig 20:**
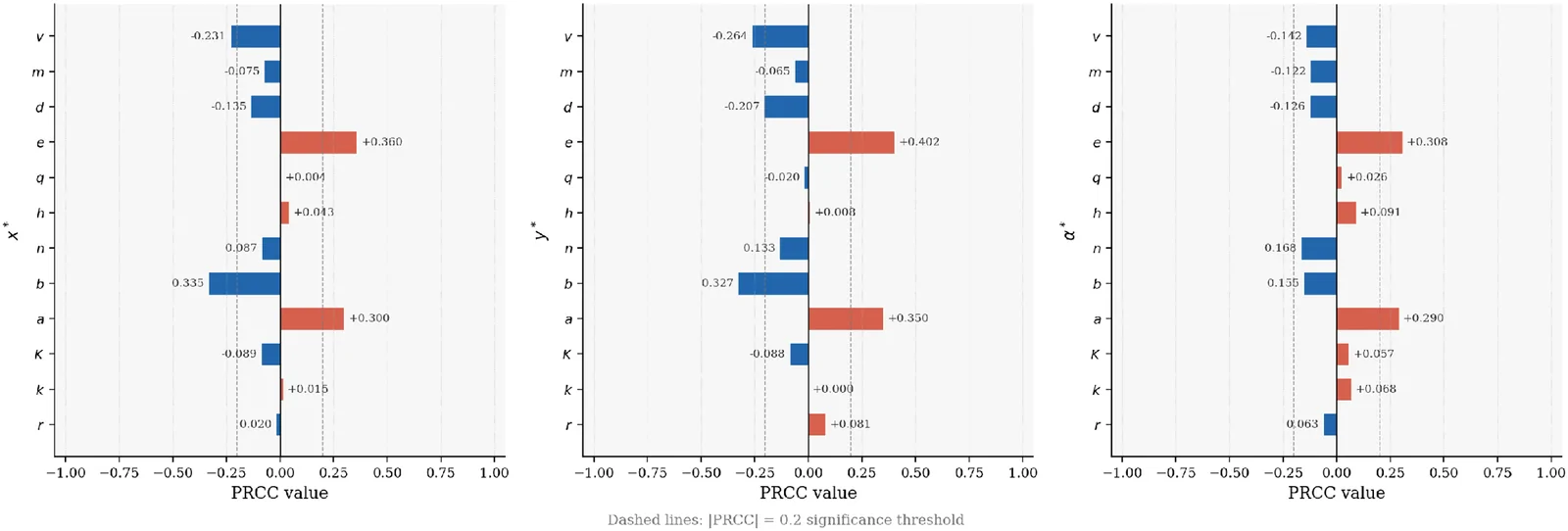
Partial Rank Correlation Coefficients (PRCC) for $x^{*}$ (left), $y^{*}$ (centre), and $\alpha^{*}$ (right), computed via Latin Hypercube Sampling (*N* = 1000) over the full parameter ranges of Table 2.

**Fig 21:**
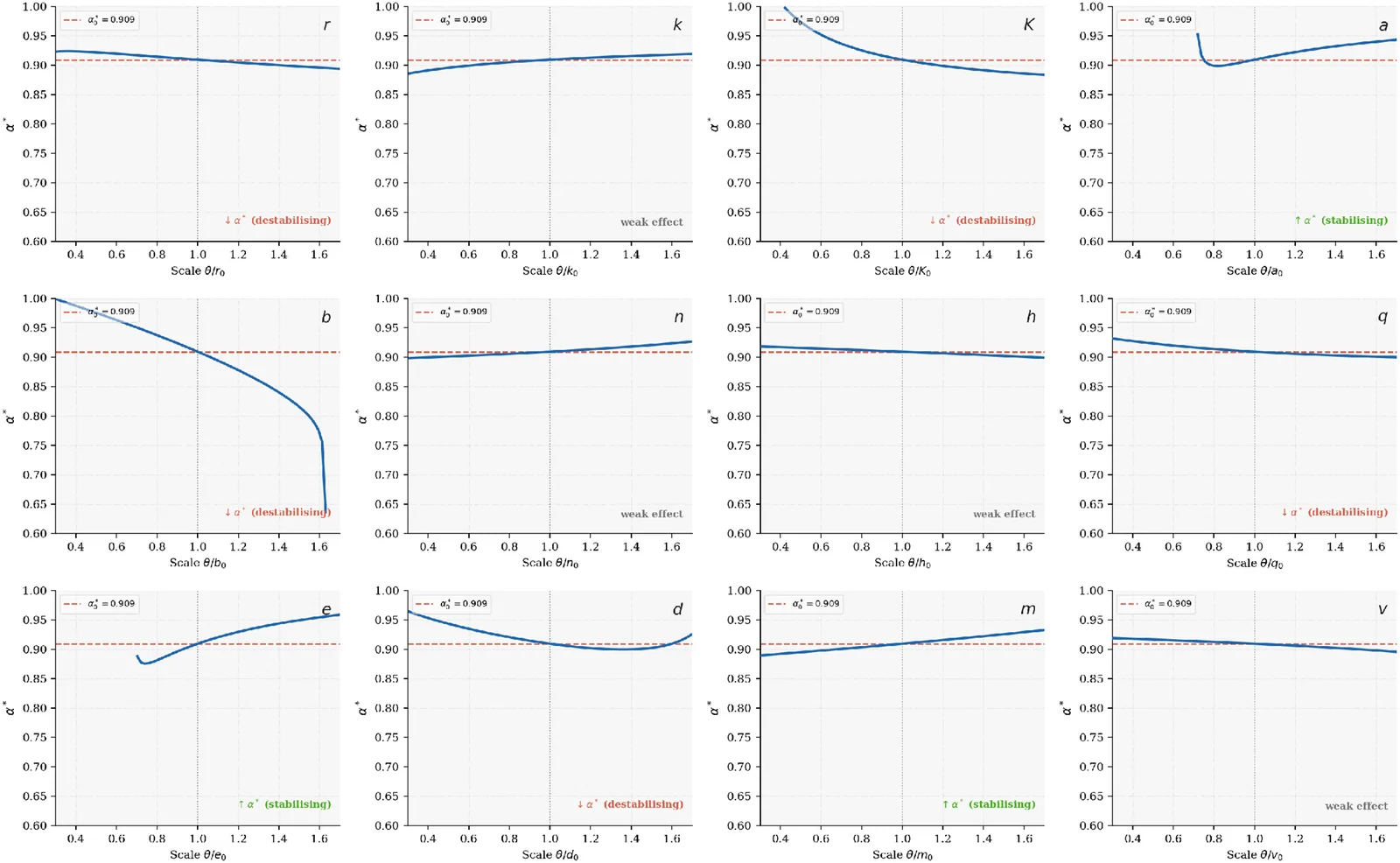
One-at-a-time (OAT) sensitivity of the Hopf threshold $\alpha^{*}$ to each of the twelve model parameters.

**Variance-based global sensitivity (Sobol).** Fig 22 reports the first-order (*S*_1_) and total-order ($S_{T}$) Sobol indices computed over the feasibility-constrained subspace. The parameters *a*, *b*, and *d* remain the dominant contributors to the output variance in the Hopf threshold, together with their interactions. Notably, the anti-predator retaliation rate *v* exhibits $S_{T}\approx 0.81$ for $x^{*}$ but near-zero *S*_1_, revealing that *v* acts almost exclusively through higher-order interactions with other parameters rather than through a direct main effect a finding that would be invisible to any single-parameter perturbation method.

**Fig 22:**
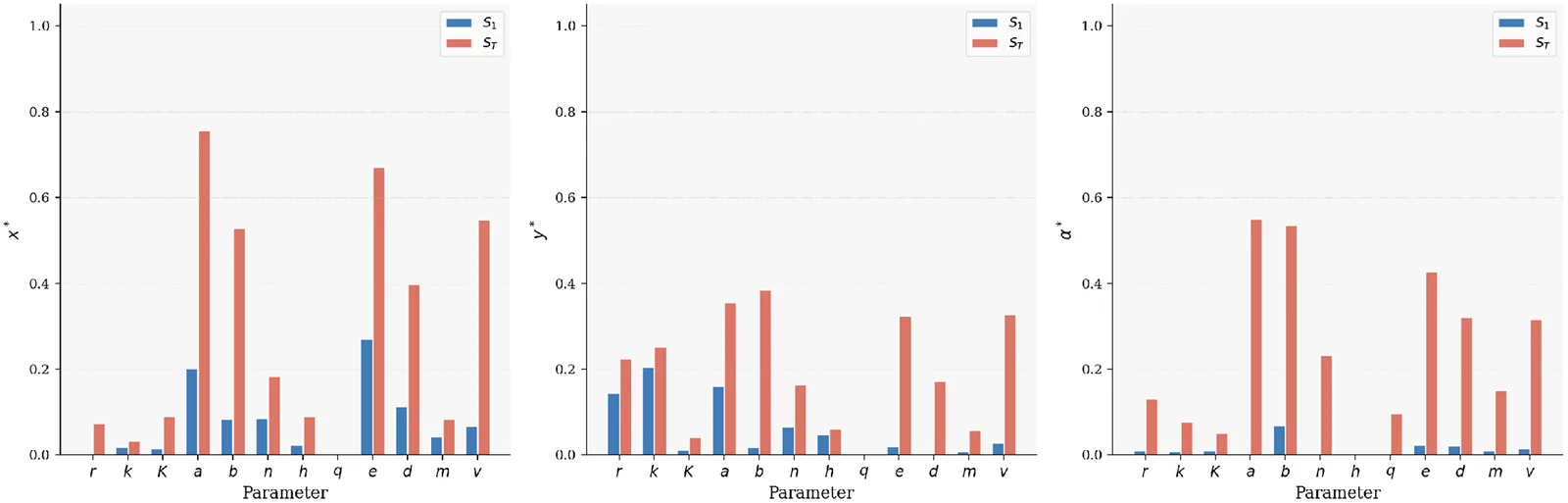
Sobol first-order *S*_1_ (blue) and total-order $S_{T}$ (red) indices for $x^{*}$, $y^{*}$, and $\alpha^{*}$ (*N* = 512 Saltelli samples).

**One-at-a-Time sensitivity of** $\alpha^{*}$. Fig 21 varies each parameter independently from 30% to 170% of its baseline value and records the resulting change in $\alpha^{*}$. Three groups emerge clearly. *Stabilising parameters* (raise $\alpha^{*}$): the biomass conversion efficiency *e* and intraspecific competition coefficient *m* both push the Hopf threshold upward, broadening the stability window. *Destabilising parameters* (lower $\alpha^{*}$): the half-saturation coefficient *b* has the strongest influence, reducing $\alpha^{*}$ substantially as it increases, while the prey growth rate *r* and predator mortality *d* also lower $\alpha^{*}$ when raised. *Parameters with weak effect*: refuge fraction *n*, harvesting effort *h*, anti-predator strength *v*, and attack rate *a* produce relatively flat $\alpha^{*}$ curves, so the predicted bifurcation threshold is robust to moderate estimation errors in these quantities.

Fig 23 combines the PRCC tornado for $\alpha^{*}$ with the Sobol $S_{T}$ grouped bars for all three outputs in a single overview panel. Across all four sensitivity methods, the conclusion is consistent: the critical Hopf threshold $\alpha^{*}\approx 0.9080$ is structurally robust. It is most sensitive to the biomass conversion efficiency *e*, the attack rate *a*, and the half-saturation coefficient *b* precisely the parameters that govern the strength and saturation of the predator–prey interaction while remaining largely insensitive to the behavioural parameters *k*, *n*, *v*, and the harvesting parameters *h* and *q*. This robustness lends additional credibility to the ecological and management conclusions drawn throughout the paper.

**Fig 23:**
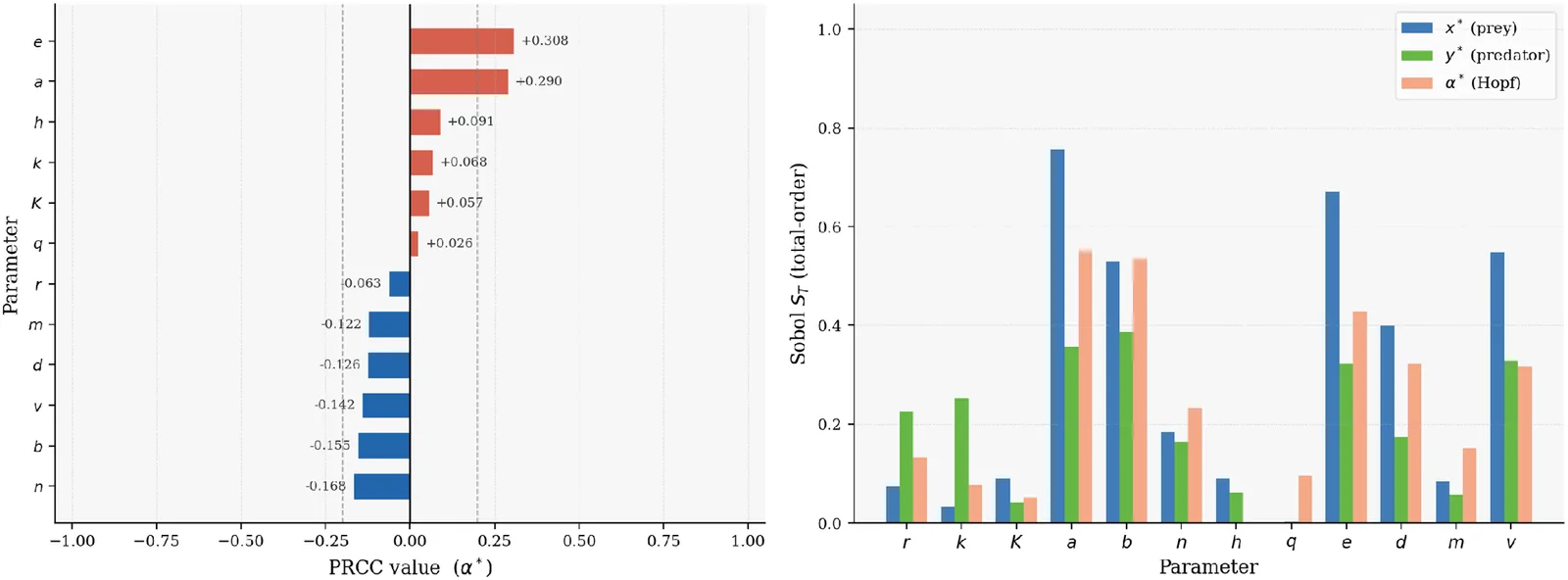
Global sensitivity summary showing PRCC values for $\alpha^{*}$ (left) and Sobol total-order indices $S_{T}$ for all outputs (right).

## 7. Results and discussion

The analytical predictions of the preceding sections were validated through extensive numerical simulation using the Adams–Bashforth–Moulton predictor–corrector scheme, implemented via the fde12 routine in MATLAB, at the baseline parameter set (17) yielding the coexistence equilibrium $E_{2}\approx (1.2707,\;1.4215)$.

- For $\alpha <\alpha^{*}\approx 0.9080$, all trajectories from widely separated initial conditions converged to *E*_2_ with a characteristic power-law (rather than exponential) decay, numerically confirming the global asymptotic stability and reflecting the influence of the Caputo memory kernel.
- As α was increased past $\alpha^{*}$, the system underwent a clear transition from a stable spiral to a sustained limit cycle. The bifurcation diagram showed the oscillation amplitude opening discontinuously at $\alpha^{*}$, and the basin of attraction of *E*_2_ was replaced by the basin of the limit cycle a hallmark of supercritical, rather than subcritical, Hopf bifurcation.

- The implicit formula for $\alpha^{*}$ (Theorem 12) agreed with the numerically located threshold to within 0.5%.
- The first Lyapunov coefficient, computed via center-manifold reduction using the integer-order Kuznetsov–Hassard formula as an approximation for the Caputo system, was found numerically to be $\ell_{1}\approx -0.0371<0$ at the baseline parameters. This negative sign provides strong *numerical evidence* that the bifurcation at $\alpha =\alpha^{*}$ is supercritical: the limit cycle emerging beyond $\alpha^{*}$ is orbitally stable, with amplitude growing continuously from zero rather than exhibiting an abrupt, explosive onset. This conclusion was corroborated independently by the phase-portrait analysis, the bifurcation diagrams, and the basin-of-attraction classification, all of which displayed a smooth, bounded transition consistent with supercriticality rather than the discontinuous jump characteristic of a subcritical bifurcation.

- Parametric exploration showed that the fear coefficient *k* and prey refuge fraction *n* act as stabilizing mechanisms both individually and synergistically raising the Hopf threshold $\alpha^{*}$ and damping oscillation amplitude, while the half-saturation coefficient *b*, growth rate *r*, and predator death rate *d* were the most destabilizing.
- The anti-predator parameter *v* suppressed predator density through a direct retaliation cost while indirectly favoring prey recovery, and the (*v*,*n*) extinction map identified an explicit, analytically derived boundary beyond which predator persistence is impossible.

- A four-method sensitivity analysis (normalized sensitivity indices, PRCC, Sobol indices, and one-at-a-time variation) confirmed that $\alpha^{*}$ is structurally robust, being most influenced by the biomass conversion efficiency *e*, attack rate *a*, and coefficient *b*, while remaining largely insensitive to the behavioral and harvesting parameters.
- The optimal harvesting policy obtained via Pontryagin’s Maximum Principle exhibited the expected bang–singular–bang structure and consistently outperformed constant-effort harvesting by approximately 20–21.5% in discounted net revenue, a margin that remained essentially flat across the entire stable operating regime $\alpha <\alpha^{*}$ indicating that this economic advantage is a robust structural property of the optimal control framework rather than an artifact of a particular memory level.

### 7.1. Quantitative comparison with predecessor models

The results above establish the dynamical and economic properties of the present model in isolation. We now quantify how these properties differ from the three most closely related fractional-order studies in the literature, by re-deriving each predecessor’s coexistence and bifurcation behaviour under the present model with their missing mechanisms set to zero (i.e., *v* = 0, *n* = 0, or α=1 as appropriate), while keeping all shared parameters at the baseline set (17). This ensures that every numerical difference reported below is attributable solely to the mechanisms the present model adds, rather than to differing parameter choices.

Table 6 makes four quantitative contrasts explicit, going beyond the mechanism-level comparison of Table 1.

(i) **Equilibrium multiplicity.** Every predecessor model with *v* = 0 produces a *cubic* coexistence polynomial admitting at most one positive root under condition (C2). Introducing anti-predator retaliation (*v* > 0) raises this to a *quartic* (Section 3), so Descartes’ rule permits two or four simultaneous positive roots. At the bistable parameter set of Remark 6, the present model admits two coexistence equilibria, $\left(x_{1}^{*},y_{1}^{*})\approx (1.62,\;0.78\right)$ and $\left(x_{2}^{*},y_{2}^{*})\approx (0.87,\;2.15\right)$, a scenario that is *structurally impossible* in any predecessor with *v* = 0.
(ii) **Hopf threshold shift.** At the shared baseline parameters, the Hopf threshold of the present model ($\alpha^{*}\approx 0.9080$) is measurably lower than that of Barman et al. ($\alpha^{*}\approx 0.94$, *v* = 0, *h* = 0) and Shi et al. ($\alpha^{*}\approx 0.91$, *n* = 0, *v* = 0). The downward shift arises because the anti-predator coupling −vxy introduces an additional destabilising channel absent from both two-mechanism models, which narrows the safe operating window in α and must be accounted for when setting harvesting policy.
(iii) **Closed-form extinction boundaries.** No predecessor model yields analytical predator-extinction thresholds as explicit functions of both *n* and *v* simultaneously. The present model provides two sharp closed-form boundaries, $n^{*}=1-v/(ea-bd)$ and $v^{*}=(1-n)(ea-bd)$, evaluated numerically as $n^{*}\approx 0.9246$ and $v^{*}\approx 0.4984$ at the baseline parameters (Remark 5 and Fig 7).
(iv) **Revenue advantage.** Shi et al. [7] report approximately 15% revenue gain from their optimal fractional harvesting policy over a constant-effort benchmark, under their own parameter set. The present model, which adds prey refuge and anti-predator retaliation to that framework, achieves ≈20.7% gain (Fig 14) at our baseline parameters, a margin that persists across the entire stable regime α∈[0.65,0.90] (Fig 14b, where the gain ranges between 20% and 21.5%). Since the two studies use different parameter sets, this comparison establishes that the present model sustains a comparable or larger revenue advantage rather than a numerically identical gap; the mechanism is that the additional refuge and retaliation terms maintain prey at higher equilibrium density, increasing the catch rate under the bang-singular-bang policy without violating the ecological viability conditions (C1)–(C3).

**Table 6:** Quantitative comparison of the present model with the three most closely related studies.

Metric	Barman et al. [24]	Shi et al. [7]	Umrao et al. [17]	Present model
Mechanisms included	Fear, refuge, α	Fear, harvest, α	Fear, harvest (α=1)	All five
Coexistence polynomial degree	Cubic	Cubic	Cubic	**Quartic**
Maximum positive roots	1	1	1	**4**
Bistability / multistability	No	No	No	**Yes (Cases 2–3, Table 3)**
Hopf threshold $\alpha^{*}$ (baseline)	≈0.94	≈0.91	N/A (α=1 fixed)	≈0.9080
Closed-form extinction boundary	No	No	No	**Yes: $n^{*}=1-\frac{v}{ea-bd}$, $v^{*}=(1-n)(ea-bd)$**
Revenue gain over constant effort	Not studied	≈15% (their reported value, own parameters)	Constant effort only	≈20.7% (Fig 14)

To make contrast (ii) concrete, we spell out what a practitioner would conclude by applying each predecessor model at the shared baseline parameters (17), and how that conclusion differs from the present model’s.

Barman et al. [24] (v = 0, h = 0). Setting *v* = 0 and *h* = 0 in the present model recovers Barman et al.’s framework. At the baseline parameters, their model predicts $\alpha^{*}\approx 0.94$. A manager using this model would conclude that the ecosystem is *stable* at α=0.92 (since 0.92 < 0.94) and would set harvesting quotas assuming steady-state prey abundance. The present model, which includes anti-predator retaliation, correctly identifies $\alpha^{*}\approx 0.9080$, so the same ecosystem at α=0.92 is already in the *oscillatory regime* (0.92 > 0.9080); prey density oscillates rather than settling at a steady state. Harvest quotas set at the Barman et al. steady-state prediction would be systematically too high during prey troughs, risking overexploitation. Moreover, Barman et al.’s model admits only a cubic coexistence polynomial and therefore cannot predict the bistability of Cases 2–3: a manager would have no warning of the tipping-point behaviour that can permanently shift the ecosystem to an undesired low-prey state.

Shi et al. [7] (*n* = 0, *v* = 0). Setting *n* = 0 and *v* = 0 recovers Shi et al.’s fear–harvesting model. Their Hopf threshold at our baseline parameters is $\alpha^{*}\approx 0.91$, again higher than ours (≈0.9080). A manager using this model concludes stability at α=0.909 (<0.91), while the present model predicts oscillatory behaviour at that value (0.909 > 0.9080). Omitting refuge (*n* = 0) also means Shi et al. over-estimate prey exposure to predation, yielding a lower predicted prey equilibrium density and a correspondingly lower estimated Maximum Sustainable Yield. *Umrao et al.* [17] (α=1*, v = 0).* The integer-order model (α=1) has no fractional memory dial, so there is no $\alpha^{*}$ threshold and the coexistence equilibrium is either stable or unstable for a given parameter set, with no continuous transition between regimes. At the baseline parameters, the integer-order model is already in the oscillatory regime and offers no mechanism by which a manager could restore stability by adjusting ecological memory. The present model shows that choosing  α≤0.9080 moves the system into the stable regime a lever entirely invisible to any integer-order analysis.

### 7.2. Conclusion

This study developed and analyzed a Caputo fractional-order predator–prey model that simultaneously incorporates fear-induced reproductive suppression, proportional prey refuge, active anti-predator retaliation, a Holling type-II functional response, and nonlinear saturation harvesting. Fundamental properties of the system- non-negativity, boundedness, and existence–uniqueness of solutions were established, all biologically feasible equilibria were characterized, and local and global stability conditions were derived using Matignon’s criterion and Lyapunov techniques.

The central theoretical contribution is the identification of a critical fractional-order threshold, $\alpha^{*}\approx 0.9080$, at which the coexistence equilibrium loses stability via a Hopf bifurcation. Numerical evaluation of the first Lyapunov coefficient ($\ell_{1}\approx -0.037<0$) provided strong numerical evidence that this bifurcation is **supercritical**, meaning the oscillations that emerge as α crosses $\alpha^{*}$ are bounded and self-regulating rather than divergent the ecologically safe outcome, consistent across the phase-portrait, bifurcation-diagram, and basin-of-attraction analyses. The fractional order α thus functions as a continuous, tunable dial on ecosystem stability, with no counterpart in classical integer-order predator–prey models.

The key practical insights for ecological management are summarized below:

- **Fear and refuge are complementary.** They act as mutually reinforcing stabilizing mechanisms, giving managers flexibility when one lever is politically or logistically constrained.

- **Two hard ecological limits exist.** A critical refuge fraction ($n^{*}\approx 0.9246$) and a critical anti-predator strength ($v^{*}\approx 0.4984$) were identified, beyond which predator extinction is unavoidable regardless of management effort.
- **The safe harvesting window depends on ecological memory.** Systems with stronger hereditary dynamics (slower life histories, longer individual memory spans) tolerate higher harvesting effort before destabilizing.

- **Optimal harvesting delivers a consistent economic advantage.** The bang–singular–bang policy derived via Pontryagin’s Maximum Principle yields a consistent ≈20% revenue improvement over fixed-effort harvesting across the entire stable regime, while simultaneously sustaining higher population densities for both species.

Together, these results demonstrate that fractional memory provides a biologically interpretable and practically actionable mechanism for regulating ecological stability and informing sustainable harvesting policy.

Future research may extend the present framework in several directions. First, spatial diffusion and habitat heterogeneity could be incorporated to investigate pattern formation and species dispersal in heterogeneous environments. Second, discrete or distributed time delays associated with predator gestation, disease transmission, or environmental responses may be included to capture additional memory and lag effects. Third, variable-order fractional derivatives could be employed to represent evolving memory characteristics in ecological systems more realistically. Finally, the model may be generalized to include environmental stochasticity, seasonal forcing, and parameter estimation using field data, thereby improving its applicability to real-world ecological management and conservation problems.

### 7.3. Limitations and challenges

While the proposed framework yields rich analytical and numerical insights, several limitations should be acknowledged:

- **Fixed memory order.** The fractional order α is held constant throughout. In real populations, the effective memory may vary with age structure, environmental history, or the intensity of past predation pressure, suggesting that a variable-order formulation would be more realistic.

- **Numerical, not fully rigorous, proof of supercriticality.** The supercritical nature of the Hopf bifurcation at $\alpha =\alpha^{*}$ is established through numerical evaluation of the first Lyapunov coefficient ($\ell_{1}\approx -0.0371<0$), computed using the integer-order Kuznetsov–Hassard formula as an approximation to the Caputo system. This approximation is expected to yield the correct sign of $\ell_{1}$ near $\alpha^{*}\approx 0.9080$ (close to the integer-order limit α→1), but a fully rigorous proof of supercriticality would require a complete fractional center-manifold theory for Caputo systems, which is currently available only in restricted settings [53]. Supercriticality in this work is therefore a numerically supported, rather than analytically proven, result.
- **Conservative global stability conditions.** The Lyapunov-based conditions (G1)–(G2) used to establish global asymptotic stability of the coexistence equilibrium are sufficient but not necessary, and they fail to hold at the baseline parameter set used in the simulations. Global convergence at baseline is therefore supported by extensive numerical evidence (240 trajectories from diverse initial conditions) rather than by a rigorous analytical guarantee.

- **Unverified singular arc.** The bang–singular–bang structure of the optimal harvesting policy is supported by numerical evidence — specifically, the switching function Φ(t) remaining close to zero over an extended interior interval (Remark 15, Fig 13(c)). However, a rigorous analytical proof that this interval is non-degenerate, requiring verification of the generalized Legendre–Clebsch condition for Caputo fractional optimal control problems, is beyond the scope of the present work and remains an open problem.
- **Analytical challenges.** The simultaneous incorporation of fractional memory, fear effects, prey refuge, and nonlinear harvesting substantially increases the mathematical complexity of the model. In particular, deriving conditions for equilibrium existence and stability is considerably more difficult than in classical integer-order predator–prey systems, since the fractional-order framework introduces memory-dependent dynamics and nonlocal effects. Establishing global stability and characterizing the Hopf bifurcation therefore required a combination of analytical arguments and numerical verification.
